# Single cell variability of CRISPR‐Cas interference and adaptation

**DOI:** 10.15252/msb.202110680

**Published:** 2022-04-25

**Authors:** Rebecca E McKenzie, Emma M Keizer, Jochem N A Vink, Jasper van Lopik, Ferhat Büke, Vera Kalkman, Christian Fleck, Sander J Tans, Stan J J Brouns

**Affiliations:** ^1^ Department of Bionanoscience Delft University of Technology Delft The Netherlands; ^2^ Kavli Institute of Nanoscience Delft The Netherlands; ^3^ AMOLF Amsterdam The Netherlands; ^4^ Biometris, Department of Mathematical and Statistical Methods Wageningen University Wageningen The Netherlands; ^5^ Freiburg Center for Data Analysis and Modeling (FDM) Spatial Systems Biology Group University of Freiburg Freiburg Germany

**Keywords:** agent‐based simulations, single‐cell analysis, spacer acquisition, time‐lapse microscopy, type I CRISPR‐Cas, Microbiology, Virology & Host Pathogen Interaction

## Abstract

While CRISPR‐Cas defence mechanisms have been studied on a population level, their temporal dynamics and variability in individual cells have remained unknown. Using a microfluidic device, time‐lapse microscopy and mathematical modelling, we studied invader clearance in *Escherichia coli* across multiple generations. We observed that CRISPR interference is fast with a narrow distribution of clearance times. In contrast, for invaders with escaping PAM mutations we found large cell‐to‐cell variability, which originates from primed CRISPR adaptation. Faster growth and cell division and higher levels of Cascade increase the chance of clearance by interference, while slower growth is associated with increased chances of clearance by priming. Our findings suggest that Cascade binding to the mutated invader DNA, rather than spacer integration, is the main source of priming heterogeneity. The highly stochastic nature of primed CRISPR adaptation implies that only subpopulations of bacteria are able to respond quickly to invading threats. We conjecture that CRISPR‐Cas dynamics and heterogeneity at the cellular level are crucial to understanding the strategy of bacteria in their competition with other species and phages.

## Graphical abstract

Time‐lapse microscopy combined with computational modeling reveals new insights into the single‐cell biology of CRISPR‐Cas defense during invader DNA clearance in *E*. *coli*.

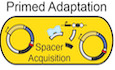

## Introduction

During the last decade, important progress has been made in identifying the sequence of steps and molecular interactions required for successful adaptive immunity by the model type I‐E CRISPR‐Cas system (Datsenko *et al*, 2012; Swarts *et al*, 2012; Nuñez *et al*, 2015; Künne *et al*, 2016; Dillard *et al*, 2018; Loeff *et al*, 2018; Musharova *et al*, 2019; Xue & Sashital, 2019; Kim *et al*, 2020; Vink *et al*, 2020). CRISPR (clustered regularly interspaced short palindromic repeats) immunity involves three main stages beginning with the acquisition of a spacer, a small piece of DNA derived from a foreign invader and stored in the CRISPR array for future defence (Bolotin *et al*, 2005; Barrangou *et al*, 2007). This array is then transcribed and processed into small CRISPR RNAs (crRNAs), which guide a surveillance complex, formed from a number of Cas (CRISPR‐associated) proteins, towards the invader DNA (Brouns *et al*, 2008; Jackson *et al*, 2014). For type I‐E systems, a 5′‐CTT consensus PAM (protospacer adjacent motif) sequence flanking the targeted site of the invader (Deveau *et al*, 2008; Mojica *et al*, 2009) allows swift recognition and ultimately degradation of the invader, through a process called direct interference (Garneau *et al*, 2010; Westra *et al*, 2012; Leenay *et al*, 2016; Xue & Sashital, 2019). However, invaders can escape direct interference via mutation within the seed region of the target site or PAM (Deveau *et al*, 2008; Semenova *et al*, 2011; Fineran *et al*, 2014). In response, the I‐E system can initiate priming, which promotes accelerated acquisition of new spacers due to a pre‐existing partial match to the invader (Datsenko *et al*, 2012; Swarts *et al*, 2012). Primed adaptation is much faster than naïve adaptation (preprint: Stringer *et al*, 2020) and is required for the insertion of a new matching spacer with a consensus PAM allowing subsequent invader degradation, which we here refer to as primed interference.

At the level of individual cells, however, much more is unknown. Interference is a kinetic arms race between invader replication and degradation, which could result in complex and stochastic dynamics within single cells. Replication and degradation themselves may also display variability between cells in the population. For instance, invader degradation rates can be affected by stochastic processes such as the expression of CRISPR‐Cas components, target localization and nuclease recruitment (Semenova *et al*, 2016; Vink *et al*, 2020). Priming also depends on many processes in which the dynamical interplay is unclear, including the production of suitable fragments of DNA for spacer acquisition (pre‐spacers), the assembly of adaptation complexes required for further spacer selection and the processing and insertion of these pre‐spacers into the CRISPR array (Nuñez *et al*, 2015; Jackson *et al*, 2017; Wright *et al*, 2017; Kim *et al*, 2020). Elucidating the cellular dynamics and heterogeneity of the CRISPR‐Cas response is critical to understanding interference and adaptation mechanistically and of direct importance to its natural function. For instance, upon invasion, cells are thought to have a limited time window to respond in order to escape invader replication, protein production and cell death (Davison, 2015; Shao *et al*, 2015; Kutter *et al*, 2018; Hampton *et al*, 2020).

A number of studies have investigated the interference process by collecting either population averages, or single‐cell data on short time scales (<1 s) (Fineran *et al*, 2014; Xue *et al*, 2015; Staals *et al*, 2016; Jackson *et al*, 2019; Musharova *et al*, 2019; Vink *et al*, 2020). However, averaging within a population can conceal the variation between cells and the dynamics within single cells over time (Spudich & Koshland, 1976; Elowitz, 2002), thus masking the underlying dynamics of CRISPR‐Cas interference. In addition, investigations into the adaptation process have provided great insight into the diversity of spacers acquired (Staals *et al*, 2016; van Houte *et al*, 2016), possible mechanisms of target destruction (Datsenko *et al*, 2012; Richter *et al*, 2014) and conditions under which adaptation most frequently occurs within a population (Díez‐Villaseñor *et al*, 2013; Amlinger *et al*, 2017; Høyland‐Kroghsbo *et al*, 2018); however, these studies could not observe any variation existing in each step of the adaptation process within individual cells.

Recently, developments in the field have begun to include the use of time‐lapse microscopy to investigate invader establishment and degradation in single cells (Guan *et al*, 2017; preprint: Mamontov *et al*, 2021). Here, we set out to further these techniques and investigate and quantify the dynamics and variability of both the interference and adaptation processes in single‐cell lineages. Using time‐lapse microscopy and microfluidic devices, we followed individual cells over multiple rounds of division while simultaneously monitoring CRISPR‐Cas protein expression and DNA degradation. Hence, we obtained individual lineages, the genealogical relations between them, as well as real‐time data on the DNA clearance process, instantaneous growth rates, cell sizes and division frequencies of individual cells. We determined that while direct interference occurs quickly and consistently, clearing the target from all cells within hours, priming is highly variable and much slower, taking over several tens of hours for some cells. Further, through stochastic agent‐based modelling we were able to define the adaptation and clearance stages of priming and identified primed adaptation as the source of the variation observed—more specifically the binding of Cascade to the mutated target DNA, rather than other complex processes including the integration of new spacer DNA fragments into the host genome.

## Results

### Time‐lapse microscopy of the CRISPR‐Cas response

**Figure 1:**
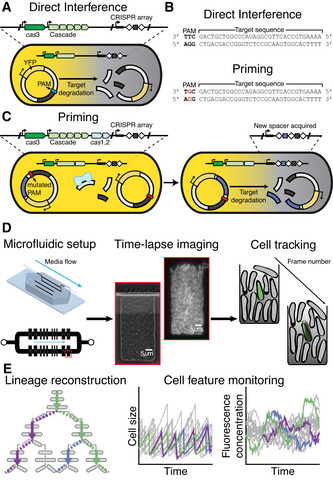
Investigating single‐cell behaviour during CRISPR‐Cas defence using time‐lapse microscopy Schematic of the direct interference process. The cell contains a I‐E CRISPR‐Cas system controlled by the lacUV5 (*cas*3) and araBp8 (*cas* operon) inducible promoters (black arrows), as well as the CRISPR array with a single spacer targeting the plasmid (grey box). The plasmid encodes YFP and contains a sequence matching the spacer (grey), flanked by a consensus PAM (blue). Immediate targeting by the CRISPR‐Cas system results in degradation of the plasmid and loss of the YFP in the cell.To invoke priming, the 5′‐CTT consensus PAM, flanking the target sequence located on the plasmid, is mutated by one nucleotide to a non‐consensus PAM 5′‐CGT.Schematic of the priming process. (Left cell) A mutation of the PAM (red) flanking the target sequence means the spacer in the CRISPR array can no longer initiate direct interference. Fragments in the cell can be captured and processed by Cas1,2 (light blue). (Right cell) The Cas1,2 complex integrates the fragment into the CRISPR array as a new spacer (purple), which matches the target plasmid and results in degradation and loss of YFP in the cell.To allow long‐term imaging, cells are grown in a microfluidic chip that allows constant media supply. Cells within a single well are imaged frequently in phase contrast and fluorescence allowing segmentation and tracking of lineage history across frames.Variation in features of reconstructed single‐cell lineages (left) such as size (middle) and fluorescence concentration (right) are continuously monitored enabling further investigation.

Using two strains, KD615 (WT) and KD635 (Δ*cas*1,2) (Appendix Table S1), we investigated priming and direct interference, respectively. The strains contain an array with a leader, two repeats and a single previously characterised spacer, spacer8 (SP8) (Swarts *et al*, 2012; Musharova *et al*, 2019) (Fig 1A–C). In addition, these strains are engineered to control *cas* gene expression using arabinose and IPTG induction, and hence initiation of the CRISPR‐Cas response. Target plasmids were engineered to encode a constitutively expressed YFP or CFP fluorescent protein (Kremers *et al*, 2006) and contain a target sequence that is complementarity to SP8 in the CRISPR array, allowing direct monitoring of target DNA presence in individual cells over time (Fig 1A–C) (Appendix Table S1). In order to investigate the direct interference process, we flanked the target sequence with a 5′‐CTT consensus PAM (Mojica *et al*, 2009) (Fig 1A and B). Further, to investigate the priming response we mutated the PAM to 5′‐CGT (Fig 1B and C), a mutation known to allow mobile genetic elements (MGE) to escape interference and invoke a primed adaptation response (Semenova *et al*, 2011; Datsenko *et al*, 2012; Musharova *et al*, 2019).

The use of a microfluidic device (Wehrens *et al*, 2018) enabled fluorescence time‐lapse imaging for over 36 h with the option for media exchange (Fig 1D). The device contained chambers allowing observation of a single layer of cells, constant medium supply, removal of cells that no longer fit the chamber due to growth and control of intracellular processes via induction. Image analysis software was used to segment and track all cells and their fluorescence signals, thus allowing the reconstruction of lineage trees in a defined region at the bottom of the chamber (Fig 1D and E) (Young *et al*, 2012; Kiviet *et al*, 2014; Wehrens *et al*, 2018).

### Direct interference is fast and synchronous

**Figure 2:**
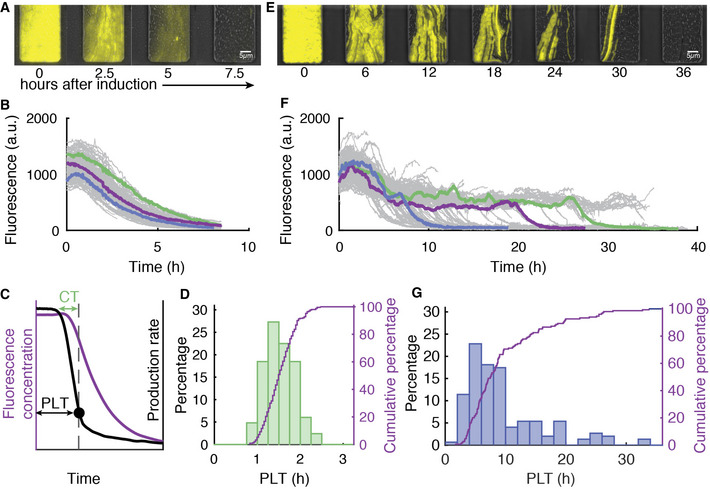
Variation in target plasmid clearance times is much larger when CRISPR adaptation is required A, BClearance of a target with a consensus PAM by direct interference. Overlay of fluorescent and phase contrast time‐lapse images. The presence of the target plasmid is tracked by its YFP production (A). Reconstructed lineage traces of the imaged population (A) from induction of the CRISPR‐Cas system over time (grey) lineages show some variation in plasmid clearance times (coloured) (B).CProduction rate (black line) of the YFP is used to determine the plasmid loss time, PLT, (black dot, dashed line) which is the time from induction until detection of loss (black arrow) allowing earlier detection than using the mean fluorescence (purple line). The time from first targeting of a single plasmid to the PLT (dashed line) is defined as the clearance time (CT, purple arrow).DDistribution of PLTs determined by the production rate during direct interference (*n* = 250 loss events).E, FClearance of a matching target with a mutated PAM via priming. Overlay of fluorescent and phase contrast time‐lapse images as in (A). Reconstructed lineage traces of the imaged population (E) (grey). Lineages show large variations in the time taken to clear the plasmid (coloured) (F).GDistribution of PLTs via priming calculated with the production rate (*n* = 149 loss events). Source data are available online for this figure.

**Figure EV1:**
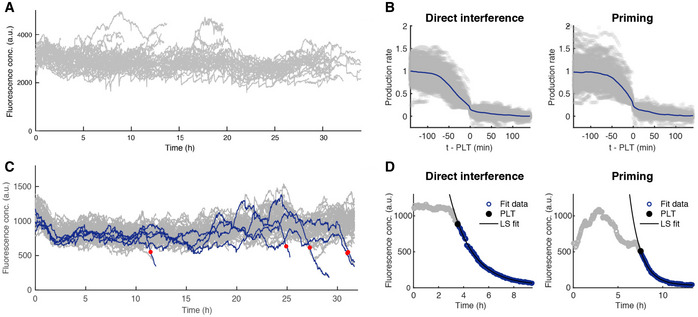
Details of CRISPR‐dependent plasmid loss and subsequent fluorescence decay Plasmid loss is CRISPR‐dependent. The YFP fluorescence traces in arbitrary units (a.u.) of the WT strain harbouring pControl a plasmid with no target for the CRISPR‐Cas system. Time‐lapse imaging was carried out for 35‐h post‐induction of the *cas* genes and showed no plasmid loss.Decay of YFP fluorescence in both direct interference and priming follows exponential decay. The fluorescence concentration data (open circles) of representative lineage of the direct interference (left) and priming (right) lineages can be described by exponential decay. This was evaluated by performing a least‐squares (LS) fit of the fluorescence concentration data (purple open circles) after the moment of plasmid loss (PLT, black circle) to an exponential curve (LS fit, black line).In the absence of Cas1 and Cas2 clearance of a target with a non‐consensus PAM mutant occurs rarely. The YFP fluorescence of the ∆*cas1,2* strain containing pMutant was imaged for 34 h after induction. Lineages that were able to clear the plasmid are highlighted in blue with the red dot indicating the PLT determined by the production rate. 1.4% of lineages (5 unique events, red dot) cleared the plasmid.Plasmid loss during direct interference and primed interference processes occur on a comparable timescale. All production rate traces (grey) starting from 140 min prior to the detected plasmid loss time PLT from direct interference (left) and priming (right) were aligned at the PLT (t‐PLT = 0) and the average trend (navy) normalised for comparison. From the average trend, we estimate the clearance time (CT), time taken from the initiation of plasmid clearance until the elimination of all copies, to be in the order of 60 min for both direct interference and priming from the onset of the production rate decrease.

We first investigated the direct interference response (Fig 1A). Prior to *cas* gene induction, the images showed high YFP fluorescence in all cells, confirming the presence of the target plasmid (Fig 2A) which decreased upon induction, indicating CRISPR‐Cas‐mediated degradation of the target DNA (Fig 2A, Expanded view Movie EV1). When the plasmid did not contain a target sequence (pControl), YFP levels did not decrease for over 35 h (Fig EV1A), indicating targeting by CRISPR‐Cas is required for plasmid loss in this set‐up.

The mean YFP fluorescence per cell unit area (which estimates the YFP concentration) showed the decrease started after about 1 h of induction and then exhibited a smooth monotonic decline without substantial cell‐to‐cell variability (Fig 2B). Note that traces end upon the cells exiting the observation chamber. CRISPR‐mediated degradation of the target was thus efficient and synchronous, and in the case of a 5‐copy plasmid could overcome the plasmid replication and copy number control. Hence, we surmised that the YFP fluorescence may decrease exponentially, as the YFP proteins are diluted exponentially due to volume growth upon clearance of the last plasmid. Indeed, we found the fluorescence decrease to be exponential (Fig EV1B).

Direct interference variability between cells also appeared limited (Fig 2B). To address it more directly, we quantified the moment all plasmids are cleared by determining the YFP production rate as the change in total cellular fluorescence per unit of time (Levine *et al*, 2012). The production rate scales with the number of target DNA copies and shows the expression timing more precisely by suppressing slow dilution effects. Indeed, the YFP production rate decreased rapidly and reached zero (the background level of cells not expressing YFP) when the mean fluorescence was still close to its maximum (Fig 2C). This moment was identified as the plasmid loss time (PLT) (Fig 2C). PLT was narrowly distributed between about 1 and 2.5 h (Fig 2D, CV^2^ = 0.055). Hence, in all cells the target was cleared. The clearance was rapid, taking between 1 and 3 generations, and sometimes occurred in the same generation in which the CRISPR‐Cas response was initiated by induction (Appendix Fig S1).

### Primed adaptation is highly variable

Next, we studied plasmid clearance after adaptation from a target with a mutated PAM (Fig 1C). Most notable in these priming experiments was the heterogeneity between lineages, with the clearance process ranging from 2 to 30 cellular generations (Appendix Fig S1). Upon induction, some lineages showed a decreasing trend in fluorescence as early as 4 h (Fig 2E and F, Expanded view Movie EV2), while others remained fluorescent after 35 h (Fig 2F). The PLTs were indeed broadly distributed and displayed a long tail towards large values (Fig 2G, CV^2^ = 0.458). Of note, we did not observe plasmid clearance in the same generation in which the CRISPR‐Cas system was induced (Appendix Fig S1).

The shapes of the YFP declines were exponential, similar to the direct interference data (Figs 2B and F, and EV1B). When aligned at the PLT, the average profile of all production rate traces for direct interference and priming show a similar trend both right before and after plasmid loss is detected (Fig EV1B). In both cases, the onset of the decrease is about 60 min before PLT, thus enabling us to estimate duration of the target clearance process, from here on referred to as CT (clearance time). In priming, clearance therefore contributes much less to PLT variability than the preceding processes (Fig 2G). These observations suggest that new spacers preceded by a consensus PAM are indeed acquired and that the CRISPR adaptation phase is responsible for the observed temporal variability (Fig 2G).

Spacer acquisition in the population was indeed confirmed by PCR of the CRISPR array in cells collected from the microfluidic device (Appendix Fig S2). Spacer acquisition was not observed with the Δ*cas*1,2 strain, consistent with Cas1 and Cas2 being required for acquisition (Yosef *et al*, 2012). In the absence of Cas1 and Cas2, however, low‐frequency plasmid loss was observed in 1.4% of the lineages over a 35‐h period (Fig EV1C). Hence, complete clearance is possible with a mutated PAM, even if highly inefficient.

### Genealogical relations impact the CRISPR‐Cas response

**Figure 3:**
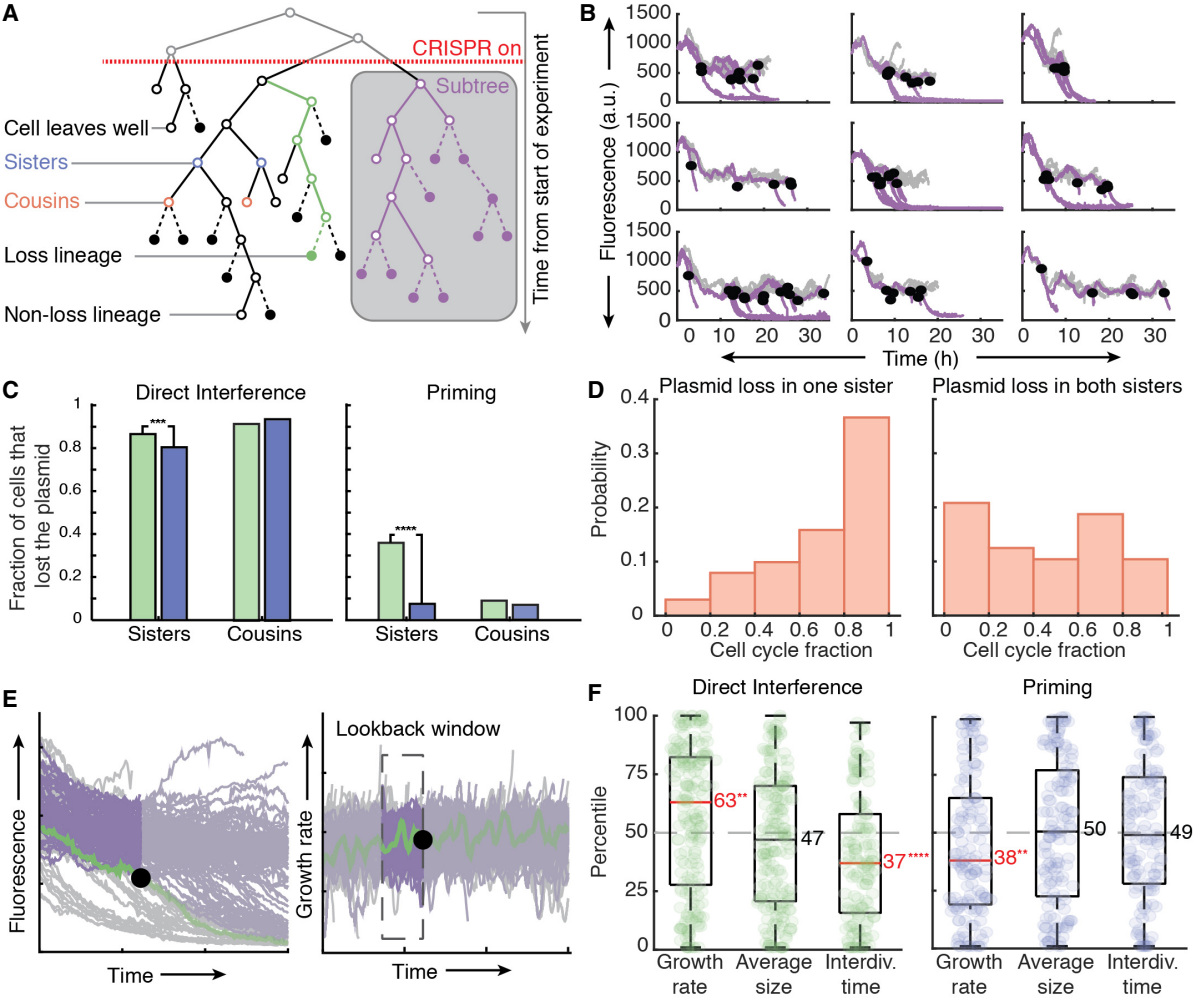
Growth rate and interdivision times have an influence on direct interference and priming Schematic of key analysis structure and terminology. Closed circles indicate cells which clear the plasmid before division, open circles indicate cells which have not yet lost the plasmid.A comparison of 9 subtrees constructed from induction. The subtrees consist of reconstructed fluorescence lineage traces (grey). Loss lineages are indicated in purple with the PLTs indicated by closed black dots.The observed fraction of loss in cells (green) during direct interference (left) or priming (right) related as either sisters (DI: *n* = 171, P: *n* = 98 loss events) or cousins (DI: *n* = 130, P: *n* = 138 loss events) is plotted against the fraction of expected loss events (blue) in related cells when the events are randomised in the same time window. The significance of these values was computed using a Poisson binomial test.The cell cycle was divided into 5 equal fractions and plasmid loss times are plotted in the corresponding fraction where one sister alone cleared the plasmid (left, *n* = 101) or both sisters cleared the plasmid (right, *n* = 24).Schematic explaining the rank‐based analysis approach. For each detected plasmid loss event (left, black circle) the cell feature, that is, the growth rate for the loss‐lineage of interest (right, green) is averaged over a lookback window (right, dashed rectangle) and then ranked amongst all averages of non‐loss lineages in the same window (purple, right).Boxplots of percentile rankings for growth rate, average size and interdivision time over a lookback window of 30 min for all loss lineages that cleared the target. Left, for clearance of a consensus target (green, *n* = 250 biological replicates) the lookback window started 30 min before plasmid loss and ended at the PLT. Right, for clearance of a mutated target (blue, *n* = 149 biological replicates), the lookback window started 90 min before plasmid loss and ended 60 min before loss. The median percentile ranking of loss lineages is indicated by a line and value, categories in which this value was significantly different from a ranking in the 50^th^ percentile as computed by a 2‐sided binomial test are indicated in red followed by asterisks. We follow the standard assumptions of a binomial test, including dichotomy of the items and a sample size significantly smaller than the population size. The bottom and top edges of the box indicate the 25^th^ and 75^th^ percentiles, respectively. The whiskers extend to the most extreme data points not considered outliers. Data information: (C, F) ****P < 0.0001, ***P < 0.001, **P < 0.01.

To study the role of genealogy in the CRISPR‐Cas response, we took a more in‐depth look at the lineage history before plasmid loss (Fig 3A). For primed adaptation, some subtrees showed all plasmid loss events occurring close together; however, most subtrees showed a wide PLT variability (Fig 3B, black dots), in line with lineages responding independently. However, statistical analysis showed that sisters cleared their plasmids within the same cell cycle more frequently than expected at random and more strongly so for priming than for direct interference (Fig 3C). Hence, inheritance plays a role in the CRISPR‐Cas response (Fig 3C).

These data led us to hypothesise that in priming, plasmid loss times in sisters correlate due to spacer acquisition occurring in the mother, after which plasmid degradation (primed interference) continues into the daughters. If true, the detection of plasmid loss in each daughter will likely be close in timing, with the moment in the cell cycle for both daughters determined by when spacer acquisition occurred within the mother’s cell cycle. This would result in a random distribution of loss times throughout the cell cycle for each pair of daughters in the experiment. Conversely, when loss in sisters was not correlated (i.e. only one sister cleared the plasmid), we believe both acquisition and clearance managed to occur in the same cell cycle. In this case, we would expect clearance to occur at the end of the mother’s cell cycle. We base this on our earlier finding that on average ~60 min (CT) is required for the interference process (Fig EV1D), indicating adaptation must occur at the beginning of the cell cycle and be directly followed by swift interference. To test this hypothesis, we divided the cell cycle into five equal fractions and recorded each loss event in the appropriate fraction. Indeed, loss events in just one sister occurred more frequently towards the end of the cell cycle (Fig 3D). In contrast, the moments of plasmid loss were more randomly distributed in the case where both daughters lost the plasmid (Fig 3D). Altogether, this indicated that loss likely takes place more frequently in sisters than cousins (Fig 3C) because adaptation occurred in the mother.

### The growth rate has opposing effects on adaptation and interference

To study whether stochastic variations in cell cycle parameters affect the CRISPR‐Cas response, we developed a ranking analysis to rank each “loss‐lineage” that successfully cleared the plasmids relative to the “non‐loss lineages” that had not cleared the plasmids at that same moment in time (Fig 3E). As cellular features such as the growth rate might not be in a steady state due to changes in the environment, comparing loss lineages that cleared the plasmids at different times over the course of the 36‐h experiment could result in the detection of a trend in growth not related to the CRISPR‐Cas response. The ranking was based on growth rate averaged over a 30 min “lookback window” (Fig 3E), determined using autocorrelation times which are a measure of the rate of change of a time series. The autocorrelation coefficient of the growth rate is no longer significant beyond 30 min, thus indicating measurements more than 30 min apart are unlikely to be correlated (Appendix Fig S3). In direct interference, the “loss lineages” exhibited a higher median growth rate than “non‐loss lineages”, with their growth rate ranking in the 63^rd^ percentile (*P* = 0.01) (Fig 3F). These lineages also showed shorter interdivision times (*P* = 0.0001), but not a difference in cell size (Fig 3F). These results were robust over a range of lookback window sizes (see Appendix Fig S4). We stress that growth is likely only one of the many factors affecting the CRISPR‐Cas response, which is also reflected by the broad ranking distributions (Fig 3F). Overall, the analysis indicated that faster growth in coordination with more frequent cell division has a positive effect on the rate of clearance of a consensus target.

Primed adaptation showed a different picture. To probe the effects on spacer acquisition, which occur about 60 min before plasmid loss, we used a lookback window between 90 and 60 min before the PLT. While cell size and interdivision time did not show an effect (no significant deviation from the 50^th^ percentile) the growth rate did, with loss lineages growing more slowly compared with non‐loss lineages (38^th^ percentile, *P* = 0.01) (Fig 3F). This was robust to changes in the lookback window (Appendix Fig S5). Altogether, these findings indicated that, on average, slower‐growing cells achieved faster plasmid clearance through priming.

### Cascade concentrations impact the CRISPR‐Cas response

**Figure 4:**
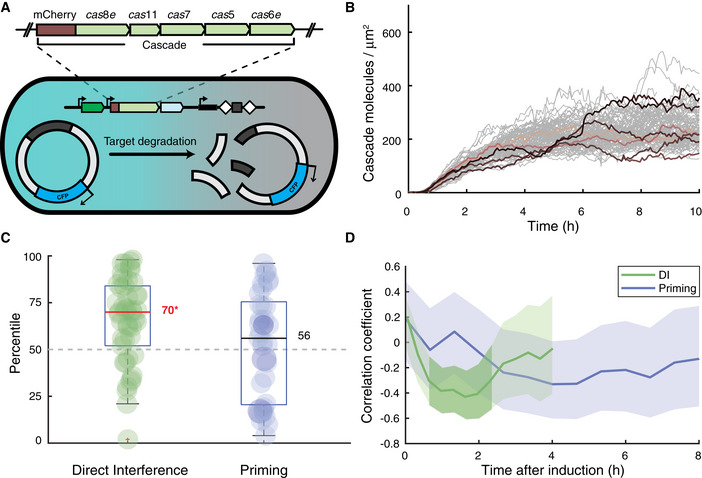
Cellular Cascade concentrations influence the direct interference response Schematic of the experimental set‐up adapted to allow visualisation of target presence (CFP) and Cascade levels (mCherry) simultaneously. The expansion indicates the mCherry fluorescent tag was fused to the N terminus of the *cas8e* subunit.Cascade concentration of single‐cell lineages over time from induction. The surface area of *E. coli* cells in our experiments is approximately 2 µm^2^, resulting in an estimate of approximately 500 molecules of Cascade per cell at steady state.Cascade concentrations were averaged over a 60‐min lookback window from the plasmid loss event for all loss lineages during direct interference (green, *n* = 46 biological replicates) or priming (blue, *n* = 32 biological replicates). The Cascade concentration of the loss lineages was ranked as percentile amongst the non‐loss lineages and plotted here. The median percentile ranking of loss lineages is indicated by a line and value, categories in which this value was significantly different from a ranking in the 50^th^ percentile as computed by a 2‐sided binomial test (*P < 0.05) are indicated in red followed by an asterisk. We follow the standard assumptions of a binomial test, including dichotomy of the items and a sample size significantly smaller than the population size. The bottom and top edges of the box indicate the 25^th^ and 75^th^ percentiles, respectively. The whiskers extend to the most extreme data points not considered outliers.The Pearson correlation coefficient of plasmid loss time versus total cumulative Cascade concentration at that moment is plotted every 5 min (DI) or 10 min (Priming) starting from induction of the CRISPR‐Cas system. The plotted line for both a target with a consensus PAM (green) and target with a mutant PAM (blue) are enveloped by a 95% confidence interval. Darker shading indicates where the correlation coefficient is significantly different from zero (Student’s *t*‐test, *P* < 0.05).

**Figure EV2:**
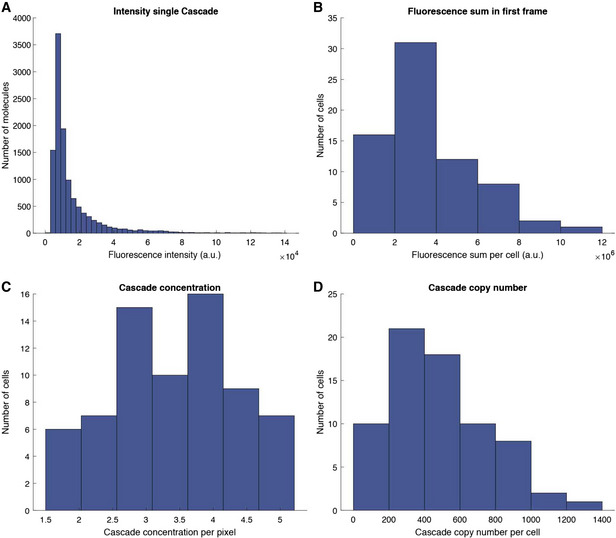
Cascade copy number determination The fluorescence sum (RFP) of each cell in the first frame was determined.The RFP molecules were then bleached until it was possible to determine the fluorescence intensity of a single molecule (representing a single Cascade).The Cascade copy number per cell was then determined by dividing the average fluorescence sum by the average intensity of a single Cascade molecule.The Cascade concentration per pixel was determined by dividing the fluorescence sum by the area of the cell in pixels.

Apart from physiological determinants like growth, Cascade expression levels may influence the speed of CRISPR‐Cas defence, for instance via growth rate fluctuations or the random partitioning of molecules at division (Schwabe & Bruggeman, 2014). To investigate this, we fused mCherry (RFP) to the N terminus of the Cas8e subunit of Cascade (Vink *et al*, 2020) (Fig 4A). Using single‐particle fluorescence calibration, we estimated that the cells contain on average about 200 Cascade molecules/µm^2^ (Figs 4B and EV2). Hence, we quantified the (stochastic) variations in Cascade abundance within single‐cell lineages upon induction (Fig 4B).

**Figure EV3:**
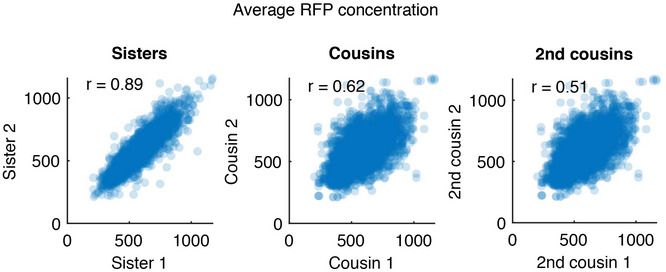
Correlation of RFP levels between cells related as sisters, cousins and second cousins The levels of RFP (Cascade) are strongly correlated between sisters, cousins and second cousins. The correlation coefficient r decreases as the cells become less closely related.

Cascade levels fluctuate on a longer timescale than the cell cycle (200 min, Appendix Fig S6) and are strongly correlated between sisters and cousins (*R* = 0.89 and 0.62, respectively, Fig EV3), indicating that Cascade levels are stable over several generations. We reasoned that lineages with high Cascade concentrations may target and clear the plasmids faster. Hence, we performed time‐lapse experiments and used the ranking approach, now ranking lineages based on the average Cascade in a window of 60 min prior to plasmid loss. For direct interference, loss lineages exhibited significantly higher Cascade levels than non‐loss lineages and ranked in the 70^th^ percentile (*P* = 0.03, Fig 4C). Conversely, no differences in Cascade levels were observed between loss and non‐loss lineages for priming, with the former ranking in the 56^th^ percentile (*P* = 0.32, Fig 4C).

In priming, however, the target search by Cascade occurs over a longer period of time prior to achieving plasmid loss (Fig 2F), likely rendering the ranking approach less suitable due to a limited lookback window. Hence, to test this notion, we investigated the correlation between Cascade search hours and the PLT for each lineage at time points onward from induction. Cascade search hours are defined as the sum of hours spent by all Cascades molecules in the cell searching for the target and are determined from the cumulative RFP, that is, the area under the RFP concentration curve of each lineage from induction until a point of interest (Appendix Fig S7). One may expect that a single‐cell lineage which has a high number of Cascades for a short period of time close to induction could undergo adaptation earlier than a cell which has a lower number of Cascades over a longer period of time, or vice versa (Appendix Fig S7). To this end, we carried out this analysis to determine whether spacer acquisition may be governed by a requirement for a number of Cascade search hours rather than a peak in copy number in the cell.

At 0–2‐h post‐induction, PLT and Cascade search hours indeed correlated negatively for direct interference but not for priming, indicating cells with a higher sum of Cascade search hours lost the plasmid earlier (Fig 4D). This result is in line with our earlier findings (Fig 4C) and supports that stochastic variations in Cascade expression levels affect direct interference. For the priming process, the impact of Cascade levels appeared weaker, and no significant correlation was found between PLT and Cascade search hours prior to the loss (Fig 4D). This may suggest that neither the total search hours of Cascade nor the instantaneous expression levels play a detectable role in the determination of when plasmid loss occurs during priming. We hypothesise this could be due to the underlying processes being less synchronised in time in comparison with direct interference and hence masked by other stochastic variations in our set‐up.

### Low Cascade‐target binding affinity can generate CRISPR‐Cas response variability

**Figure EV4:**
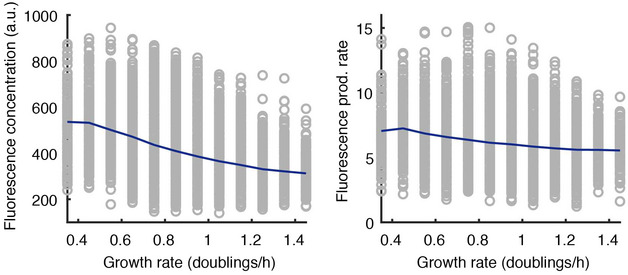
Schematic representation of the agent‐based simulation framework In this framework, we simulate a bacterial population, including exponential cell growth and stochastic division dynamics. At the level of individual cells, we simulate the stochastic reaction kinetics of all steps of the CRISPR defence process. The details of the simulation procedure are described in the Materials and Methods.

**Figure 5:**
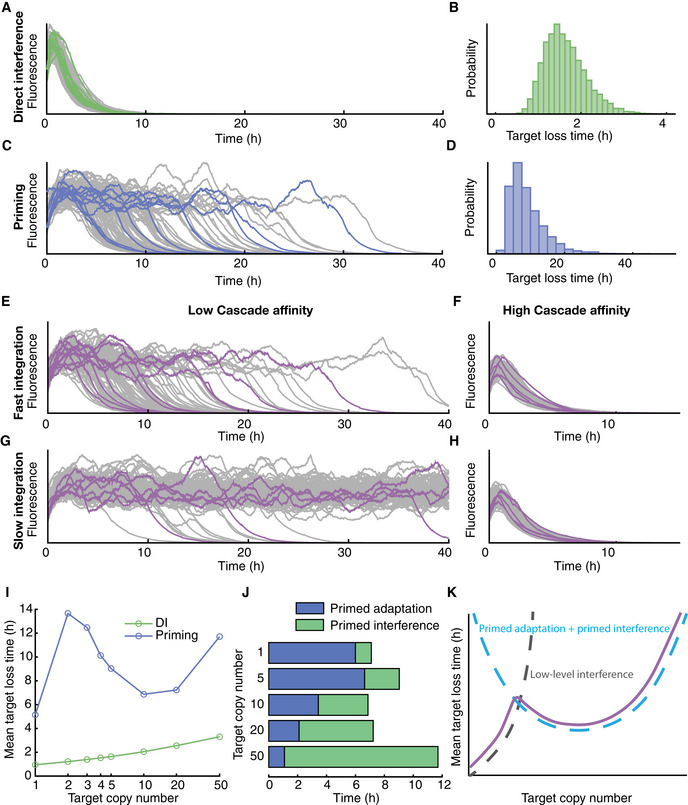
Results from the stochastic agent‐based model of CRISPR adaptation and interference A–DExample trajectories showing fluorescence concentration produced by plasmid containing cells simulated with the agent‐based model for the direct interference (A) and priming condition (C) and corresponding target loss distribution (B,D, respectively).E–HExample trajectories from 4 different parameter combinations. High Cascade affinity (F,H) corresponds to an increase in target binding by a factor of 100 as compared to low Cascade affinity (E,G), slow integration (G,H) represents a 100‐fold reduction in the spacer integration rate as compared to fast integration (E,F).IMean target loss time of the population as a function of the average target copy number per cell for direct interference (green) and priming (blue).JBreakdown of average time spent on primed adaptation (blue) and primed interference (green) for cells that clear targets through priming, for target copy numbers in the range 1‐50.KSchematic of alternative target loss pathways. At low copy numbers, targets can be completely cleared through low‐level interference, which becomes increasingly rare as copy numbers increase. The priming process shows a u‐shaped relationship with the target copy number, as a result of adaptation becoming faster as target copy numbers increase, and time required for interference increasing with target copy number.

To gain insight into the variability and dynamics of the CRISPR‐Cas defence, we developed an agent‐based simulation framework. Adaptive immunity in bacterial populations has been modelled previously (Iranzo *et al*, 2013; Bradde *et al*, 2017; Martynov *et al*, 2017), but to our knowledge none describe variability or single‐cell behaviour. Briefly, we simulated 100 cells, their growth and division, plasmid maintenance, stochastic protein production and partitioning at division, spacer acquisition and target DNA degradation (Fig EV4, see Materials and Methods for details). We found that with these minimal model ingredients and by only changing the Cascade‐target binding affinity due to the PAM mutation, the model reproduced both the low variability of direct interference (Figs 5A and B, and 2B and D) and the high variability of priming (Figs 5C and D, and 2F and G) from the experimental conditions. Specifically, by fitting the model to all available experimental data (Figs 2 and EV1C; Appendix Fig S8), we found a Cascade‐target binding affinity reduction in two orders of magnitude for the PAM mutation, which is consistent with previous work (Jung *et al*, 2017; Cooper *et al*, 2018) (Appendix Table S5).

The priming process can be conceptually understood as a two‐step process, adaptation followed by interference, where the low probability of the first step creates the broadness of the PLT distribution (Materials and Methods). We hypothesised that variation of the primed adaptation response could originate from the low‐affinity target search of Cascade, or the spacer integration. In the agent‐based model, we varied the rates of these two processes by a factor of 100, while keeping the Cas3‐mediated target destruction constant, and found that slow spacer integration alone was not enough to explain the observed variability (Fig 5H). Conversely, reducing the Cascade‐target binding affinity within the model is both necessary and sufficient to reproduce the observations (Fig 5E–H) and is required to generate pre‐spacers.

### Competition between adaptation and low‐level interference

In priming, low Cascade‐target affinity and resulting sporadic target degradation can yield a low‐level interference prior to adaptation, which in turn provides a continuous source of target DNA fragments that can act as pre‐spacers (Künne *et al*, 2016). Hence, we wondered whether target abundance affects this process. For direct interference, as expected, we found that the plasmid loss time (PLT) increased monotonically in simulated trajectories as the average number of targets varies from 1 to 50 (Fig 5I, see Appendix Fig S9 for full range of distributions). Simulations of priming did not show such a monotonic trend: the PLT first went up, then down and finally up again (Fig 5I, Appendix Fig S10). This behaviour could be explained by splitting priming into the adaptation and interference processes (Fig 5J): while primed interference logically only speeds up with fewer targets, primed adaptation initially slows down with fewer targets because of the resulting fewer pre‐spacers, but then speeds up for the lowest number of targets, because low‐level interference is now sufficiently efficient, in combination with unequal partitioning upon division (Appendix Fig S11). Indeed, our experiments also showed such clearance of a 5‐copy target by low‐level interference without spacer acquisition (Appendix Fig S1). This alternative pathway competes with priming when there are few targets (Fig 5K) and might explain the trend in Fig 5J showing faster loss at 1 target as compared to 5 targets. These findings suggest that target abundance affects the balance between primed adaptation and primed interference, resulting in a non‐monotonous trend for the target clearance probability.

### Cascade expression stochasticity can accelerate CRISPR adaptation

**Figure 6:**
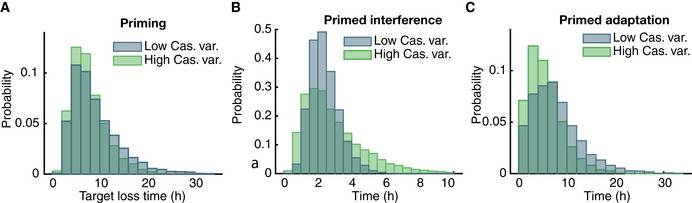
Distribution of primed adaptation and primed interference time for high and low variability in Cascade concentration ATarget loss time distribution for two different levels of Cascade concentration variability for priming. At low variability (blue) Cascade proteins are produced in frequent, small bursts, whereas at high variability (green) proteins are synthesised more sporadically in large bursts (100‐fold increase), keeping average Cascade concentration constant.B, CThe variability of primed interference times (B) for high Cascade variability (green) increases as compared to low Cascade variability (blue), whereas the variability of primed adaptation times (C) decreases with higher Cascade variability.

Our experiments showed that CRISPR‐Cas defence is affected by Cascade expression (Fig 4C and D) which is stochastic in nature (Fig 4B). However, due to the inducible promoter set‐up in our experiments, variability in Cascade levels may be lower than in a natural setting. To investigate the possible implications, we changed the level of gene expression variability for Cascade to exhibit 100‐fold stronger expression bursts while maintaining average Cascade concentrations (see Materials and Methods for details). For direct interference simulations, this increased variability resulted in a higher mean PLT: while some cells could clear all targets earlier, many cells required more time to clear all targets as compared to lower‐variability Cascade expression (Appendix Fig S12). Surprisingly, for priming the mean PLT became lower when the Cascade variability increased (Fig 6A). The primed interference phase showed a trend similar to direct interference: a broadening of the PLT distribution yielding a slow‐down on average (Fig 6B). However, the entire distribution shifted to lower values for primed adaptation (Fig 6C), yielding an overall speed‐up. For mutated PAMs, pre‐spacer production critically depends on high Cascade levels, even if transient, as the cumulative probability of a pre‐spacer integration event depends on the Cas concentration in a highly non‐linear fashion. In Appendix Fig S14, we show how this non‐linear dependence results in an increased probability of adaptation for cells with high Cascade variability as compared to cells with low variability, while having equal average Cascade concentrations.

## Discussion

In this study, we have investigated a previously unexplored question: what are the dynamics and variability of the CRISPR adaptation and interference responses in individual cells? Our time‐lapse microscopy approach allowed real‐time monitoring of invader presence, cell traits and inheritance in single‐cell lineages. We found that direct interference, despite its dependence on various stochastic processes and poorly understood competition between replication of invading nucleic acids and degradation by CRISPR‐Cas systems, is notably deterministic and efficient, with invader DNA clearance achieved in all cells within 1–3 generations. Conversely, the priming CRISPR‐Cas response was highly variable, ranging from 2 to 30 generations before clearance. Our data show that direct interference and primed interference can in fact occur on comparable time scales and identify the adaptation phase of priming as the origin of the variation. Further, our direct observation of the CRISPR‐Cas action and modelling approach revealed several factors that impact CRISPR‐Cas response variability. The interaction between Cascade and the target DNA, which is characterised by a low affinity owing to the PAM mutation, represents a key source of heterogeneity in the adaptation process of the priming response, rather than the complex spacer integration process.

For direct interference, we found our observed degradation time of ~90 min on average for the consensus target plasmids to be on a comparable time scale to previous work (Guan *et al*, 2017) taking into account the differences in experimental set‐up including but not limited to: the number targeting spacers, copy number of targets and differences in Cas protein induction. While in agreement with the work of Guan *et al*, 2017, we found that cell size was not an influential factor in the speed of target degradation; we additionally found that cells that cleared the target DNA earlier, grew and divided faster than the population mean. This may be explained by the fact that faster growth is known to reduce plasmid copy numbers (Lin‐Chao & Bremer, 1986; Klumpp, 2011).

**Figure EV5:**
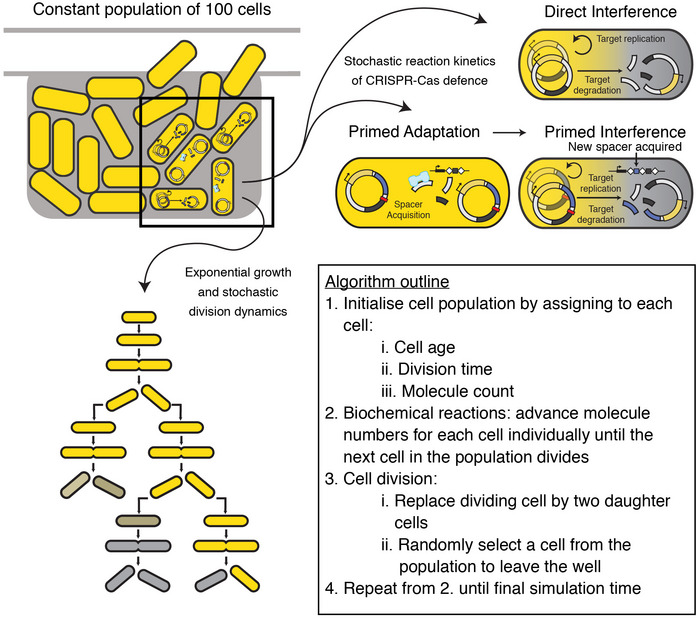
Slower‐growing cells have higher Cascade concentrations Cascade concentration (left) and Cascade production rate (right) show an inverse relationship with cellular growth rate (grey circles), revealing slower‐growing cell on average (navy line) have a higher concentration of Cascade.

For priming, the reverse was found. Cells that adapted and cleared the target DNA earlier grew more slowly than the population mean. From this, we conjectured that slower growth may lead to more spacer acquisition events or that spacer acquisition may cause slower growth of cells. We speculate that the first explanation is most likely explanation may play a role due to copy number maintenance mechanisms, which result in higher concentrations of target plasmids in slow‐growing cells (Ingmer *et al*, 2001). This hypothesis was further supported by the model which showed that adaptation occurs more efficiently in the presence of a higher number of targets (Fig 5J). While in our set‐up an effect of Cascade concentration on priming could not be detected, we note that slower‐growing cells had higher Cascade abundance (Fig EV5), suggesting that Cascade levels may play a role in combination with other factors enhanced by slow growth. Slow growth may simply provide more time to the cell to locate the target and facilitate spacer insertion before interruption by cell division (Høyland‐Kroghsbo *et al*, 2018). In line with this idea, recently published studies on primed CRISPR adaptation in I‐F systems in *Pseudomonas aeruginosa* have found a causal link between reduced bacterial growth rates and increased spacer acquisition (Dimitriu *et al*, 2022). This finding was attributed to slower phage development induced by bacteriostatic antibiotics, allowing more time for cells to acquire spacers especially in the late‐exponential and early‐stationary phases, when cells are presumably growing slower (Amlinger *et al*, 2017), and in slow‐growing populations when compared directly to faster‐growing populations (Høyland‐Kroghsbo *et al*, 2018). Our findings together with these studies indicate that slow growth caused by any environmental change or cellular stress may in fact be beneficial to a cell trying to undergo adaptation.

Our findings suggest that target copy number influences the efficiency of spacer acquisition, which has implications for phage invasion. It implies that one genome copy of a single virulent phage with an escape PAM may not lead to efficient CRISPR adaptation. However, upon replication of the phage genome, it may become abundant enough, though at this point in time it is likely that primed interference with a new spacer cannot successfully eliminate a virulent phage before cell lysis (Hynes *et al*, 2014; Davison, 2015; Severinov *et al*, 2016). Despite this, it has been shown bioinformatically that priming by type I systems is widespread in nature (Nicholson *et al*, 2019), especially against temperate phages (Nobrega *et al*, 2020). Such events could occur due to low‐level interference, in which a cell is able to simultaneously clear the invader while present as a single copy and acquire a spacer from the fragments produced. This would result in the immunisation of a single cell in the population, ultimately leading to a subpopulation of resistant cells that could limit further propagation of the same phage. Such a phenomenon may be more likely to occur when a defective phage infects the cell (Hynes *et al*, 2014).

The variation existing between single cells in a population is remarkable. Stochasticity or noise in gene expression and cellular components has been demonstrated to play crucial roles in many cellular processes (Balaban *et al*, 2004; Moormeier *et al*, 2014; Uphoff *et al*, 2016). We anticipate that the dynamics and heterogeneity of the CRISPR‐Cas system, as studied here, play an important function in strategies that bacteria exploit and evolve in their continuous competition with phages, as well as with other species. For instance, CRISPR‐Cas could contribute to bet‐hedging strategies (Acar *et al*, 2008), in which subpopulations develop to combat changes in the environment, such as phage exposure. A distinct subpopulation in which Cascade is highly expressed could allow faster elimination of an invading phage and subsequent re‐population. This may in turn increase the fitness of the population, by reducing the overall burden of CRISPR‐Cas expression and risk of autoimmunity (Westra *et al*, 2015; Staals *et al*, 2016) and hence outcompete other bacterial strains. While such a strategy may not guarantee single‐cell survival, it is at large beneficial for the population as a whole. Indeed, previous studies have shown CRISPR‐Cas immunity in single cells acts to limit phage propagation throughout the population in an abortive infection‐like manner (Strotskaya *et al*, 2017; Watson *et al*, 2019; preprint: Lopatina *et al*, 2020). On the contrary, the survival of only a subpopulation of cells may result in population bottlenecking and an overall loss of diversity (Moxon & Kussell, 2017). This may be disadvantageous in terms of spacer diversity, where it has been shown that populations containing a range of spacers are better able to combat and even facilitate the extinction of new invaders (van Houte *et al*, 2016; Martynov *et al*, 2017). Further, we cannot discount that a more susceptible subpopulation of cells may lead to higher overall phage titres and a larger overall threat to the population.

While a number of studies have thoroughly investigated CRISPR‐Cas systems through population and single molecule‐based experiments (Barrangou *et al*, 2007; Marraffini & Sontheimer, 2008; Richter *et al*, 2014; Patterson *et al*, 2016; Staals *et al*, 2016; Amlinger *et al*, 2017; Strotskaya *et al*, 2017; Høyland‐Kroghsbo *et al*, 2018; Musharova *et al*, 2019; Watson *et al*, 2019; Xue & Sashital, 2019), these findings do not provide insight into the cell‐to‐cell variability. Our work, along with others (Guan *et al*, 2017; preprint: Mamontov *et al*, 2021), has begun to bridge this gap demonstrating how important the dynamics of CRISPR‐Cas systems are to their functioning and the outcome of populations facing a threat. Further investigation into different CRISPR‐Cas types and classes, fluctuating environments (Nguyen *et al*, 2020), and conditions supporting the formation of subpopulations (Spormann, 2008) will enhance the understanding of CRISPR‐Cas dynamics on both the molecular and population scale.

## Materials and Methods

### Cloning

Plasmid pTU166 targeted by KD615 and KD635 was created by amplifying the streptomycin resistance cassette from pCDFDuet‐1 with primers BN831 and BN832 to add a 5′CTT‐PS8 tail. The backbone of pVenus was amplified using primers BN833 and BN834, and both products were restricted with KpnI and HindIII enzymes. Overnight ligation at 16°C and transformation into DH5ɑ resulted in colonies selected to contain the plasmid. Plasmids pTU190 and pTU193 were created by PCR amplification of pTU166 using primer BN911 in combination with BN912 or BN891, respectively. Products were restricted with SalI, ligated and transformed into DH5a. Target plasmids pTU389 and pTU390 were PCR amplified from plasmid pTU265 a derivative of pVenus containing CFP using primers BN2278 in combination with BN2275 or BN2276, respectively. Products were restricted with NcoI, ligated and transformed into DH5a. All plasmids were confirmed by Sanger sequencing (Macrogen). All plasmids used are listed in Appendix Table S1. Primers used are listed in Appendix Table S2.

### Creation of strains KD615mCherry‐Cas8e and KD635mCherry‐Cas8e

Strains were created using lambda red homologous recombination (Datsenko & Wanner, 2000). Plasmid pSC020, containing both Lambda red and the Cre‐recombinase, was transformed by electroporation into strains KD615 and KD635. Strains were recovered at 30°C for 1.5 h and plated on media containing 100 μg/ml ampicillin. Transformants were then grown overnight in liquid medium at 30°C, with selection, and made competent the following day by inoculating 50 ml with 500 μl of overnight culture. Once the cells reached, an OD_600_ of 0.2 a final concentration of 0.2% L‐Arabinose (Sigma‐Aldrich) was added and cells were grown for another 1.5 h and subsequently washed with pre‐cooled 10% glycerol.

The mCherry‐*cas*8*e* G‐block (IDT) (Appendix Table S3) based on the design used in (Vink *et al*, 2020) was resuspended with ddH_2_O to a concentration of 50 ng/µl and transformed into the competent cells by mixing 2 μl DNA with 50 μl of cells and recovering at 30°C for 1.5 h. After recovery, cells were plated undiluted with selection for kanamycin and ampicillin. PCR‐verified colonies were then grown in liquid culture with 1 mM IPTG at 37°C to promote the loss of the kanamycin resistance cassette and pSC020. Individual colonies were screened for plasmid loss by patching each colony onto three plates containing no antibiotics, only kanamycin and only ampicillin. Colonies exhibiting no resistance were then PCR screened with primers (Appendix Table S2) BN2204 and BN2205 for the presence of the mCherry‐Cascade fusion. Strains were confirmed by Sanger sequencing (Macrogen).

### Growth conditions

All strain and plasmid combinations (Appendix Table S1) used were grown at 37°C, shaking at 180 rpm, prior to microscopy. To avoid autofluorescence under the microscope, a minimal M9 media was used containing the following supplements; 2% glycerol (Sigma‐Aldrich), 1X EZ Supplements (M2104 Teknova), 20 μg/ml uracil (Sigma‐Aldrich), 1 mM MgSO_4_ (Sigma‐Aldrich) and 0.1 mM CaCl_2_ (Sigma‐Aldrich), from here on called M9 media.

### Microfluidic device

The device used was developed by D.J. Kiviet in the Ackermann laboratory and has been previously used in the Tans laboratory (Wehrens *et al*, 2018). The device contains a main flow channel 23.5 µm high and 200 µm wide that splits into two 100 µm wide flow channels of the same height. Perpendicular to these flow channels are wells with a height of 0.75 µm, widths of 1 × 80 µm, 1 × 60 µm, 2 × 40 µm, 3 × 20 µm, 3 × 10 µm, 3 × 5 µm and depths of 60, 30, 50 and 40 µm. These well sizes are repeated five times and are the location where the growth of microcolonies occurs during an experiment. The PDMS devices were made by casting them into an epoxy mould, a gift from D.J. Kiviet and the Ackermann laboratory.

The PDMS device was produced by mixing polymer and curing agent (Sylgard 184 elastomer, Dow Corning) in ratio of 1 ml of curing agent to 7.7 g of polymer. This mixture was poured into the epoxy mould, and air bubbles were subsequently removed by use of a desiccator for 30 min followed by baking at 80°C for 1 h. After baking, the device can be carefully removed from the mould with the aid of a scalpel and holes were punched for liquid in‐ and outlets. For use under the microscope, the PDMS chip was covalently bound to a clean glass coverslip. This was done by treating both the PDMS and glass surface with 5–10 sweeps of a portable laboratory corona device (model BD‐20ACV, Electro‐Technic Products). After treatment, the chip was placed carefully onto the glass slide and gently tapped to facilitate full contact between the PDMS and glass surface. Finally, the device was baked for another 1–2 h at 80°C and stored until the experiment was started.

### Loading and filling of microfluidic wells

Cells were initially grown overnight (for ~12 h) at 37°C, 180 rpm in 10 ml M9 media with antibiotic selection (streptomycin 50 µg/ml) for the target plasmid. The following day 500 µl of culture was passaged into fresh M9 medium (with selection for the target plasmid), approximately 3 h before microscope set‐up and grown at 37°C, 180 rpm. After 3 h of growth, the cells were pelleted and resuspended in ~30 µl.

To begin the experiment, 2 µl of 0.01% Tween20 (dH_2_O) solution is slowly pipetted into the selected media lane to allow the removal of air and flow of liquid into the wells perpendicular to the media lane. Following this, 2 µl of concentrated bacterial culture was pipetted slowly into the same lane. Once liquid could be seen exiting at the opposite end of the media lane the syringes containing media (loaded on syringe pumps), the valve controller and the waste collection flasks were attached to the chip by metal connectors and polyethene tubing. Media was pumped into the chip at a flow rate of 0.5 ml/h allowing constant supply of nutrients to the cells. The rate of media flow was also important for the removal of cells from the top of the well, to allow constant division and long‐term tracking of cells located lower within the well.

### Media switches

All experiments were carried out with precise 37°C temperature control and required the use of 2 different media. For the first 12 h of the experiment (including loading of the chip), cells were grown in Media 1; M9 supplemented with both anhydrotetracycline (40 ng/ml) and streptomycin (25 µg/ml) to induce the YFP and select for cells containing the target plasmid, respectively. After 12 h of growth in the chip, the media was switched via the valve controller (Hamilton, MPV valve positioner) to Media 2; M9 supplemented with anhydrotetracycline (40 ng/ml), 0.1% L‐arabinose and 0.1 mM IPTG. This media change allowed removal of the selection for the target plasmid, continued induction of the YFP and induction of the CRISPR‐Cas system after filling of the wells.

### Spacer acquisition detection from microfluidic chip output

Over the course of the experiment, the cells that flow out of the wells and subsequently the chip were collected in a sterile Erlenmeyer flask. The cells were then concentrated by centrifuging for 5 min at 2,000 *g*. The supernatant was removed, and cells were resuspended in 2 ml of M9 media. Colony PCR was performed with 1 μl of culture using primers BN1530 and BN1531 (Appendix Table S2), and the products were run on a 2% agarose gel at 100 V for 30 min alongside the 100–1,000 bp DNA Ladder (SmartLadder‐SF, Eurogentec).

### Imaging and image analysis

For all time‐lapse experiments, phase contrast images were acquired at 1‐min intervals at a maximum of 2 positions. In experiments with a YFP target plasmid, fluorescent images were taken every 2 min, with an exposure time of 500 ms. For experiments with a CFP target plasmid and the mCherry‐Cascade fusion, images were acquired every 4 min with exposure times of 500 and 200 ms, respectively. Images were acquired for the entire experiment including the first 12 h of growth. Cells were imaged with an inverted microscope (Nikon, TE2000), equipped with 100× oil immersion objective (Nikon, Plan Fluor NA 1.3), automated stage (Märzhäuser, SCAN IM 120 3 100), high‐power LED light source with liquid light guide (Sutter, Lambda HPX‐L5), GFP, mCherry, CFP and YFP filter set (Chroma, 41017, 49008, 49001 and 49003), computer‐controlled shutters (Sutter, Lambda 10‐3 with SmartShutter), cooled CMOS camera (Hamamatsu, Orca Flash4.0) and an incubation chamber (Solent) allowing temperature control. In order to obtain images with a pixel size of 0.041 µm, an additional 1.5× lens was used. The microscope was controlled by MetaMorph software. A series of acquired phase contrast images were analysed with a custom MATLAB (MathWorks) programme, originally based on Schnitzcells software (Young *et al*, 2012), adapted to allow for automated segmentation of cells growing in a well (Wehrens *et al*, 2018). Segmentation was inspected and corrected manually where necessary. All segmented cells were then tracked between frames using the pixel overlap between cells allowing the formation of lineage structures (Wehrens *et al*, 2018). Growth rates are determined by fitting an exponential function to recorded cell lengths over multiple frames and thus represent the rate of cell elongation, whereas interdivision time is calculated as the time between subsequent divisions.

### Plasmid loss and clearance time detection using the fluorescent protein production rate

Before screening for plasmid loss, we detect cell death in lineages by applying a moving average filter to the cellular growth rate. If the cellular growth rate reached zero and did not recover again, the remainder of the fluorescence time series after this point was excluded from the analysis. For each lineage, we computed the fluorescence production rate of the plasmid‐encoded fluorophore from a cell’s total fluorescence, cell area, cellular growth rate and the rate of photobleaching of the fluorophore (Levine *et al*, 2012). As there is always some amount of residual fluorescence produced by the cells, we selected an appropriate threshold for plasmid loss detection from the upper values of the distribution of production rates of plasmid‐free cells. To detect plasmid loss in individual lineages, we applied a moving average filter to the fluorescence production rate and detected the first instance of the production rate reaching a value below the threshold. This plasmid loss time (PLT) can be seen as an upper bound estimate, as some processes (transcription, translation and fluorophore maturation) still carry on for some time after the last plasmid has been cleared but could not be measured in our set‐up. The onset of the clearance time (CT), which signifies the start of the destruction of all plasmids through interference and ends at the plasmid loss time (PLT), is difficult to detect in individual lineages due to the naturally occurring fluctuations in the fluorescence production rate. To determine this quantity, we align all plasmid loss lineages at the PLT and compute the average trend. The CT per experimental condition is approximated as the duration from the point where the average production rate starts to decrease until the PLT.

### Sister and cousin statistics

For each lineage that lost the plasmid, we wanted to compare the probability of loss in an unrelated cell and in a related cell. For related cells, we counted the frequency of loss and non‐loss in sister and cousin cells of the loss cell, but only if the sister or cousin divided (contained a complete cell cycle). For unrelated cells, we counted the total number of loss events (*i*) that occurred throughout the cell cycle of the related cell. For each loss event, we counted how many cells (*c_i_*) still contained the plasmid up to that point. The probability of plasmid loss happening in an unrelated cell during the lifecycle of the related cell was subsequently calculated recursively using the following equations:

$$p_{0}=0$$

$$p_{i}=\frac{1-p_{i-1}}{c_{i}}+p_{i-1}$$

where *p_i_* is the probability of loss occurring within an unrelated cell given *i* plasmid loss events occurred within the cell cycle of the related cell and *c_i_* stands for the number of cells still containing the plasmid at the same time as the *i*‐th plasmid loss event.

### Cascade copy number determination

The control strain KD614 mCherry‐Cas8e containing plasmid pTU265 (Appendix Table S1) was prepared and loaded into the microfluidic chip as above. After 12 h, a sterile tube was connected to the waste tubing and output from the chip was collected for 30 min. The media was then switched to induce Cascade. Approximately 5 h after induction when Cascade levels are considered to be stabilised, the output from the chip was again collected for 30 min. To improve counting, cells were subsequently fixed with 2.5% paraformaldehyde solution at 22°C for 45 min (Uphoff *et al*, 2013). Slides were cleaned by sonication in subsequent steps with MilliQ, acetone and KOH (1 M). Next, 1% agarose pads containing the M9 medium were prepared and hardened between two slides within 20 min of measuring to prevent desiccation. The fixed cells were then spun down and resuspended in 5 µl of which 1 µl was pipetted onto a pre‐prepared agarose pad.

The cells were imaged using a TIRF microscope (Olympus IX81; Andor Ixon X3 DU897 EM‐CCD camera) using a high‐power 561 nm laser, which quickly bleached most mCherry molecules within a couple of frames. Intensity of single molecules was measured with Thunderstorm starting from the thirtieth frame (Ovesný *et al*, 2014). The total cell fluorescence was measured by segmenting the cells from the phase contrast image and sum fluorescence counts of all cell pixels (with background subtracted). The copy number was calculated by dividing the total cell fluorescence in the first frame by the average fluorescence intensity of the single molecules. We could then calculate the Cascade concentration ~200 Cascade molecules/µm^2^ by dividing the population average of the mean summed RFP per cell by this copy number, which was applied to the cells in our time‐lapse data.

### Master Equation description of the probability of plasmid loss

In order to test whether the distribution of the target clearance times by direct interference can be reproduced by a simple one‐step process, we consider a model using a compound probability for binding of Cascade to the target and subsequent target removal from the system. In bacteria, the number of targets is subject to maintenance which delays the removal of *M*_0_ targets. For sake of simplicity, we ignore this additional step, which has the advantage that the number of unknown parameters is kept to an absolute minimum. Because direct interference is a fast process, one can assume that target maintenance does not have a strong effect on the clearance time distribution. The Cascade number is not constant, but rather Cascade production is induced at the beginning of the experiment. This simplified model only depends on five parameters: the delay after induction for production of Cascade *τ_c_*, the Cascade production rate *σ*, the turn‐over rate of Cascade *λ*, the number of targets per cell *M* and the probability of a target removal event *p_d_*. The number of targets in individual cells will be in general stochastic; however, due to target maintenance one can assume that this distribution will be quite narrow. For this reason, we set *M*_0_ = 5 (Thompson *et al*, 2018).

The time‐dependent Cascade copy number is modelled as a production–degradation process with a delay *τ_c_* and zero initial amount of Cascade: the bulk mean *µ*(*t*) is given by:

$$\mu \left(t\right)=\frac{\sigma }{\lambda }\theta \left(t-\tau_{c}\right)\left(1-e^{-\lambda (t-\tau_{c})}\right).$$

By fitting this equation to Cascade concentration data for the bulk mean (Fig 4B), we estimate the following: *τ* *_c_* = 34 min, *σ* = 3/min and *λ* = 0.0061/min to obtain an average copy number of almost 500 Cascades per cell at steady state.

The removal of *M*_0_ targets from the system is a First‐Passage‐Time problem. We formulate the simple Master Equation (ME) for the conditional probability *P_M_* (*t*) to find *M* targets in a cell at a given time *t*:

$$\frac{dP_{M}\left(t\right)}{\mathrm{dt}}=\mu \left(t\right)p_{d}\left(M+1\right)P_{M+1}-\mu \left(t\right)p_{d}MP_{M^{\prime }}$$

where *p_d_* is the compound probability that within the time interval Δ*t* a Cascade molecule binds to a target and the target is subsequently removed from the system.

To obtain the First‐Passage‐Time distribution, we need to determine the survival probability *S* to find at least one target, which is simply given by *S* = 1 – *P*_0_. *P*_0_ is obtained by solving the above ME with the initial condition $P_{M}\left(t=0\right)=\delta_{MM_{0}}$:

$$P_{0}\left(t|M_{0}\right)={\left[1-e^{-p_{d}\int_{0}^{t}\mu (t^{\prime })dt^{\prime }}\right]}^{M_{0}}$$

*P*_0_(*t*|*M*_0_) = 0 for *t* < *τ*_*c*_ and because the state *M* = 0 is naturally an adsorbing boundary we readily find $\lim_{t\to \infty }P_{0}\left(t|M_{0}\right)=1$. The First‐Passage‐Time distribution *FPr*(*t*|*M*_0_) for target removal is given by $FP_{r}=-dS/dt=dP_{0}/dt$:

$$FP_{r}\left(t|M_{0}\right)=M_{0}p_{d}\mu \left(t\right){\left[1-e^{\;p_{d}\int_{0}^{t}\mu (t^{\prime })dt^{\prime }}\right]}^{M_{0}-1}$$

Fitting this distribution to the empirical data (Fig 2D) gives rise to *p_d_* = 4.4 × 10^−4^/min. The average target removal time *τ* is given by:

$$\tau =\int_{0}^{\infty }t^{\prime }FP_{r}\left(t^{\prime }|M_{0}\right)dt^{\prime }$$

Using the estimates for *p_d_*, *σ*, *λ*, *τ_c_* and *M*_0_ = 5, we obtain τ≈94 min. The fit of *FP_r_* to the data can be seen in Appendix Fig S13A.

The simplified model yields a decent fit to the direct interference data. What about the target clearance during priming? To investigate whether this can be conceptually understood by a two‐step process, first spacer acquisition subsequently followed by primed interference, we condition *FP_r_* on the time *τ_p_* needed for spacer acquisition:

$$FP_{r}\left(t|M_{0},\tau_{p}\right)=M_{0}p_{d}\theta \left(t-\tau_{p}\right)\mu \left(t\right){\left[1-e^{-p_{d}\int_{\tau_{p}}^{t}\mu \left(t^{\prime }\right)dt^{\prime }}\right]}^{M_{0}-1}$$

The rationale behind this is that a Cascade molecule needs to bind to a target to produce the pre‐spacers necessary for spacer acquisition before primed interference can happen. It follows $FP_{r}\left(t|M_{0},\tau_{p}\right)=0$ for *t* < *τ_p_*. Note that *τ_p_* ≥ *τ_d_*, since in the absence of Cascade the probability of spacer acquisition is negligibly small. The distribution for *τ_p_* is given by the First‐Passage‐Time distribution for the passage $M_{0}\to M_{0}-1:FP_{p}=-dP_{M_{0}}/dt$:

$$FP_{p}\left(\tau_{p}|M_{0}\right)=M_{0}p_{p}\mu \left(\tau_{p}\right)e^{-M_{0}p_{p}\int_{0}^{\tau_{p}}\mu \left(t^{\prime }\right)dt^{\prime }}$$

where *p_p_* is the compound probability that within the time interval Δ*t*, one Cascade binds to a target, pre‐spacers are produced and a spacer is integrated.

The distribution for the target removal times is given by:

$$FP_{\tau }\left(t|M_{0}\right)=\int_{0}^{\infty }FP_{r}\left(t|M_{0}-1,\tau_{p}\right)FP_{p}\left(\tau_{p}|M_{0}\right)d\tau_{p}$$

The integral cannot be done analytically. Fitting *FPr*(*t*|*M*_0_) to the experimentally obtained data for the distribution of target loss times during priming (Fig 2E) yields *p_p_* = 10^−6^/min. The fit of *FP_τ_* to the data can be seen in Appendix Fig S13B.

### An agent‐based model for stochastic biochemical kinetics of cell populations in microfluidic wells

Although a highly simplified description of our system, the results from the ME description show that the Cascade copy number is an important determinant in creating the variability in the PLT distribution in the case of direct interference. For priming, the distribution could be replicated by considering the process as the result of two subsequent steps, of which the spacer acquisition process creates the wide PLT distribution. However, this model of primed adaptation is highly simplified and does not give any mechanistic insight into the process of adaptation and interference in a growing cell population. To better understand how cell‐to‐cell variability and population dynamics affect CRISPR‐Cas defence, we have developed a stochastic, agent‐based simulation framework to analyse the kinetics of spacer acquisition and target loss. An agent‐based approach allows us to keep track of the biochemical composition of individual cells in a growing population, as well as the inheritance of molecules and other cellular features in lineages. In this type of model, each cell is an agent, and there is no interaction between cells. For computational efficiency and to emulate the experimental set‐up, the size of the cell population is kept constant. Results for this type of set‐up, where the population size is constant, are identical as for a population experiencing exponential population growth, as long as the population size is sufficiently large (100–1,000 cells) (Thomas(Voliotis *et al*, 2016).

### Model assumptions

Since the detailed mechanism of primed spacer acquisition in type I‐E CRISPR‐Cas systems is not yet completely known, we start out with a simplified model to see whether this is sufficient to explain our data. Because primed adaptation is much more efficient than naive adaptation (preprint: Stringer *et al*, 2020), we assume that the rate of naive adaptation is negligibly small over the time course of the experiment. The spacer composition of the CRISPR array is not modelled in detail. Rather, we assume that we start out with a crRNA sequence that matches the target, but is flanked by a non‐consensus PAM. The effector complexes containing this spacer can still bind to the target DNA (Semenova *et al*, 2011; Musharova *et al*, 2019), but with a binding affinity that is decreased up to a factor 100–150 as compared to binding with a consensus PAM (Jung *et al*, 2017; Cooper *et al*, 2018 *)*. Once the effector complex is bound to the target, Cas3‐catalysed destruction of the target takes place (Krivoy *et al*, 2018). Thus, the level of interference is associated with the level of effector complex binding (Cooper *et al*, 2018).

Cas3‐mediated destruction of targets is a source of substrates for spacer acquisition machinery, the Cas1‐Cas2 complex, during primed adaptation (Künne *et al*, 2016; Semenova *et al*, 2016). Intermediates of target DNA degradation are transient and quickly degrade after an initial burst. Abundant levels of Cas1 and Cas2 lead to robust spacer acquisition, by allowing Cas1‐Cas2 to capture the transient intermediates of Cas3 action (Semenova *et al*, 2016). Since in our system Cas3, Cas1 and Cas2 are highly expressed, we assume the levels of these proteins are not rate‐limiting within the scope of our model and thus do not explicitly model their abundances. Furthermore, in agreement with previously published work, we assume cells have a target maintenance system that is controlled by logistic dynamics to keep the target concentration at its target level (Severinov *et al*, 2016). In addition, targets and target‐containing configurations are actively partitioned between daughter cells (Meacock & Cohen, 1980; Shao *et al*, 2015) according to a multi‐hypergeometric distribution, with each daughter receiving on average half of the mother cell’s targets. All other proteins are partitioned according to a Binomial distribution, where the ratio of daughter cell sizes determines the probability of each molecule ending up in one of two daughter cells. We model the synthesis of CRISPR proteins as a Poisson process, in which proteins are produced in geometrically distributed bursts to capture the effect of transcriptional bursting (Golding *et al*, 2005). We assume all molecular species are stable on the timescale of the experiment (*i.e*. no degradation), with the exception of the free crRNAs (not loaded in Cascade) and the DNA fragments that are the result of interference, which have a short lifetime.

### Algorithm outline

For the agent‐based model, we have adapted the First‐Division Algorithm by Thomas (Thomas, 2017) to include the *Extrande* extension to the SSA. Furthermore, we keep the population size constant by randomly selecting a cell to be removed from the population in the event of a cell division. The steps to replicate our experimental set‐up are described below.

#### Population initialisation

At time *t* = 0, initialise *N* cells by assigning to each cell an age $t_{i}∼U\left(-\log\left(2\right)/\mu_{p},\log\left(2\right)/\mu_{p}\right)$, a growth rate $\mu_{i}∼Lognormal(\mu_{p},\sigma_{p}^{2})$ and molecule count *x_i_*. Select division size $V_{d,i}∼Lognormal(\mu_{V_{n}}\;,\sigma_{V_{n}}^{2})$ and compute generation time *t_gen,i_* as $\log\left(V_{d,i}/V_{b,i}\right)/\mu_{i}$, where *V_b,i_* is the birth size. This determines the division time of the cell which is defined as $t_{d,i}=t_{i}+t_{gen,i}$.

#### Biochemical reactions

Determine the next dividing cell: $j=\arg\min_{i}\left(t_{d,i}-t_{i}\right)$. Determine Δ*t* from $\min\left(t_{d,j}-t_{j},L\right)$, where *L* is Extrande’s look‐ahead horizon. Advance the molecule numbers of each cell independently from age *t_i_* to *t_i_* + Δ*t* using the Extrande algorithm and advance time from *t* to *t* + Δ*t*.

#### Cell division

When *t* = *t_d,j_*, replace the dividing cell by two newborn daughter cells of zero age. The birth size of both daughters is determined as $V_{b,D_{1}}=Normal(\mu_{V_{R}},\sigma_{V_{R}}^{2})V_{d,j}$ and $V_{b,D_{2}}=V_{d,j}-V_{b,D_{1}}$. Assign to one of these a molecule number distributed according to the Binomial distribution (proteins) and the multi‐hypergeometric distribution (targets and target configurations), depending on the mother’s molecule count *x_j_* and the daughter’s size ratio to the mother cell $V_{b,D_{2}}/V_{d,j}$, and assign the remaining molecules to the other daughter. Assign to each daughter independently a growth rate *µ_i_*, division volume *V_d,i_* and compute corresponding division time. To ensure a constant population size, randomly select a cell to be deleted from the population.

#### Repeat

Repeat from 2. Until *t* = *t_final_*.

### Molecular mechanism and model parameters

Each cell in the population contains a pool of biochemical species that can interact with each other through biochemical reactions, as described in step 2. We distinguish between the targets *P*, the CRISPR array *A*, which codes for a spacer *crRNA* matching a sequence on the target, and the surveillance protein *Cascade*. Together with the crRNA, the Cascade protein makes up the effector complex *E*. When the effector complex encounters a target it can bind, albeit with a low affinity in the case of a non‐consensus PAM on the target, forming a complex *EP*. Destruction of the target can then take place, producing DNA fragments *F*. One of these fragments can be integrated into the CRISPR array *A* as a new spacer, transforming the array to *A^∗^* which can now also express the newly acquired crRNA, *crRNA^∗^*, in addition to the spacer that was already present. The effector complex containing the new spacer, *E^∗^* has a higher binding affinity for the target. These biochemical reactions are governed by the equations described in Appendix Table S4.

The size of individual cells increases exponentially with a constant elongation rate throughout the cell cycle. Cellular length is used as a measure for cell size, as *E. coli* cell width remains approximately constant throughout the cell cycle and thus the cellular volume is linearly proportional to the cell length (Taheri‐Araghi *et al*, 2015). Growth parameters were chosen to be representative for our experimental data. As no kinetic data are available on individual reactions of the adaptation and interference processes, these parameters were calibrated to qualitatively agree with the experimentally determined target loss time distributions from the direct interference and priming conditions and previously published abundances of *cas* abundances (Djordjevic *et al*, 2012). Unless stated otherwise, the growth parameters used were $\mu_{p}=\frac{\log(2)}{70}$, *σ_p_* = 0.2, $\mu_{V_{B}}=0.5$, $\sigma_{V_{B}}=0.07\cdot \mu_{V_{B}}\;$, $\mu_{V_{D}}=3.9$, $\sigma_{V_{D}}=0.11\cdot \mu_{V_{B}}\;$, *p* ^s^ = 5. The other parameters used in the model simulations describing the kinetic reaction rates are given in Appendix Table S5. To simulate the direct interference condition with the same model, we simply modify the initial state of the system such that the spacer array consists of crRNA*^∗^*, which is flanked by the consensus PAM sequence.

### Cascade variability impacts the probability of spacer acquisition

In Fig 6C, we have shown that in priming, increased variability in the expression of Cascade can lead to faster spacer acquisition on average. In simulations of the agent‐based model, variability of the Cascade protein concentration is controlled through the protein production rate *k*_1_ in coordination with the average protein burst size *b_c_*: to modify Cascade variability while maintaining a constant concentration, *b_c_* is multiplied by a factor *a* while *k*_1_ is multiplied by its inverse, 1/*a*. In Fig 5, *a* = 100 which leads to an increase in the coefficient of variation of the Cascade concentration at steady state from *CV* = 0.02 (low Cascade variability) to *CV* = 0.42 (high Cascade variability).

We will now illustrate how higher Cascade variability can lead to faster spacer acquisition by considering two scenarios and comparing the cumulative probability of the time until spacer acquisition for the simplified two‐step model, which is given by.

$$FP_{\mathrm{SA}}\left(t|M_{0}\right)=1-e^{-M_{0}p_{p}\int_{0}^{t}m(t^{\prime })dt^{\prime }}$$

First, we consider a cell which has a constant Cascade level of 500 copies at any point in time between *t* = 0–1,000 min and plot the corresponding cumulative spacer acquisition probability (Appendix Fig S14A). Second, we consider a second cell in which Cascade is not constant but rather appears as a shorter “burst” of 2,500 copies from *t* = 200 min until *t* = 400 min, and 0 copies at any other time (Appendix Fig S14A). The cumulative spacer acquisition probability for the second cell reaches 1 faster than for the first cell (Appendix Fig S14B), despite the two cells having the same average Cascade concentration over the course of 1,000 min. This suggests that the effects of upwards fluctuations can outweigh the downward fluctuations.

where *p_d_* is the compound probability that within the time interval *∆t* a Cascade molecule binds to a target and the target is subsequently removed from the system.

### Model implementation

Stochastic simulations were performed using the adapted *Extrande* algorithm (Voliotis *et al*, 2016) implemented in C++. Each data point in Figs 5I and J and 6A–C was obtained from 100 simulated experiments of up to 10^4^ min. The population size of each simulation was fixed at 100 cells. See Materials and Methods and Appendix Tables S4‐S5 for model details and parameters.

## Author contributions

**Rebecca E McKenzie:** Conceptualization; Data curation; Formal analysis; Funding acquisition; Validation; Investigation; Visualization; Methodology; Writing – original draft; Writing – review & editing. **Emma M Keizer:** Data curation; Software; Formal analysis; Investigation; Visualization; Methodology; Writing – original draft; Writing – review & editing. **Jochem N A Vink:** Data curation; Formal analysis; Investigation; Methodology; Writing – review & editing. **Jasper van Lopik:** Investigation; Writing – review & editing. **Ferhat Büke:** Data curation; Investigation; Writing – review & editing. **Vera Kalkman:** Investigation; Writing – review & editing. **Christian Fleck:** Software; Supervision; Methodology; Writing – review & editing. **Sander J Tans:** Conceptualization; Supervision; Funding acquisition; Methodology; Writing – review & editing. **Stan J J Brouns:** Conceptualization; Supervision; Funding acquisition; Writing – review & editing.

In addition to the CRediT author contributions listed above, the contributions in detail are:

REM, SJJB and SJT conceived the project. REM, JNAV, JVL and VK performed the experiments. EMK, JV, REM and FB analysed the data. EMK and CF performed the modelling. REM, EMK, SJJB, SJT and CF wrote the manuscript with input from all authors.

## Disclosure and competing interests statement

The authors declare that they have no competing interests that relate to the research described in this paper.

## Supporting information



Appendix

Expanded View Figures PDF

Movie EV1

Movie EV2

Source Data for Figure 2

## Data Availability

Data analysis was performed using custom MATLAB scripts, which can be found at https://github.com/TansLab/Tans_Schnitzcells. Scripts for lineage analysis and plotting were implemented in MATLAB and are available upon request. An implementation of the agent‐based model in C++ is available at https://git.wur.nl/Biometris/articles/CRISPR_ABM .
