# Supplementary material for: Single cell variability of CRISPR‐Cas interference and adaptation
Source: Mol Syst Biol. 2022 Apr 25;18(4):e10680. doi: 10.15252/msb.202110680 (PMC10561596; doi:10.15252/msb.202110680)
Supplement: Supplementary file 1 — Appendix [file MSB-18-e10680-s001.pdf]

Appendix to Single Cell Variability of CRISPR-Cas Interference and Adaptation

Rebecca E. McKenzie*, Emma M. Keizer*, Jochem N.A. Vink, Jasper van Lopik, Ferhat Büke, Vera Kalkman, Christian Fleck, Sander J. Tans, Stan J.J. Brouns

* These authors contributed equally

Contents

1 Appendix Figures 2

2 Appendix Table S1: Strains and plasmids used in this study 14

3 Appendix Table S2: Oligonucleotides used in this study 15

4 Appendix Table S3: Synthetic DNA G-block used in this study 17

5 Appendix Table S4: Overview of the reactions in the model for primed adaptation 20

6 Appendix Table S5: Reaction rates used in simulations 21

1 Appendix Figures

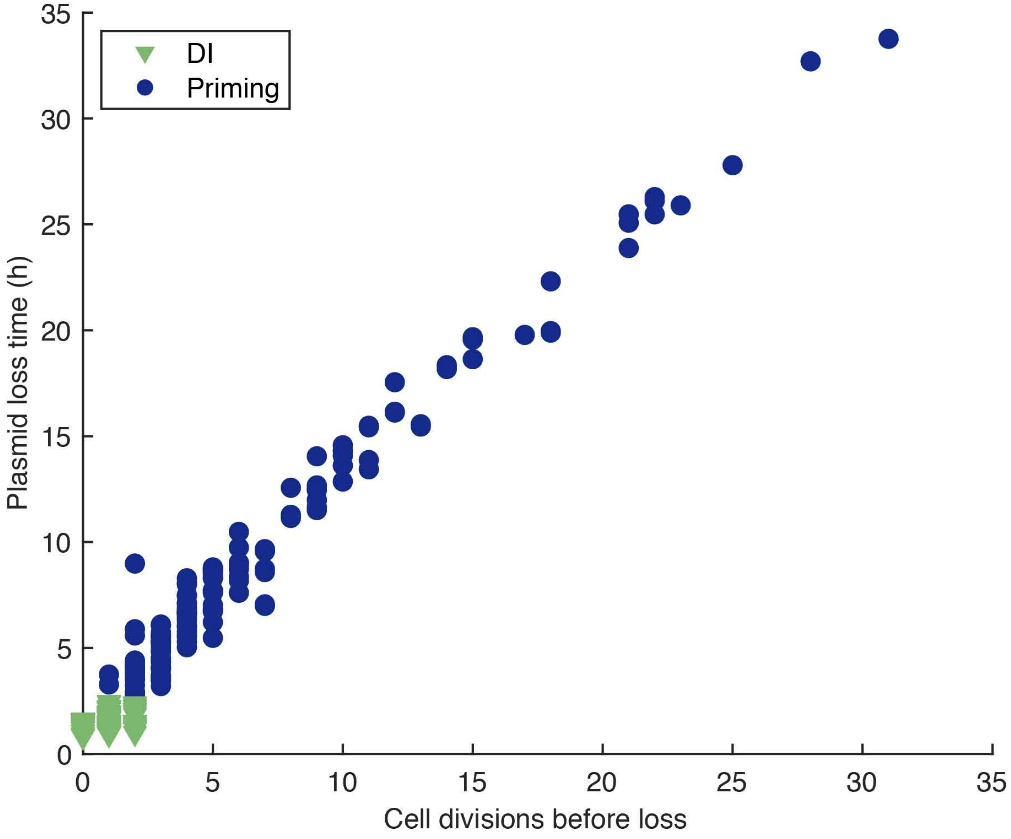

Appendix Fig S1: Cell divisions before plasmid loss highly correlates with plasmid loss time
The number of cell divisions from the moment of induction until plasmid loss are plotted against the PLT in hours. Both consensus target clearance by direct interference (green) and mutant target clearance by priming (blue) are shown.

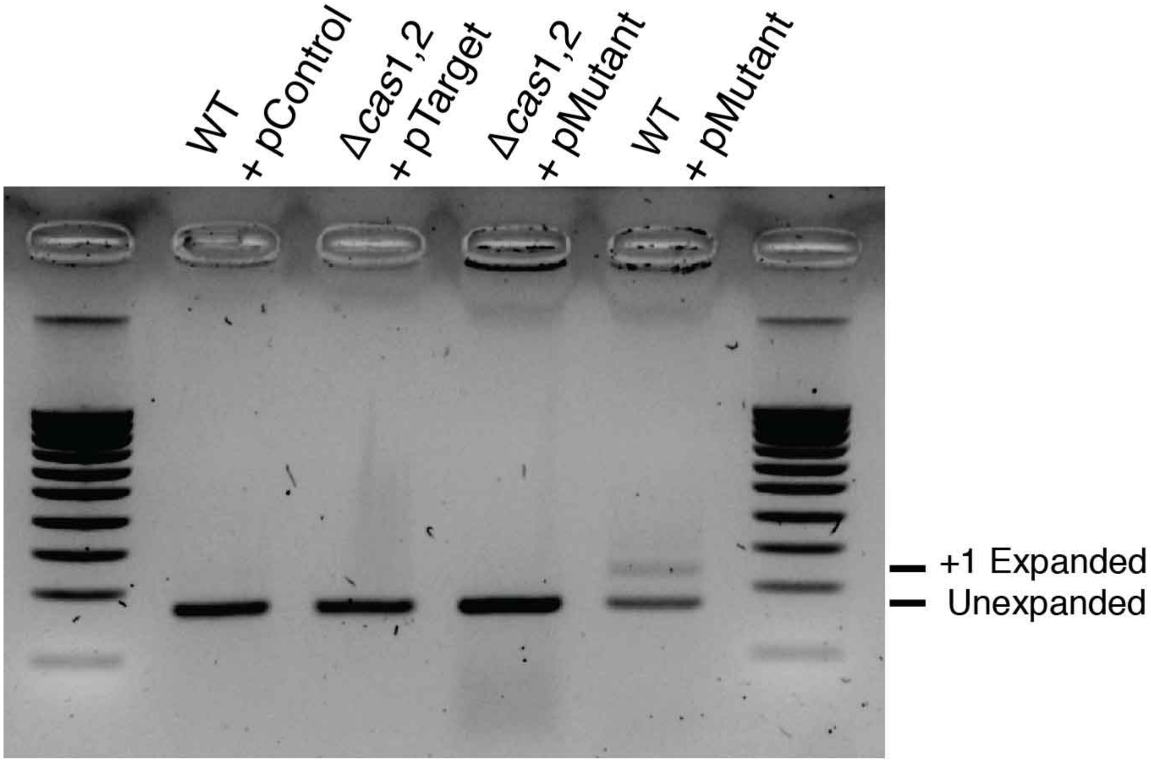

Appendix Fig S2: Spacer acquisition was only seen in the WT strain in the presence of a mutated PAM triggering priming

Cells from the chip output were collected in a flask for each experiment and the CRISPR arrays were screened for expansion due to spacer acquisition by PCR amplification using primers BN1530 + BN1531 (Appendix Table 2). The gel shows PCR amplified CRISPR arrays from each experiment. The presence of a larger band indicates array expansion and therefore successful spacer acquisition.

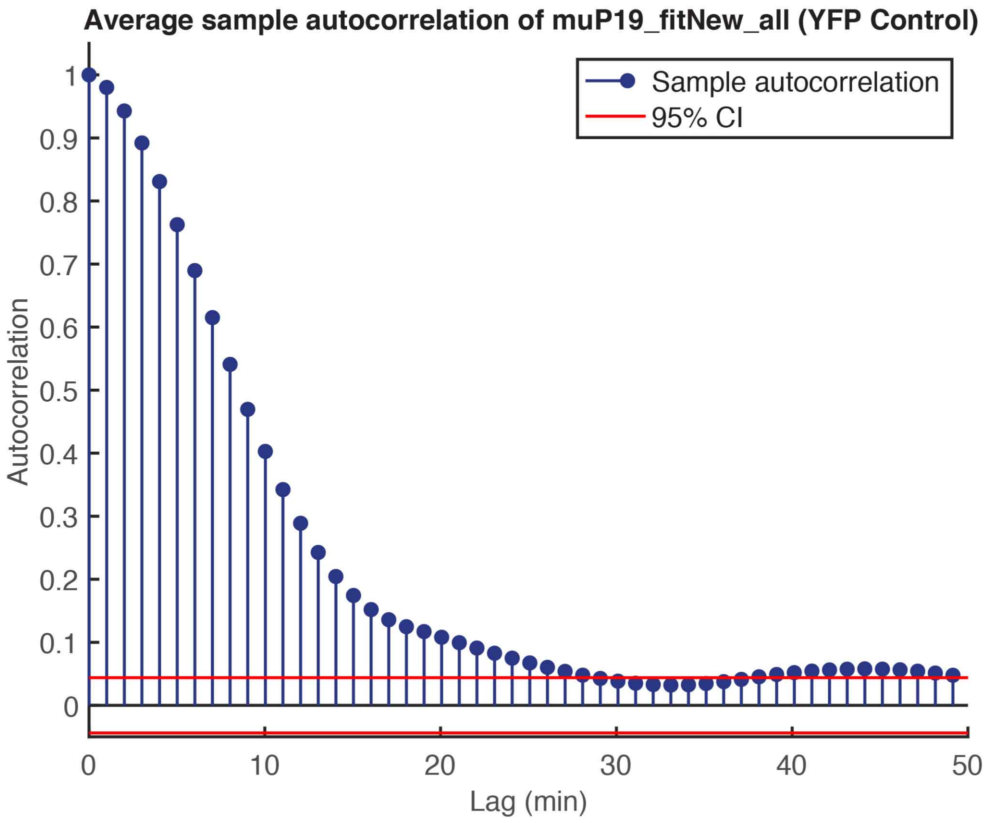

Appendix Fig S3: Autocorrelation time of cellular growth rate
The autocorrelation time was calculated for the cellular growth rate of the WT strain containing pControl by averaging the autocorrelation of cell growth as a function of time in individual lineages. After 10 minutes the autocorrelation of cellular growth has decreased to 0.4. After approximately 30 minutes the autocorrelation has decreased to zero, as indicated by 95% confidence intervals (red lines).

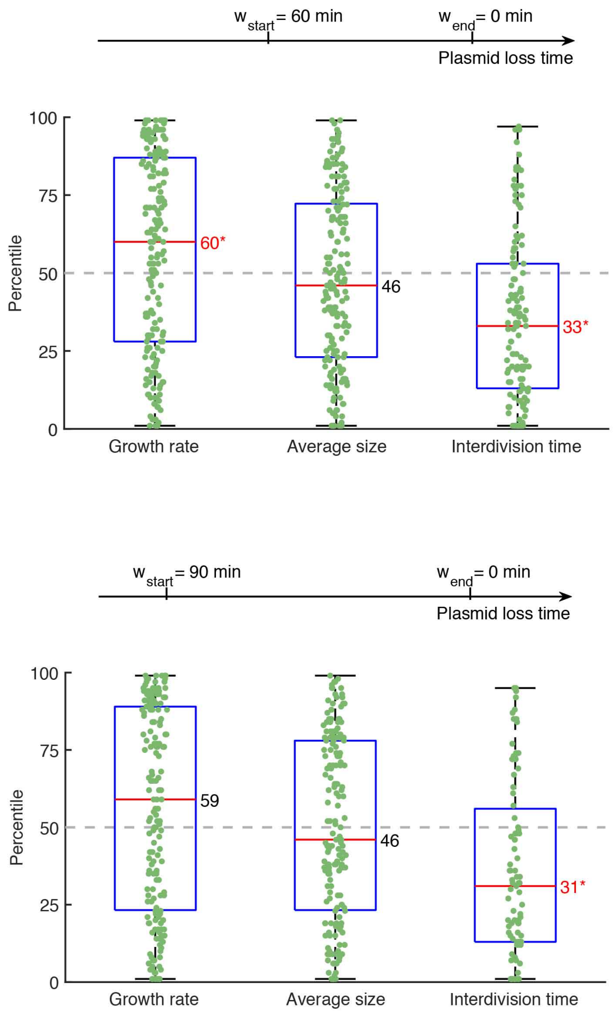

Appendix Fig S4: Growth rate, cell size and interdivision time of direct interference with different lookback windows

Boxplots of growth rate, average cell size and interdivision time presented as the percentile rankings of all plasmid loss lineages (green) that cleared a known target via direct interference. The cell feature of interest (e.g. growth rate) was averaged over a lookback window chosen in relation to the time from plasmid loss of the lineage of interest. The same cell feature was then averaged for all non-loss lineages in the population at that same moment. The cell feature of interest was then ranked amongst the non-loss population as a percentile. We considered lookback windows of 60 minutes prior to plasmid loss (top) and 90 minutes prior to plasmid loss (bottom). The median percentile ranking of loss lineages is indicated by a red line and black text, categories in which this value was significantly different from a ranking in the 50th percentile (p-value¡0.05) are indicated in red text followed by an asterisk.

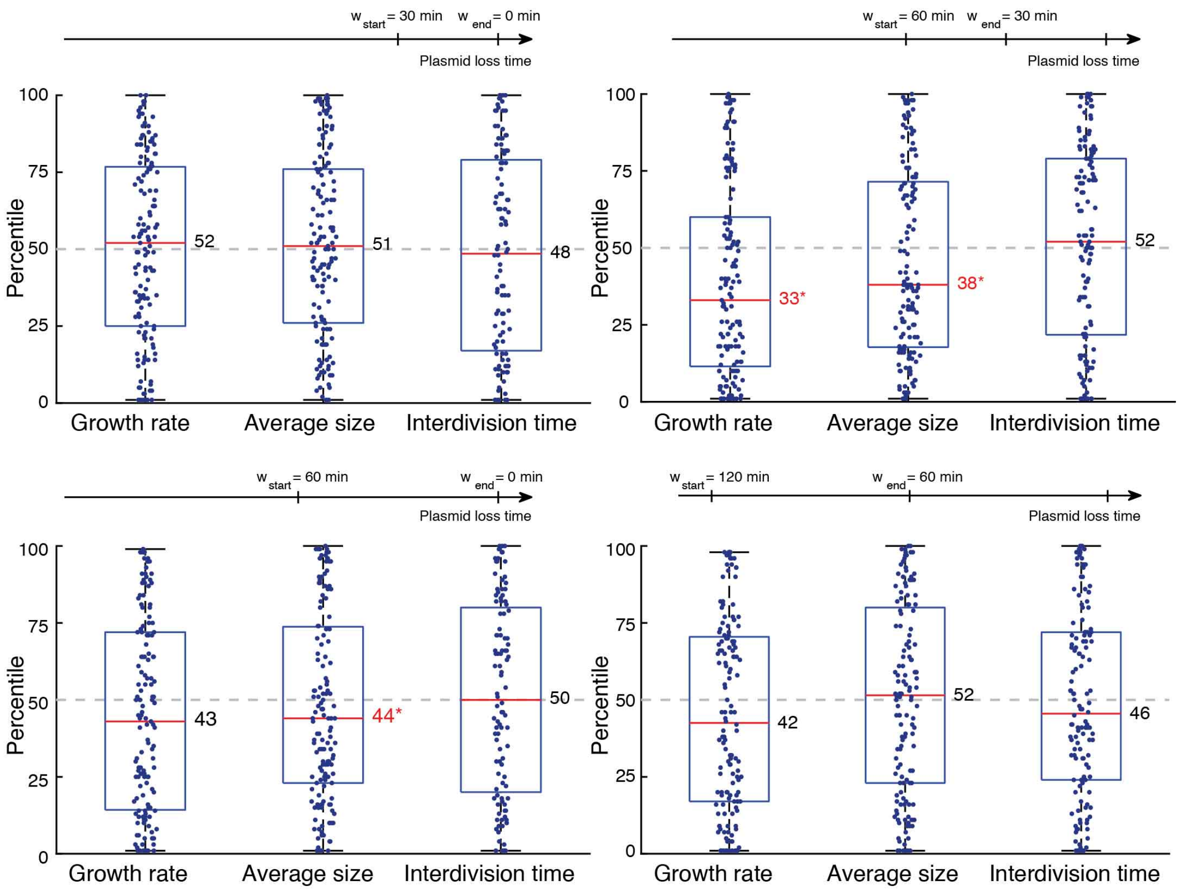

Appendix Fig S5: Growth rate, cell size and interdivision time of priming with different lookback windows

Boxplots of growth rate, average cell size and interdivision time presented as the percentile rankings of all plasmid loss lineages (navy) that cleared a known target via priming. The cell feature of interest (e.g. growth rate) was averaged over a lookback window chosen in relation to the time from plasmid loss of the lineage of interest. The same cell feature was then averaged for all non-loss lineages in the population at that same moment. The cell feature of interest was then ranked amongst the non-loss population as a percentile. We considered a range of lookback windows. The median percentile ranking of loss lineages is indicated by a red line and black text, categories in which this value was significantly different from a ranking in the 50th percentile (p-value ¡0.05) are indicated in red text followed by an asterisk.

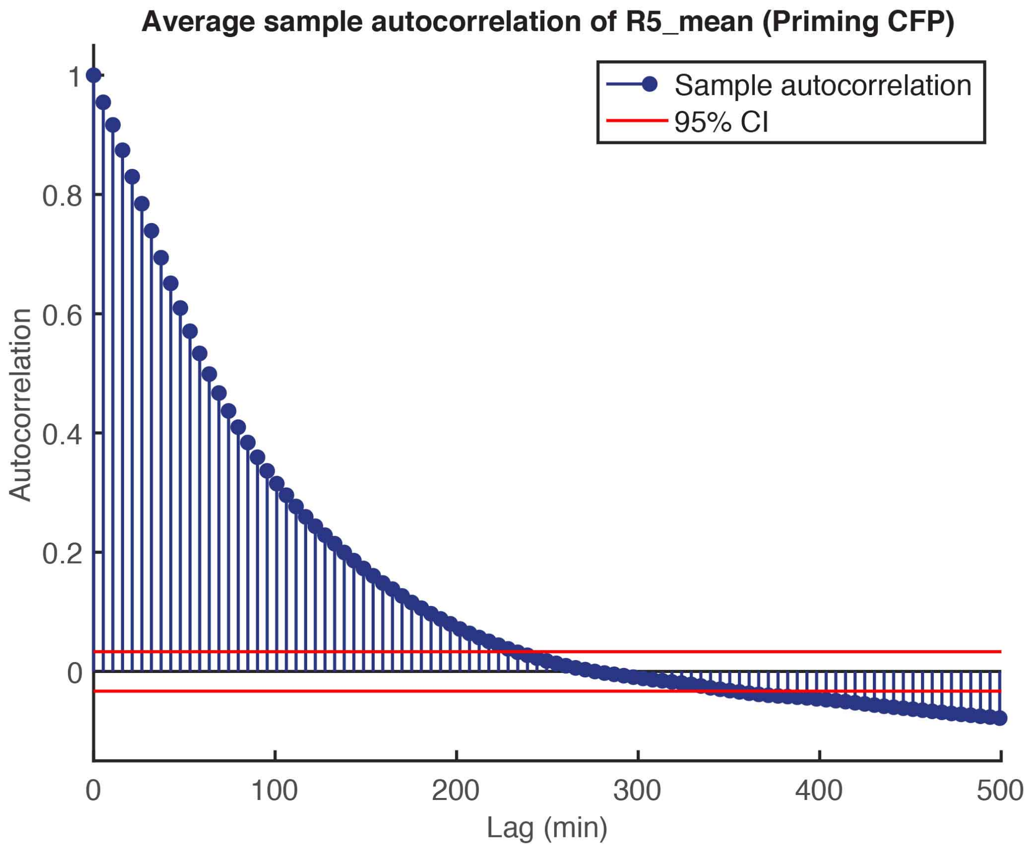

Appendix Fig S6: Autocorrelation of RFP (Cascade) concentration
The autocorrelation was calculated by averaging over the autocorrelation of RFP concentration of the WT-mCherry strain in individual lineages. After approximately 200 minutes the autocorrelation has decreased to zero, as indicated by 95% confidence intervals (red lines). The long decay time of the autocorrelation function indicates that Cascade protein levels fluctuate on a time scale longer than the cell cycle.

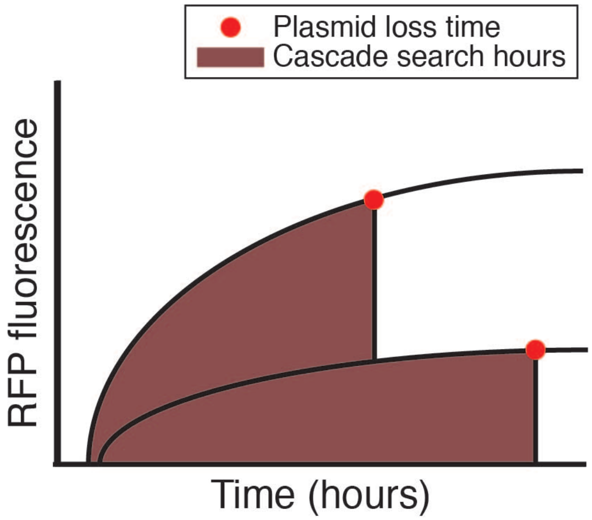

Appendix Fig S7: Cascade search hours are calculated from the cumulative RFP
Cascade search hours are the sum of all hours all Cascades have been searching in the cell. This can be calculated from the cumulative RFP or the area under the RFP curve as shown here in maroon. Two lineages with different RFP expression profiles are shown (black curves). The cell which has a higher RFP fluorescence and therefore copy number of Cascade loses the plasmid earlier as indicated by the red dot, while the cell with a lower copy number of Cascade loses the plasmid later. The two cells however, both lose the plasmid after approximately the same number of Cascade search hours i.e. the same area under the RFP curve.

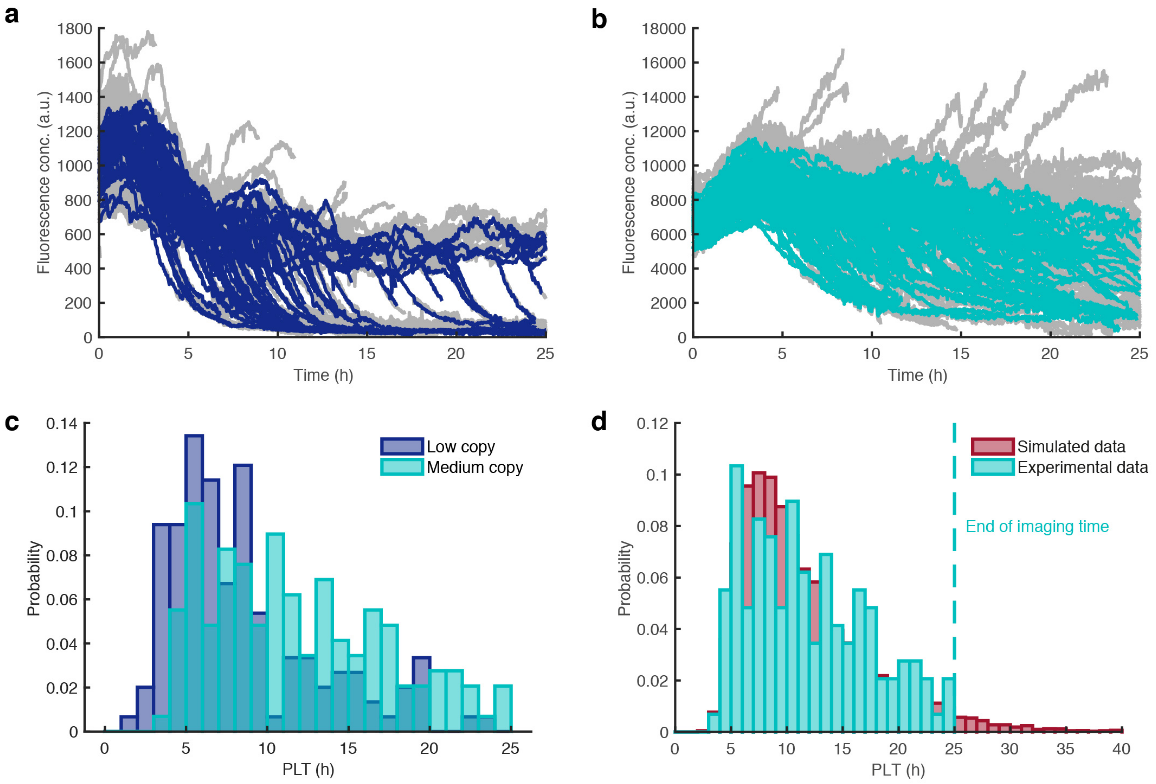

Appendix Fig S8: Plasmid loss through priming in cells with a low copy and medium copy plasmid

a, Reconstructed lineages traces of the imaged population (grey) of cells carrying a target with a mutated PAM and low copy number (TCN=5). Plasmid loss lineages are highlighted in navy. b, Reconstructed lineages traces of the imaged population (grey) of cells carrying a target with a mutated PAM and medium copy number (TCN=50). Plasmid loss lineages are highlighted in cyan. c, Plasmid loss time (PLT) distribution of the low copy and medium copy condition. d, PLT distributions of the experimental medium copy data (cyan) and simulated lineages with a target copy number of 50 (red).

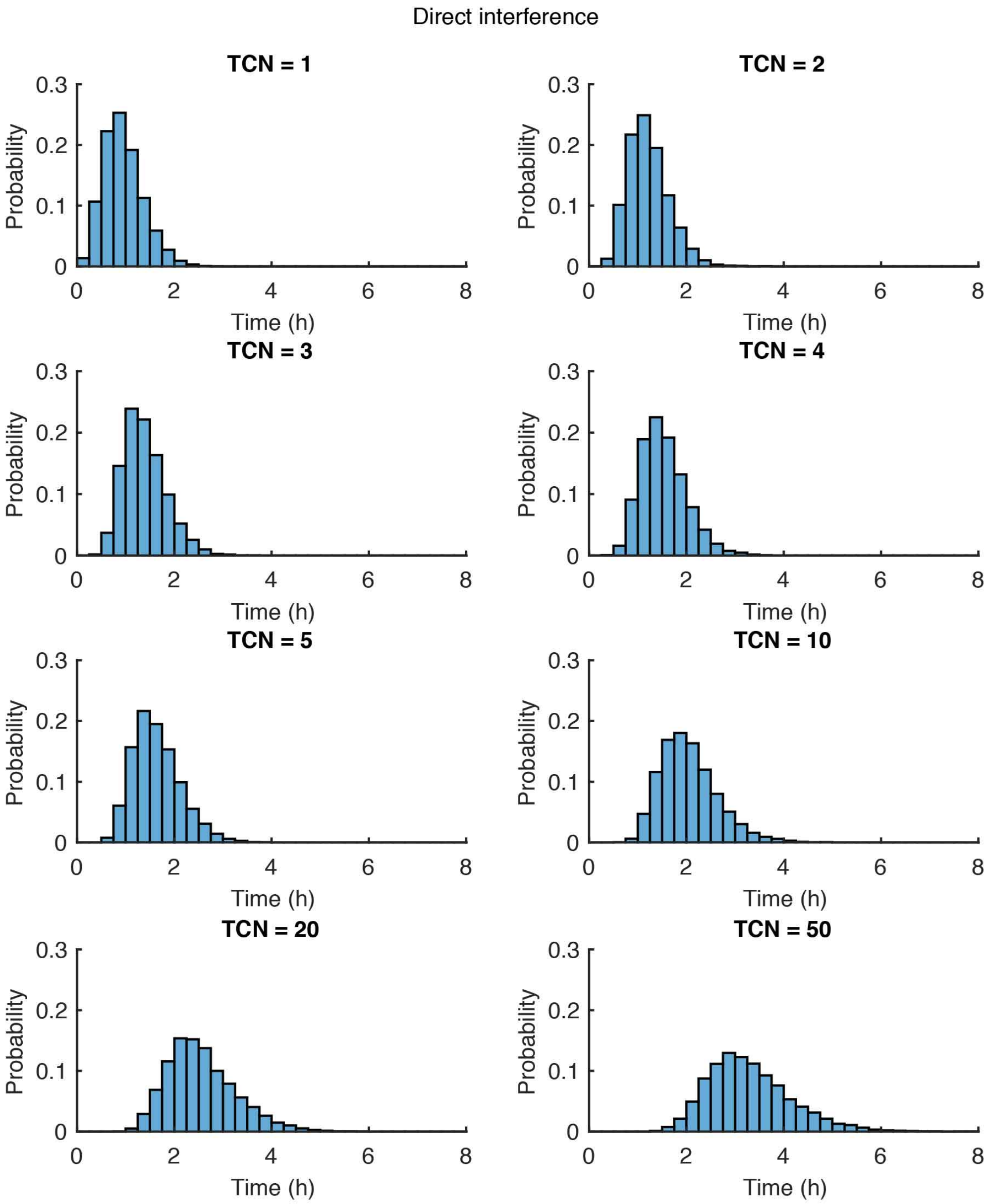

Appendix Fig S9: Distribution of target loss times resulting from simulations of the direct interference condition for average target copy numbers (TCN) per cell ranging from 1-50

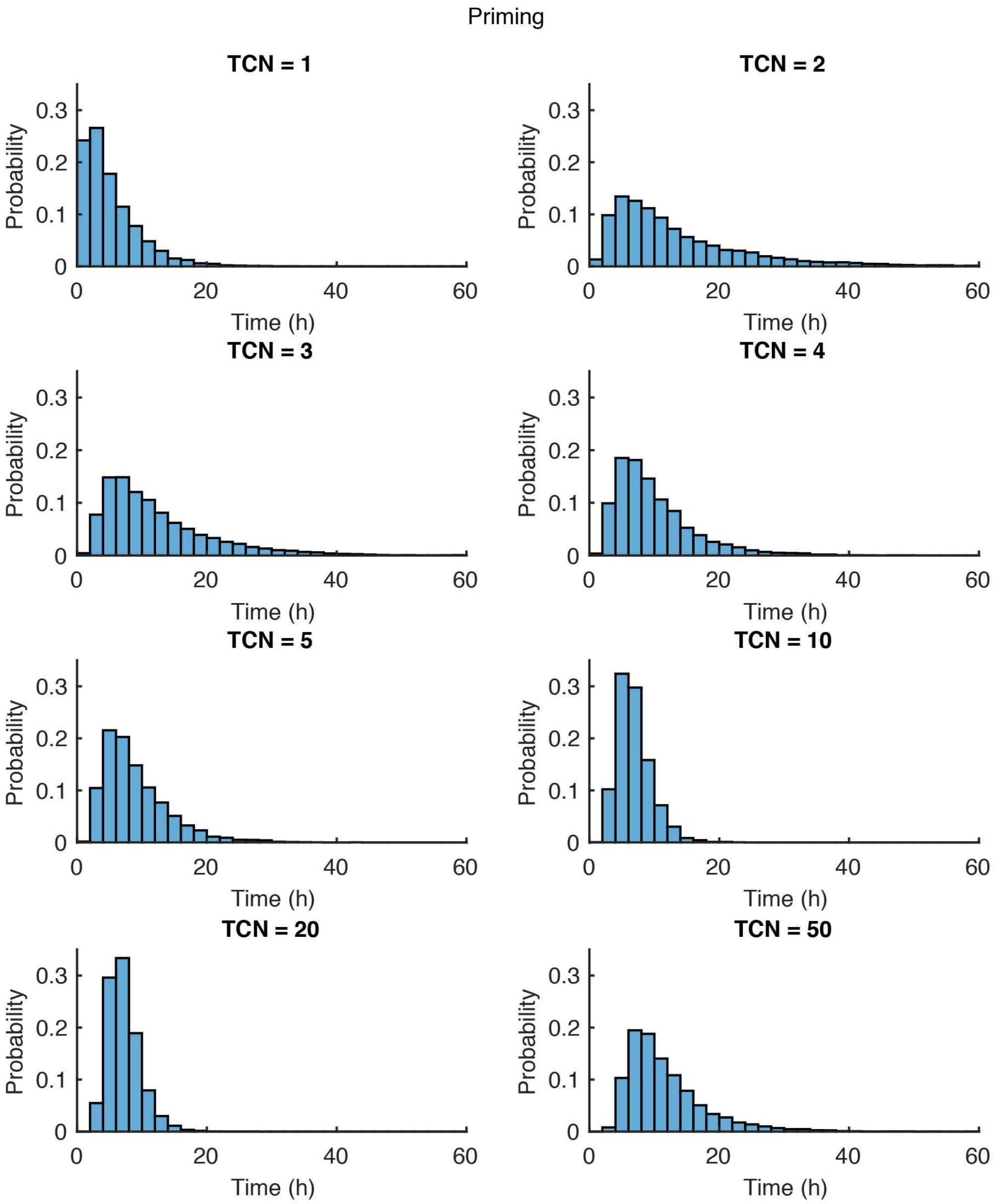

Appendix Fig S10: Distribution of target loss times resulting from simulations of the priming condition for average target copy numbers (TCN) per cell ranging from 1-50

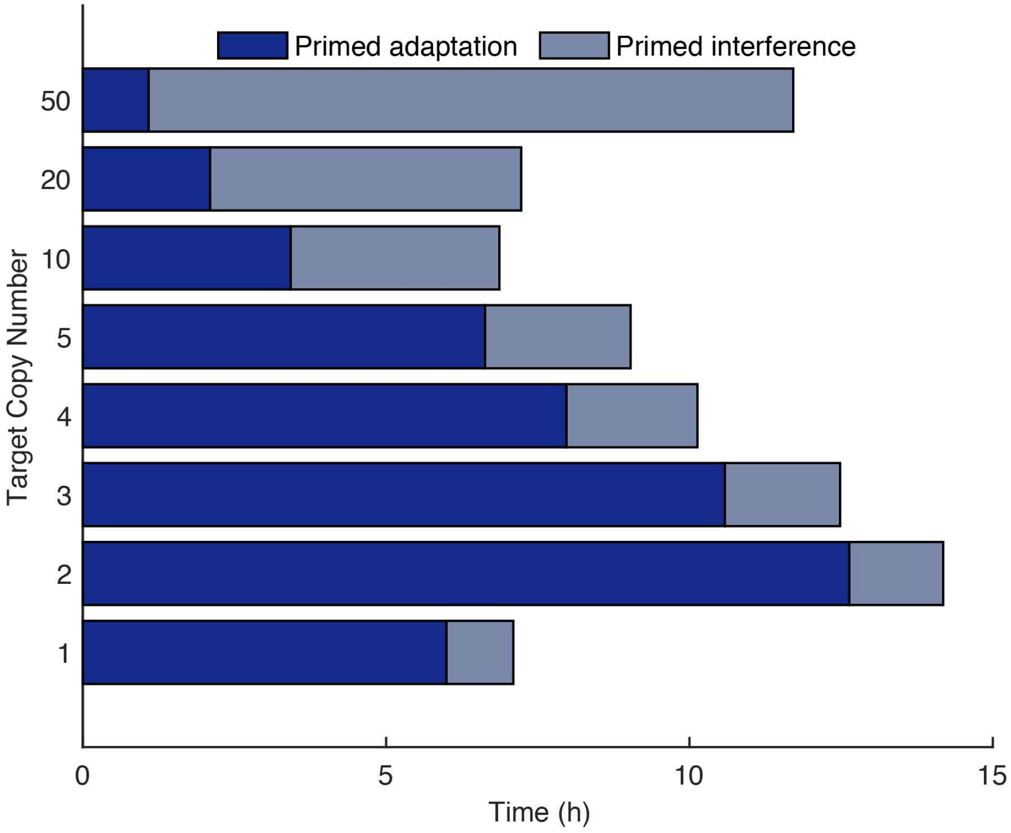

Appendix Fig S11: Target loss time as a function of the target copy number (TCN) as computed from simulated trajectories by the agent-based model for the priming condition Bar charts representing the time spent on primed adaptation (navy) and primed interference (grey) for cells clearing targets through priming with an average plasmid copy number ranging from 1-50.

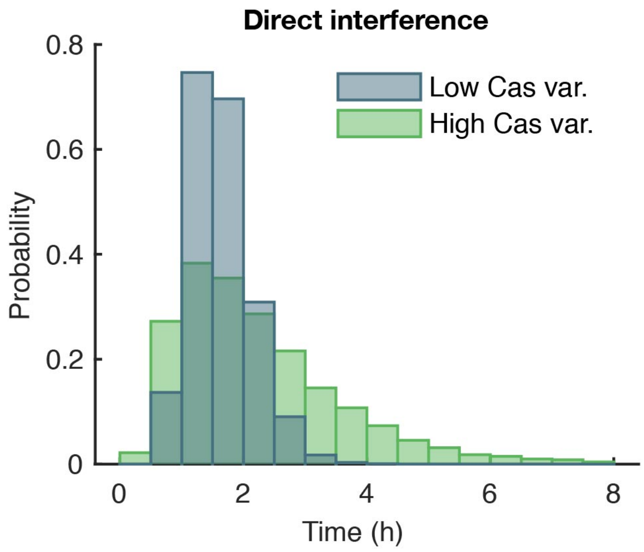

Appendix Fig S12: Distribution of plasmid loss times in direct interference for high and low variability in Cascade concentration

Target loss time distribution for two different levels of Cascade concentration variability resulting from simulated trajectories of the direct interference condition. At low variability (blue) Cascade proteins are produced in frequent, small bursts, whereas at high variability (green) proteins are synthesised more sporadically in large bursts (100-fold increase), keeping average Cascade concentration constant. The variability of PLT interference times for high Cascade variability increases as compared to low Cascade variability.

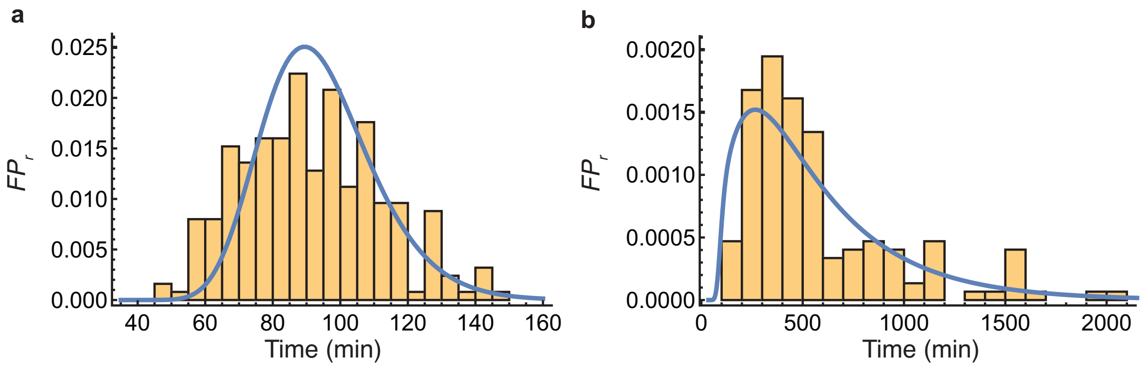

Appendix Fig S13: The fits of the simple one or two-step model to the data. **a**, fit of FP_r (solid line) to the target removal time in the case of direct interference. **b**, fit of FP_r (solid line) to the target removal time in the case of priming.

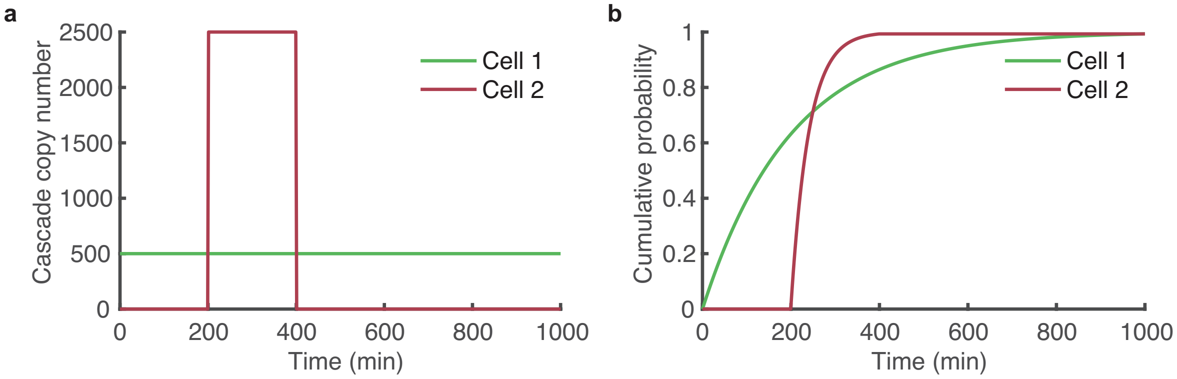

Appendix Fig S14: **a**, Cascade copy number of two cells with the same average over time. Cell 1 has a constant copy number of 500 Cascades, while in cell 2 Cascade is present transiently at 2500 copies between 200 and 400 minutes. **b**, Cumulative probability of time until spacer acquisition for the two cells with Cascade copy numbers as described in panel a ($M_0 = 1$, $p_p = 0.00001$).

2 Appendix Table S1: Strains and plasmids used in this study

**Strain**	**Description**	**Source**
KD615	*E. coli* K12, F+, ΔaraBAD, araBp8-*cse1*, lacUV5-*cas3*, CRISPR I R-SP8-R, ΔCRISPR II+III	(Datsenko et al., 2012; Musharova et al., 2019)
KD635	*E. coli* K12, F+, ΔaraBAD, araBp8-*cse1*, lacUV5-*cas3*, CRISPR I R-SP8-R, Δ*cas*1, 2, ΔCRISPR II+III	(Datsenko et al., 2012; Musharova et al., 2019)
KD615mCherry-Cas8e	*E. coli* K12, F+, ΔaraBAD, araBp8-*cse1*, lacUV5-*cas3*, CRISPR I R-SP8-R, ΔCRISPR II+III, mCherry-*cas8e*	This study
KD634mCherry-Cas8e	*E. coli* K12, F+, ΔaraBAD, araBp8-*cse1*, lacUV5-*cas3*, CRISPR I R-SP8-R,Δ*cas*1, 2, ΔCRSIPR II+III, mCherry-*cas8e*	This study
**Plasmid**	**Description**	**Source**
pTarget (pTU166)	pSC101, StrepR, TetR mVenus PS8 flanked by 'CTT' PAM	This study
pMutant (pTU190)	pSC101, StrepR, TetR mVenus PS8 flanked by 'CGT' PAM	This study
pControl (pTU193)	pSC101 ori, StrepR, TetR-mVenus, no target	This study
pVenus	pSC101 ori, KanR, mVenus-YFP	Bokinsky lab
pCDFDuet-1	pCloDF13 ori, StrepR	Lab collection
pTU265	pSC101, StrepR, TetR-Cerulean, no target	This study
pTU389	pSC101, StrepR, TetR-Cerulean, PS8 flanked by 'CGT' PAM	This study
pTU390	pSC101, StrepR, TetR-Cerulean, PS8 flanked by 'CTT' PAM	This study
pSC020	Derivative of pKD46 containing Lambda red and the Cre-recombinase	Lab collection

Appendix Table S1: Strains and plasmids used in this study

3 Appendix Table S2: Oligonucleotides used in this study

Appendix Table S2: Oligonucleotides used in this study. PAM sequences are indicated in bold, and restriction sites are underlined.

Name	Description	Sequence
BN831	Streptomycin resistance and PS8 insertion into pVenus, Fw	TTTTGGTACCTTATTT GCCGACTACCTTGGTG ATCTC
BN832	Streptomycin resistance and PS8 insertion into pVenus, Rv	TTTTAAGCTTAAAAG TGCCACTTGCGGAGA CCCGGTCGTCAGCTT ACATTCAAATATGTA TCCGCTC
BN833	Backbone amplification pVenus, Rv	TTTTGGTACCGGACTC TGGGGTTCGAG
BN834	Backbone amplification pVenus, Fw	TTTTAAGCTTCGAAAC GATCCTCATCCTG
BN891	Streptomycin resistance insertion (no target), Rv	TTTTAAGCTTACATTC AAATATGTATCCGCTC
BN911	Modify pTU166 PAM universal, Rv	TTTTGTCGACACATTC AAATATGTATCCGCTC ATGAGAC
BN912	Modify pTU166 CTT PAM to CGT	TTTTGTCGAC**ACG**CTG ACGACCGGGTC
BN1494	To amplify pTU193 Backbone minus yfp, Rv	TTTCTCGAGTAAGGAT CTCCAGGCATC
BN1495	To amplify pTU193 Backbone minus yfp, Fw	TTTCTCGAGTAAGGAT CTCCAGGCATC
BN1507	To amplify Cerulean from p15A, Fw	TTTGAATTCCAGAATT CAAAAGATCTAGGAGG
BN1508	To amplify Cerulean from p15A, Rv	TTTCTCGAGAGGATCC TTATTTATACAGCTCAT CC
BN1513	To check Cerulean insertion and confirm pTU265 by sequence, Fw	CCTCATTAAGCAGCTC TAATGCGCTG
BN1530	To screen for CRISPR array amplification, Fw	GGTTTGAAAATGGGAG CTCG
BN1531	To screen for CRISPR array amplification, Rv	GTTACATTAAGGTTGG TGGGTTG
BN2202	To amplify mCherry-Cas8e gblock, Fw	ACAGAATCTGGATGGA TGG
BN2203	To amplify mCherry-Cas8e gblock, Rv	CTGATCTCTACTGCAGT ATAGC
BN2204	Screen for mCherry-cas8e knock in, Fw	GCGCTTGCACTTAATCGC

Continued on next page

Appendix Table S2: Oligonucleotides used in this study. PAM sequences are indicated in bold, and restriction sites are underlined. (Continued)

BN2205	Screen for mCherry-cas8e knock in, Rv	ACCAGCAGTGCTAAAGCG
BN2206	Screen for mCherry-cas8e knock in, Fw	CTTTCCGTCCGGTGTC AGG
BN2275	Insertion PS8 CGT PAM into pTU265, Fw	TTTCCATGGAAAAGTG<br>CCACTTGCGGAGACCC<br>GGTCGTCAG**CGT**ACA<br>TTCAAATATGTATCCGC TCAT
BN2276	Insertion PS8 CTT PAM into pTU265, Fw	TTTCCATGGAAAAGTG<br>CCACTTGCGGAGACCC<br>GGTCGTCAG**CTT**ACAT<br>TCAAATATGTATCCGCT CAT
BN2278	Insertion of PS8 and PAM universal, Rv	TTTCCATGGCCTCATCC<br>TGTCTCTTGATC

4 Appendix Table S3: Synthetic DNA G-block used in this study

Name	Sequence
cas8e-mCherry insert	ACAGAATCTGGATGGATGGGTCTGGCAGGGTAACAGTA<br>TTGTTATTACCTATACAGGGGATGAAGGGATGACCAGAG<br>TCATCCCTGCAAATCCCAAATAACCTGGAGCTGCAGATA<br>CCGTTCGTATAATGTATGCTATACGAAGTTATAGATCTCTA<br>TTTGTTTATTTTTCTAAATACATTCAAATATGTATCCGCTC<br>ATGAGACAATAACCCTGATAAATGCTTCAATAATATTGA<br>AAAAGGAAGAGTATGAGCCATATTCAACGGGAAACGT<br>CTTGCTCTAGGC CGCGATTAAATTCCAACATGGATGC<br>TGATTTATATGGGTATAAATGGGCTCGCGATAATGTCGG<br>GCAATCAGGTGCGACAATCTATCGATTGTATGGGAAGC<br>CCGATGCGCCAGAGTTGTTTCTGAAACATGGCAAAGGT<br>AGCGTTGCCAATGATGTTACAGATGAGATGGTCAGACTA<br>AACTGGC TGACGGAATTTATGCCTCTTCCGACCATCAA<br>GCATTTTATCCGTACT CCTGATGACGCATGGTTACTCAC<br>CACTGCGATCCCCGGGAAAACAGCATTCCAGGTATTAG<br>AAGAATATCCTGATTCAGGTGAAAATATTGTTGATGCGCT<br>GGCAGTGTTCCTGCGCCGGTTGCATTCGATTCCTGTTT<br>GTAATTGTCCTTTTAACAGCGACCGCGTATTTCGTCTCGC<br>TCAGGCGCAATCACGAATGAATAACGGTTTGGTTGATGC<br>GAGTGATTTTGATGACGAGCGTAATGGCTGGCCTGTTGA<br>ACAAGTCTGGAAAGAAA TGCACAAACTTTTGCCATTCTC<br>ACCGGATTCAGTCGTCACTCATGGTGATTTCTCACTTGAT<br>AACCTTATTTTTGACGAGGGGAAATTAATAGGTTGTATTG<br>ATGTTGGACGAGTCGGAATCGCAGACCGATACCAGGAT<br>CTTGCCATCCTATGGAACTGCCTCGGTGAGTTTTCTCCTT<br>CATTA CAGAAACGGCTTTTTCAAAAATATGGTATTGATAAT<br>CCTGATATGAATAAATTGCAGTTTCATTTGATGCTCGATGA<br>GTTTTTCTAAGTCGACATAACTTCGTATAATGTATGCTATAC<br>GAACGGTAGAAATTGCAATGCATCTGCCGAATGCCGTGTG<br>GACGTAAGCGTGAACGTCAGGATCACGTTTCCCCGACCC<br>GCTGGCATGTCAACAATACGGGAGAACACCTGTACCGCC<br>TCGTTCGCCGCGCCACCATAAATCACCGCACCGTTCATC<br>AGTACTTTCAGATAACACATCG

Appendix Table S3: Synthetic DNA G-block used in this study

5 Appendix Table S4: Overview of the reactions in the model for primed adaptation

Phase	Reactions
Target replication	$P \xrightarrow{k_0/(1+((P/V_t)/p_0)^2)} 2P,$ $p_0 = \frac{p^*}{V_t}/\sqrt(\frac{k_0}{\mu} - 1)$
Expression	$G \xrightarrow{k_1(t)} G + b_P \cdot Cascade$ $k_1(t) = \frac{k_1}{1+exp(-k_d t)}$ <br><u>Before spacer integration</u> $A \xrightarrow{k_2} A + b_c \cdot crRNA$ <br><u>After spacer integration</u> $A^* \xrightarrow{k_2} A^* + b_c \cdot crRNA + b_c \cdot crRNA^*$ <br>$crRNA + Cascade \xrightarrow{k_3} E$ $crRNA^* + Cascade \xrightarrow{k_3} E^*$
Interference	$E + P \underset{k_5}{\overset{k_4}{\rightleftharpoons}} EP$ $E^* + P \underset{k_7}{\overset{k_6}{\rightleftharpoons}} EP^*$ $EP \xrightarrow{k_8} E + b_F \cdot F$ $EP^* \xrightarrow{k_8} E^* + b_F \cdot F$ $F \xrightarrow{k_9} \emptyset$
Primed adaptation	$F + A \xrightarrow{k_{10}} A^*$

Appendix Table S4: Overview of the reactions in the model for primed adaptation.

5 Appendix Table S5: Reaction rates used in simulations

**Reaction**	**Parameter**	**Value**
target replication	k_0	0.125 min^{-1}
Cascade production	k_1	2.4 min^{-1}
$crRNA/crRNA^*$ transcription	k_2	10 min^{-1}
$crRNA/crRNA^*$ degradation	k_3	0.014 min^{-1}
$crRNA - Cas/crRNA^* - Cas$ effector complex formation	k_4	0.01 $\text{M}^{-1}\text{min}^{-1}$
$E - P$ binding affinity	k_5	$1e^{-5}$ $\text{M}^{-1}\text{min}^{-1}$
EP dissociation	k_6	$1e^{-4}$ min^{-1}
$E^* - P$ binding affinity	k_7	$1e^{-3}$ $\text{M}^{-1}\text{min}^{-1}$
EP^* dissociation	k_8	$1e^{-4}$ min^{-1}
Target degradation	k_9	1 min^{-1}
Fragment degradation	k_{10}	1 min^{-1}
Spacer integration	k_{11}	0.25 $\text{M}^{-1}\text{min}^{-1}$
Cascade burst size	b_P	3
$crRNA/crRNA^*$ burst size	b_c	3
DNA fragment burst size	b_F	5
Post-induction delay of protein production	k_d	0.025 min^{-1}

Appendix Table S5: Reaction rates used in simulations.
