# Supplementary material for: Single cell variability of CRISPR‐Cas interference and adaptation
Source: Mol Syst Biol. 2022 Apr 25;18(4):e10680. doi: 10.15252/msb.202110680 (PMC10561596; doi:10.15252/msb.202110680)
Supplement: Supplementary file 2 — Expanded View Figures PDF [file MSB-18-e10680-s002.pdf]

Expanded View Figures

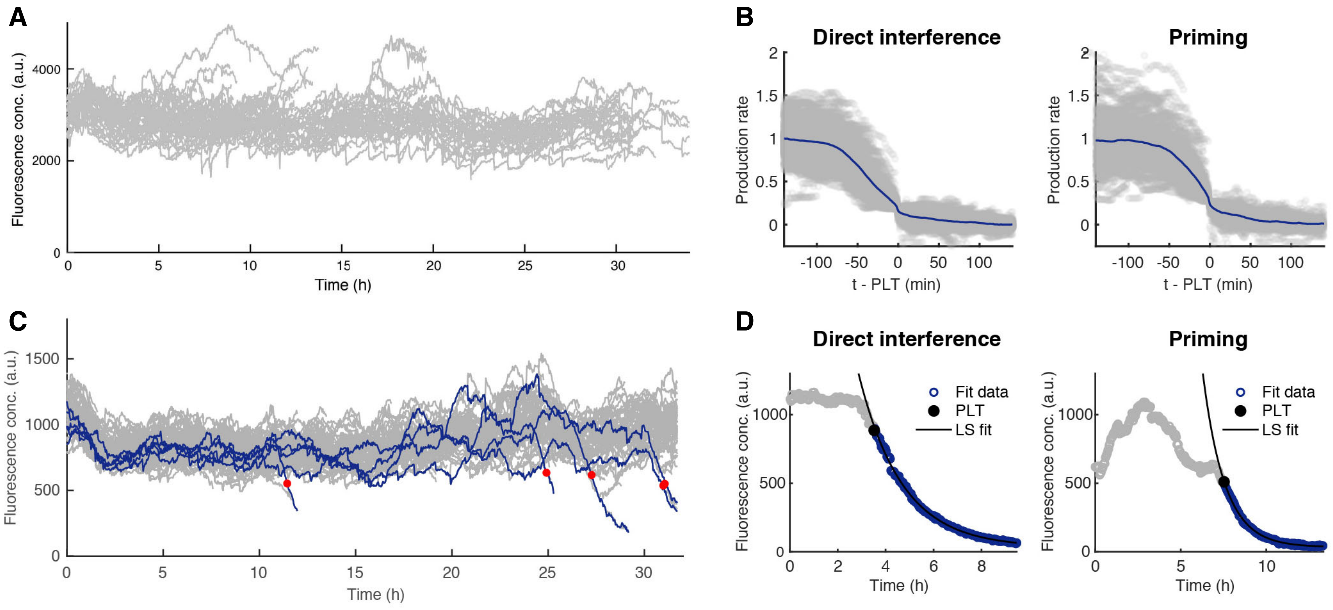

Figure EV1. Details of CRISPR-dependent plasmid loss and subsequent fluorescence decay.

A Plasmid loss is CRISPR-dependent. The YFP fluorescence traces in arbitrary units (a.u.) of the WT strain harbouring pControl a plasmid with no target for the CRISPR-Cas system. Time-lapse imaging was carried out for 35-h post-induction of the *cas* genes and showed no plasmid loss.

B Decay of YFP fluorescence in both direct interference and priming follows exponential decay. The fluorescence concentration data (open circles) of representative lineage of the direct interference (left) and priming (right) lineages can be described by exponential decay. This was evaluated by performing a least-squares (LS) fit of the fluorescence concentration data (purple open circles) after the moment of plasmid loss (PLT, black circle) to an exponential curve (LS fit, black line).

C In the absence of Cas1 and Cas2 clearance of a target with a non-consensus PAM mutant occurs rarely. The YFP fluorescence of the Δ*cas1,2* strain containing pMutant was imaged for 34 h after induction. Lineages that were able to clear the plasmid are highlighted in blue with the red dot indicating the PLT determined by the production rate. 1.4% of lineages (5 unique events, red dot) cleared the plasmid.

D Plasmid loss during direct interference and primed interference processes occur on a comparable timescale. All production rate traces (grey) starting from 140 min prior to the detected plasmid loss time PLT from direct interference (left) and priming (right) were aligned at the PLT (t-PLT = 0) and the average trend (navy) normalised for comparison. From the average trend, we estimate the clearance time (CT), time taken from the initiation of plasmid clearance until the elimination of all copies, to be in the order of 60 min for both direct interference and priming from the onset of the production rate decrease.

© 2022 The Authors

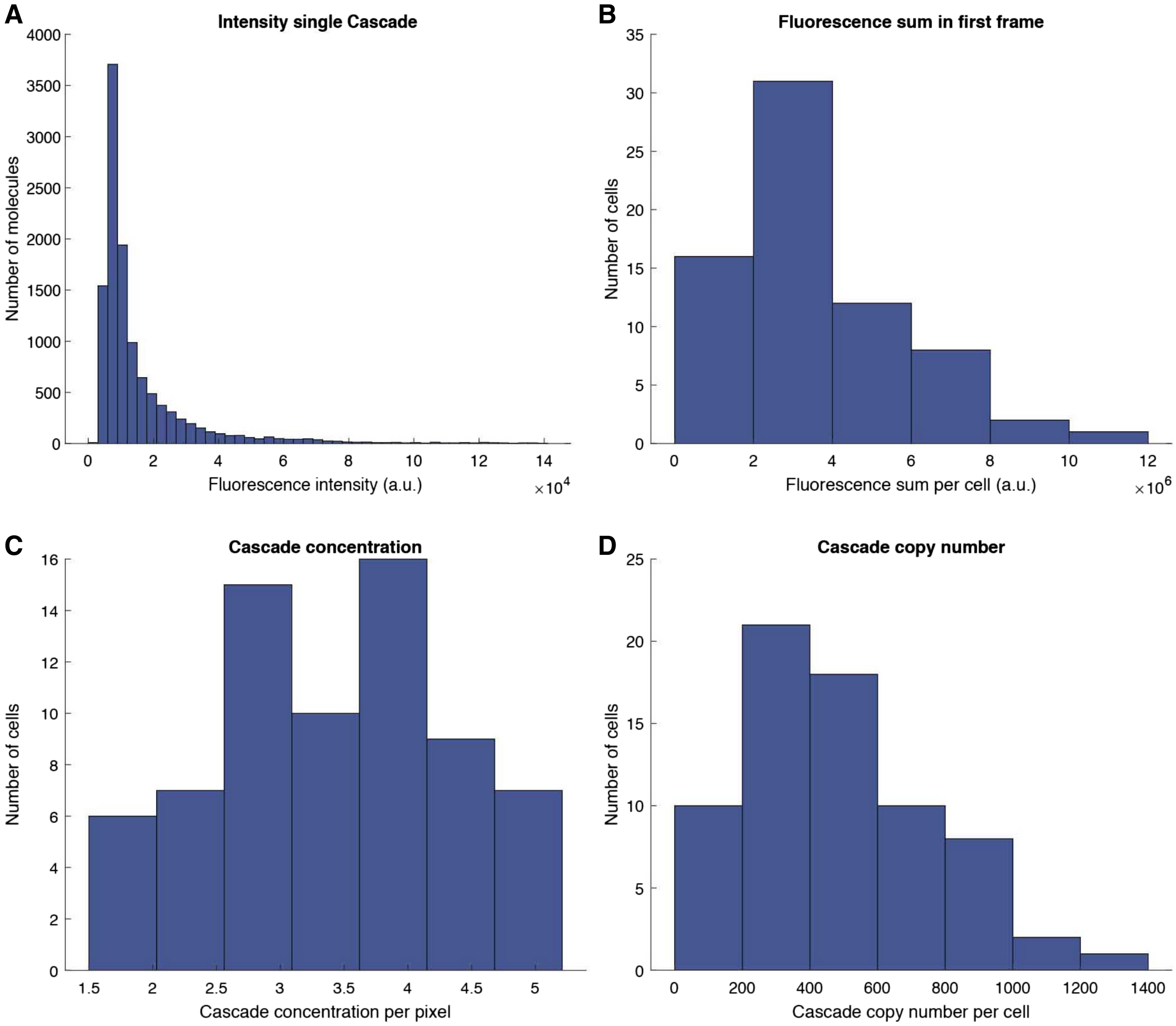

Figure EV2. Cascade copy number determination.

A The fluorescence sum (RFP) of each cell in the first frame was determined.
B The RFP molecules were then bleached until it was possible to determine the fluorescence intensity of a single molecule (representing a single Cascade).
C The Cascade copy number per cell was then determined by dividing the average fluorescence sum by the average intensity of a single Cascade molecule.
D The Cascade concentration per pixel was determined by dividing the fluorescence sum by the area of the cell in pixels.

 © 2022 The Authors

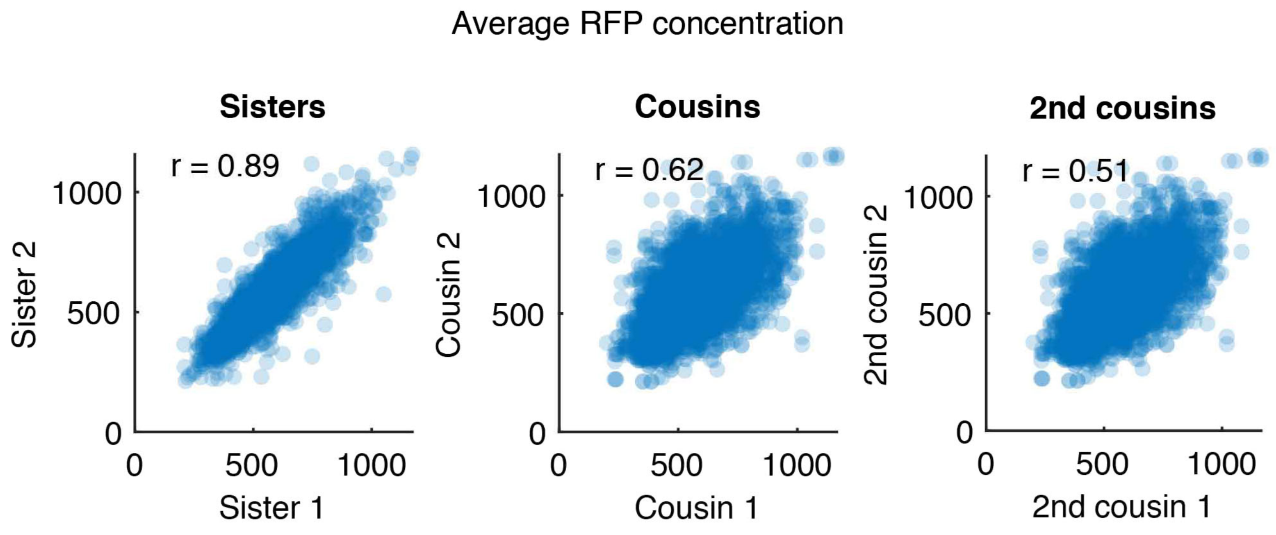

Figure EV3. Correlation of RFP levels between cells related as sisters, cousins and second cousins.

The levels of RFP (Cascade) are strongly correlated between sisters, cousins and second cousins. The correlation coefficient r decreases as the cells become less closely related.

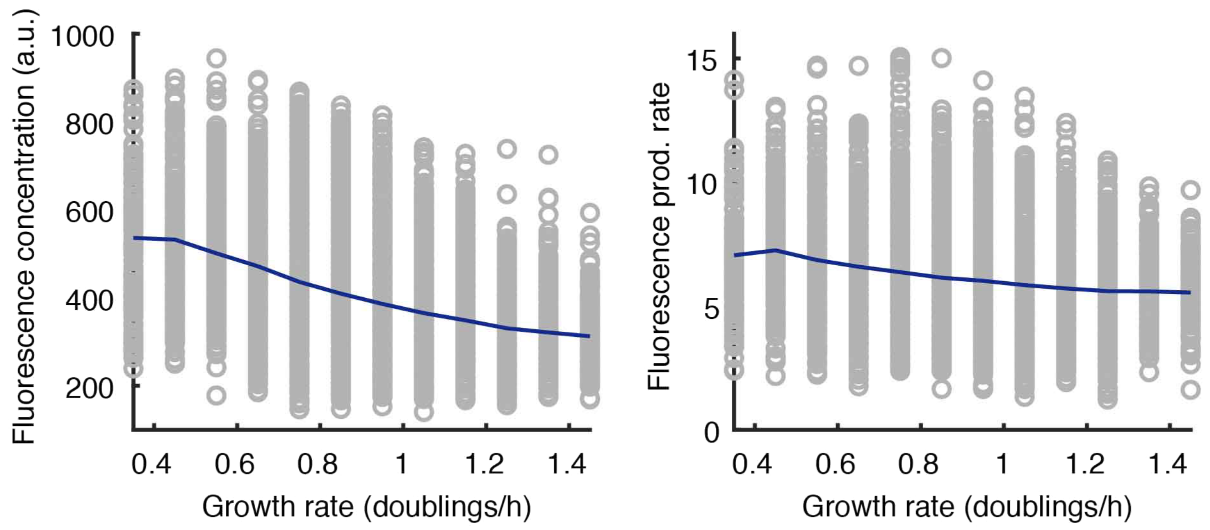

Figure EV4. Schematic representation of the agent-based simulation framework.

In this framework, we simulate a bacterial population, including exponential cell growth and stochastic division dynamics. At the level of individual cells, we simulate the stochastic reaction kinetics of all steps of the CRISPR defence process. The details of the simulation procedure are described in the Materials and Methods.

© 2022 The Authors

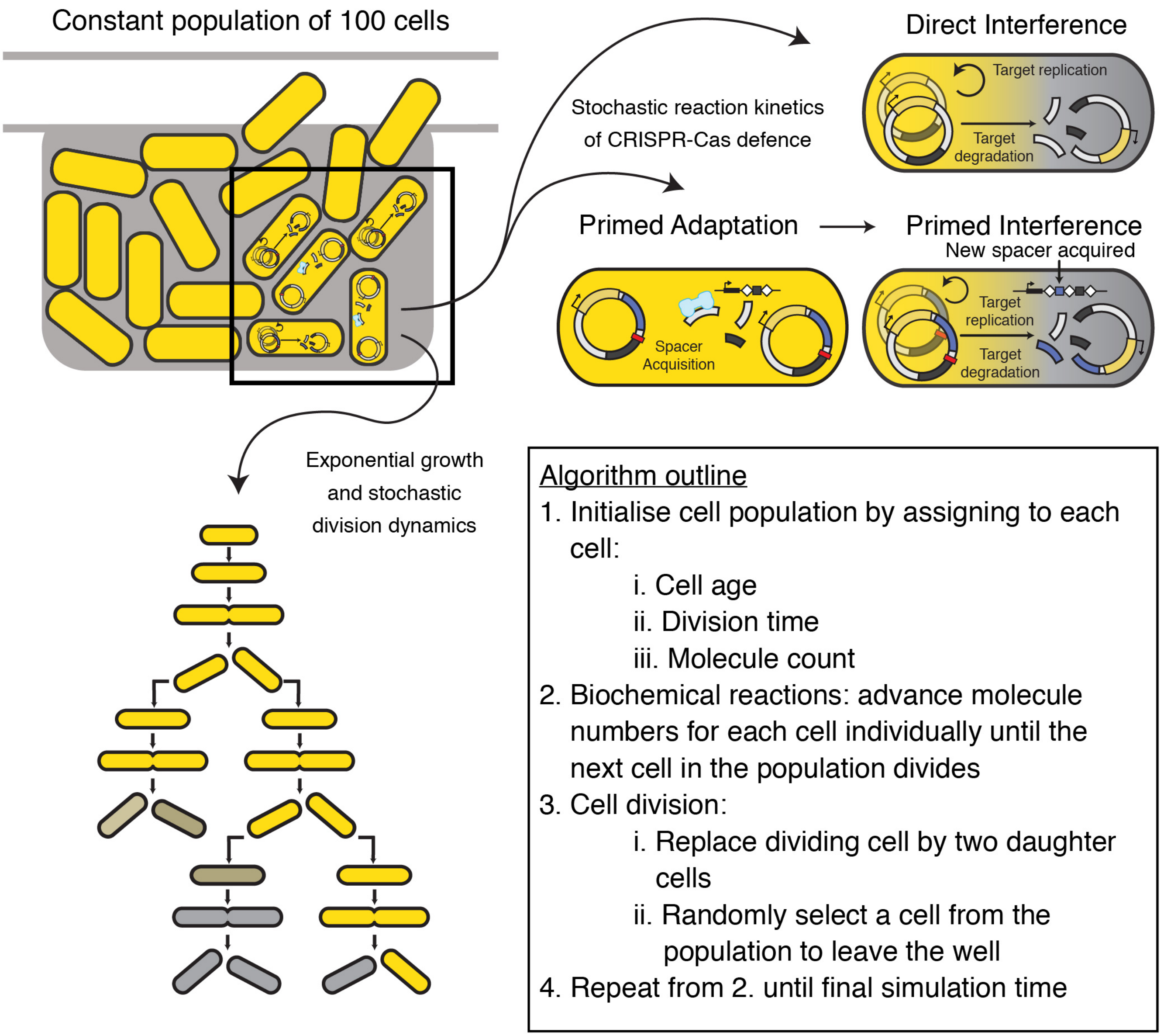

Figure EV5. Slower-growing cells have higher Cascade concentrations.

Cascade concentration (left) and Cascade production rate (right) show an inverse relationship with cellular growth rate (grey circles), revealing slower-growing cell on average (navy line) have a higher concentration of Cascade.

© 2022 The Authors
