# Supplementary material for: Single cell variability of CRISPR‐Cas interference and adaptation
Source: Mol Syst Biol. 2022 Apr 25;18(4):e10680. doi: 10.15252/msb.202110680 (PMC10561596; doi:10.15252/msb.202110680)
Supplement: Supplementary file 3 — Movie EV1 [file MSB-18-e10680-s003.zip › MSB-2021-10680R_MovieEV1/Legend_MovieEV1.docx]

**Movie EV1**

Depicts loss of the target plasmid with a consensus PAM encoding YFP in *E. coli* cells, due to direct interference. This movie is related to Fig 2A,b. A single chamber of the microfluidic chip is shown. Phase contrast and fluorescent images were overlaid and compiled at 2-minute intervals starting from induction (0 h). Time is indicated in the bottom right in hours.
