# Supplementary figures and images for: Single cell variability of CRISPR‐Cas interference and adaptation
Source: Mol Syst Biol. 2022 Apr 25;18(4):e10680. doi: 10.15252/msb.202110680 (PMC10561596; doi:10.15252/msb.202110680)

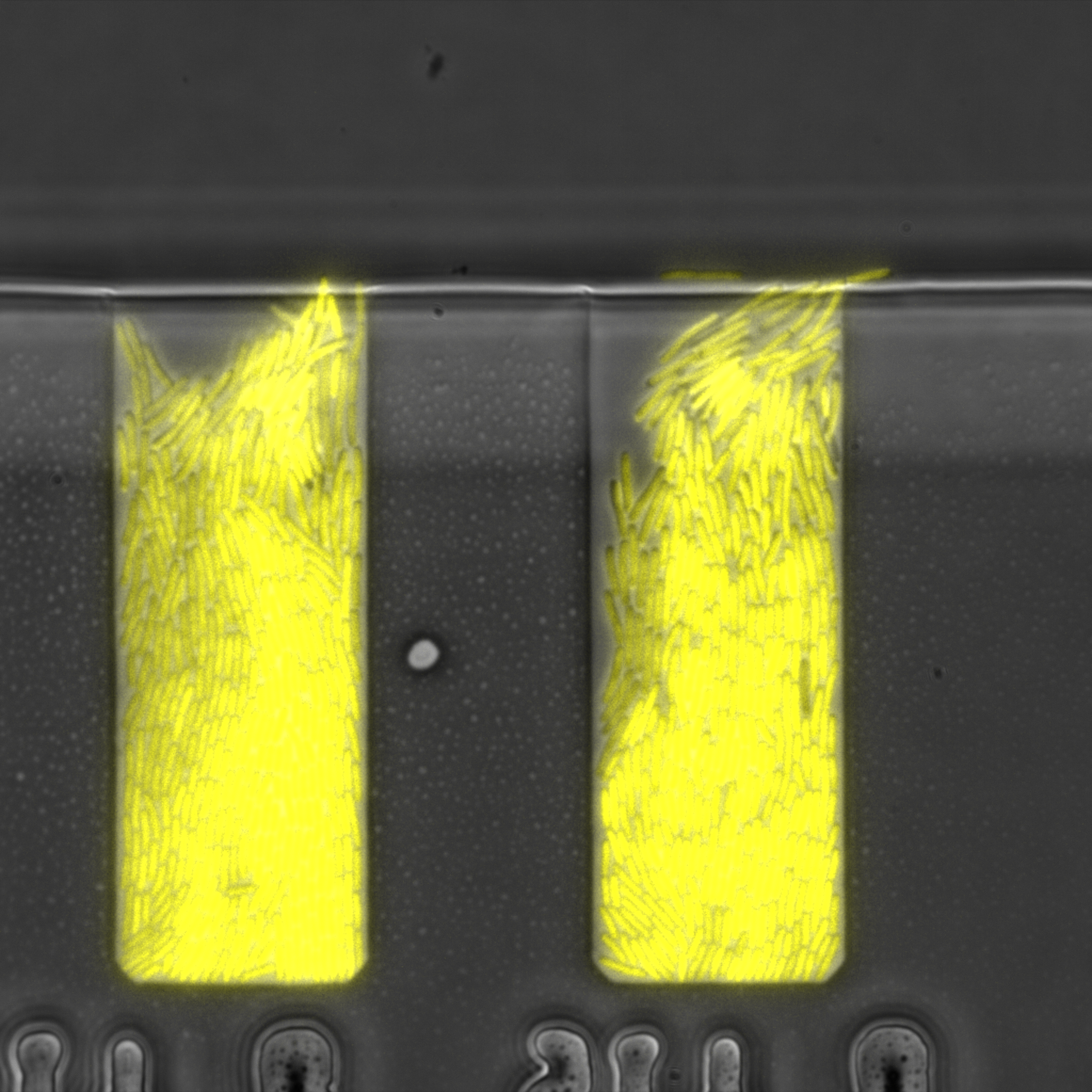

Supplement: Supplementary file 5 — Source Data for Figure 2 [file MSB-18-e10680-s004.zip › Fig2 panel 2a/Overlaid images used in illustrator/DIyfp1 (RGB).tif]

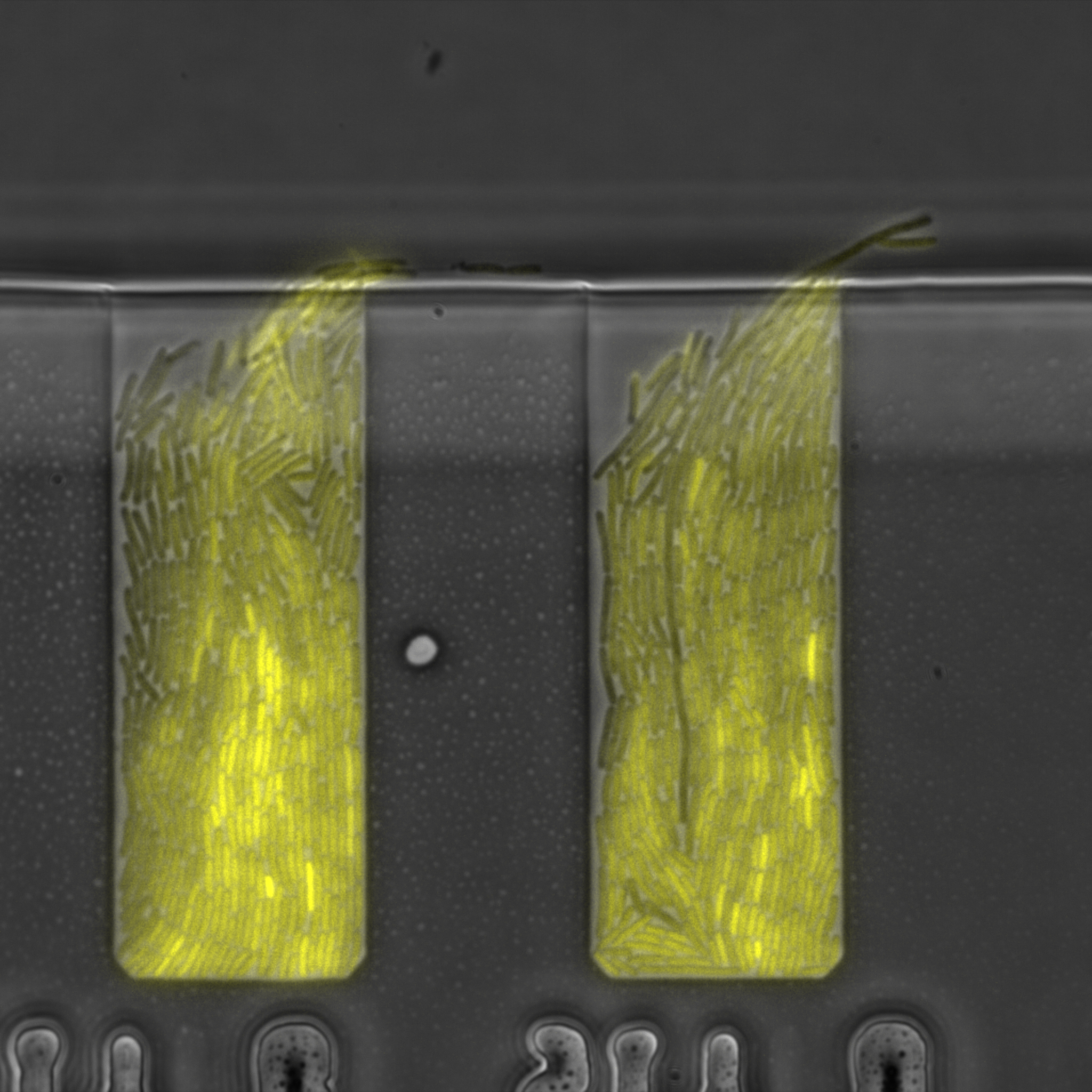

Supplement: Supplementary file 5 — Source Data for Figure 2 [file MSB-18-e10680-s004.zip › Fig2 panel 2a/Overlaid images used in illustrator/DIyfp2 (RGB).tif]

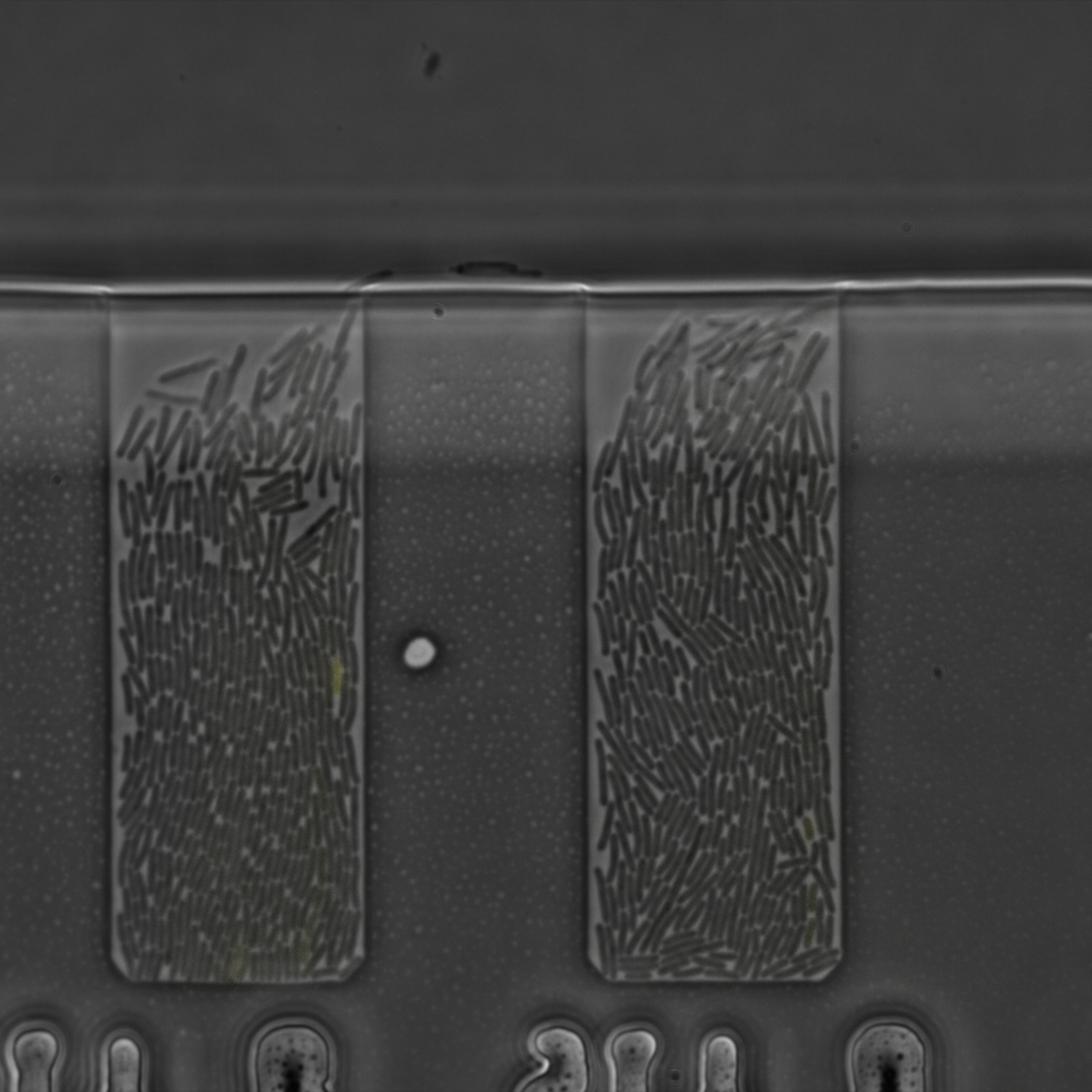

Supplement: Supplementary file 5 — Source Data for Figure 2 [file MSB-18-e10680-s004.zip › Fig2 panel 2a/Overlaid images used in illustrator/DIyfp4 (RGB).tif]

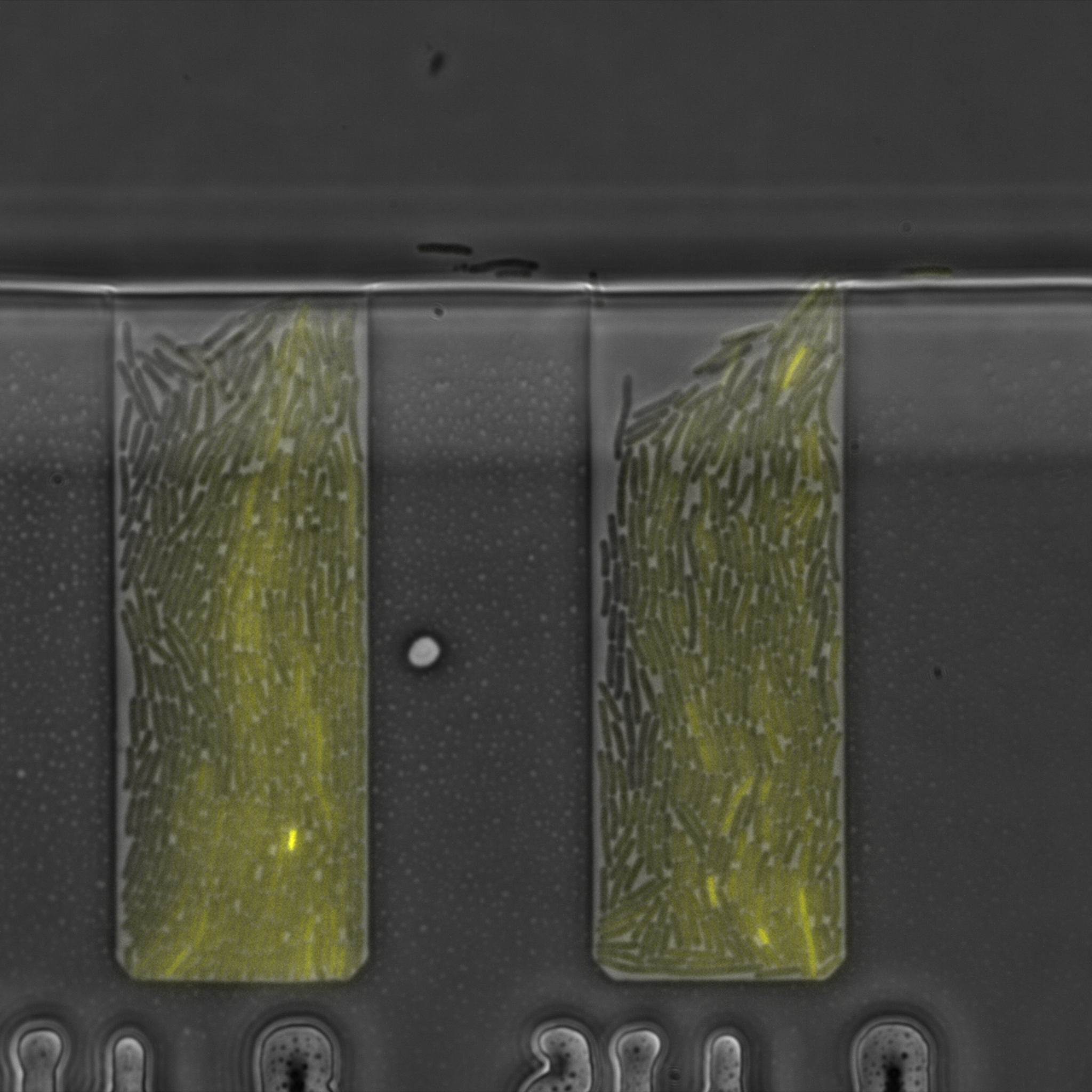

Supplement: Supplementary file 5 — Source Data for Figure 2 [file MSB-18-e10680-s004.zip › Fig2 panel 2a/Overlaid images used in illustrator/DIyfp3 (RGB).tif]

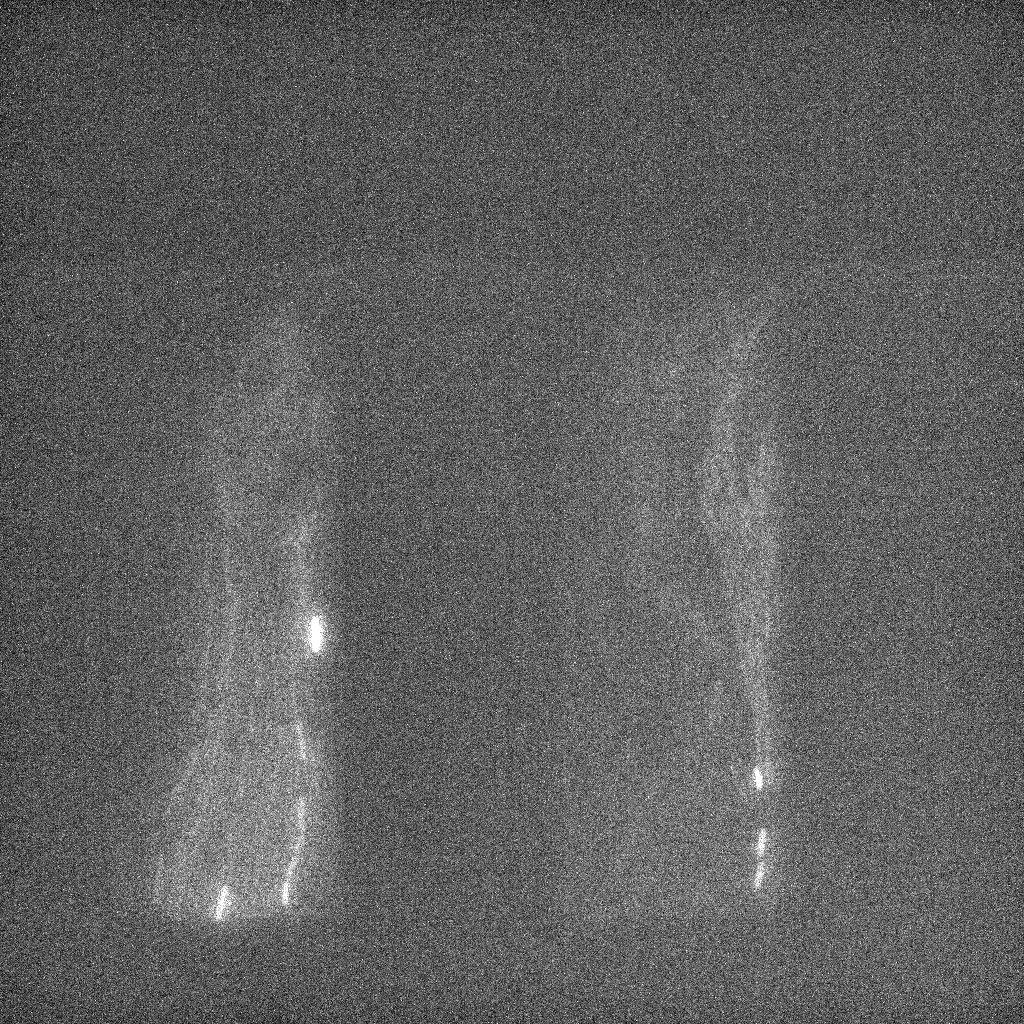

Supplement: Supplementary file 5 — Source Data for Figure 2 [file MSB-18-e10680-s004.zip › Fig2 panel 2a/Source images from the microscope/fluorescence unprocessed/pos2-y-4.tif]

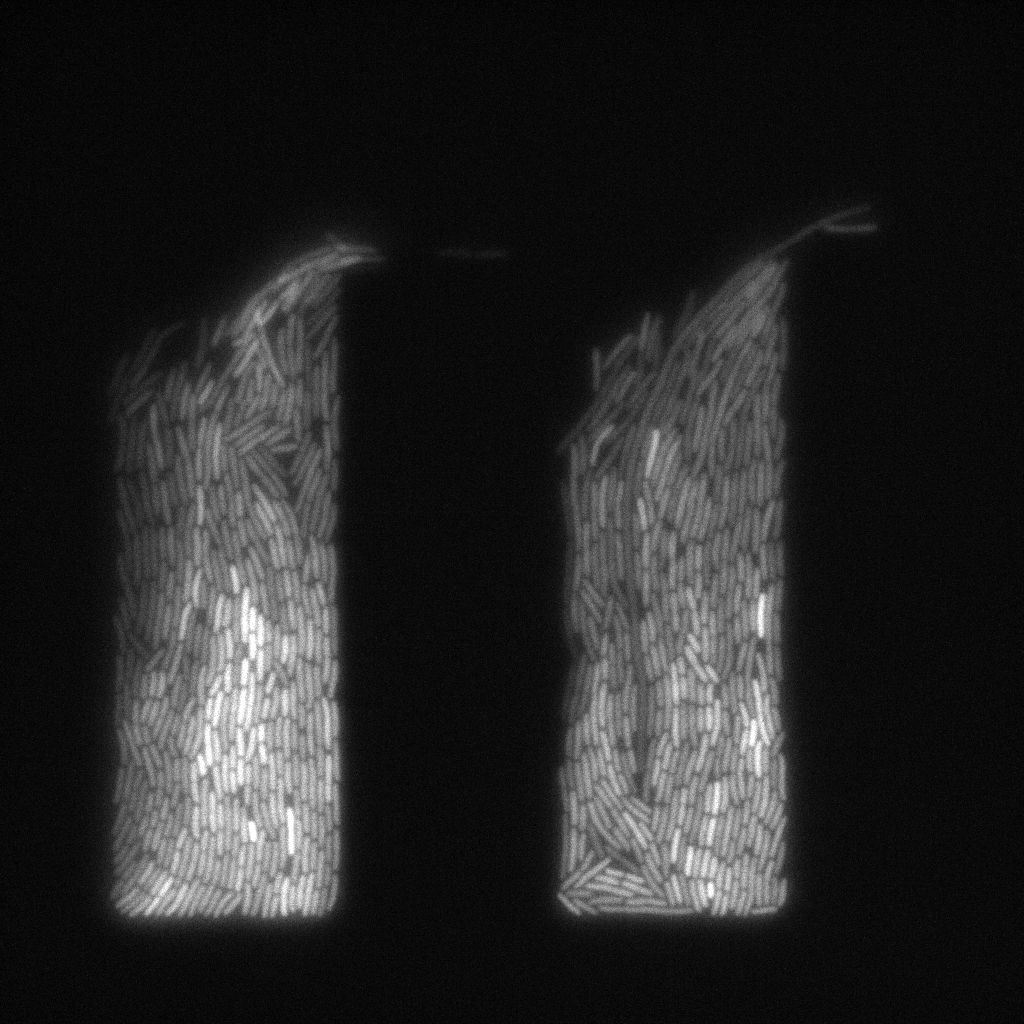

Supplement: Supplementary file 5 — Source Data for Figure 2 [file MSB-18-e10680-s004.zip › Fig2 panel 2a/Source images from the microscope/fluorescence unprocessed/pos2-y-2.tif]

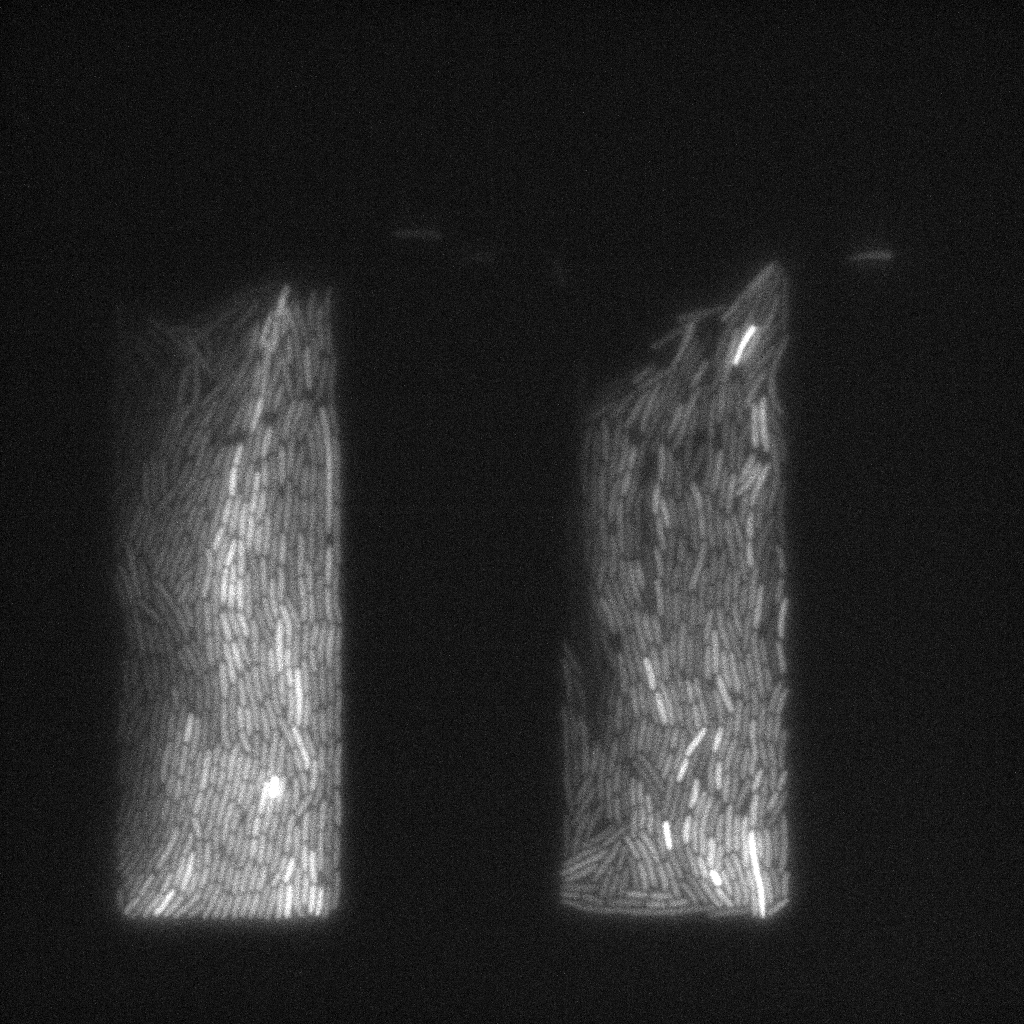

Supplement: Supplementary file 5 — Source Data for Figure 2 [file MSB-18-e10680-s004.zip › Fig2 panel 2a/Source images from the microscope/fluorescence unprocessed/pos2-y-3.tif]

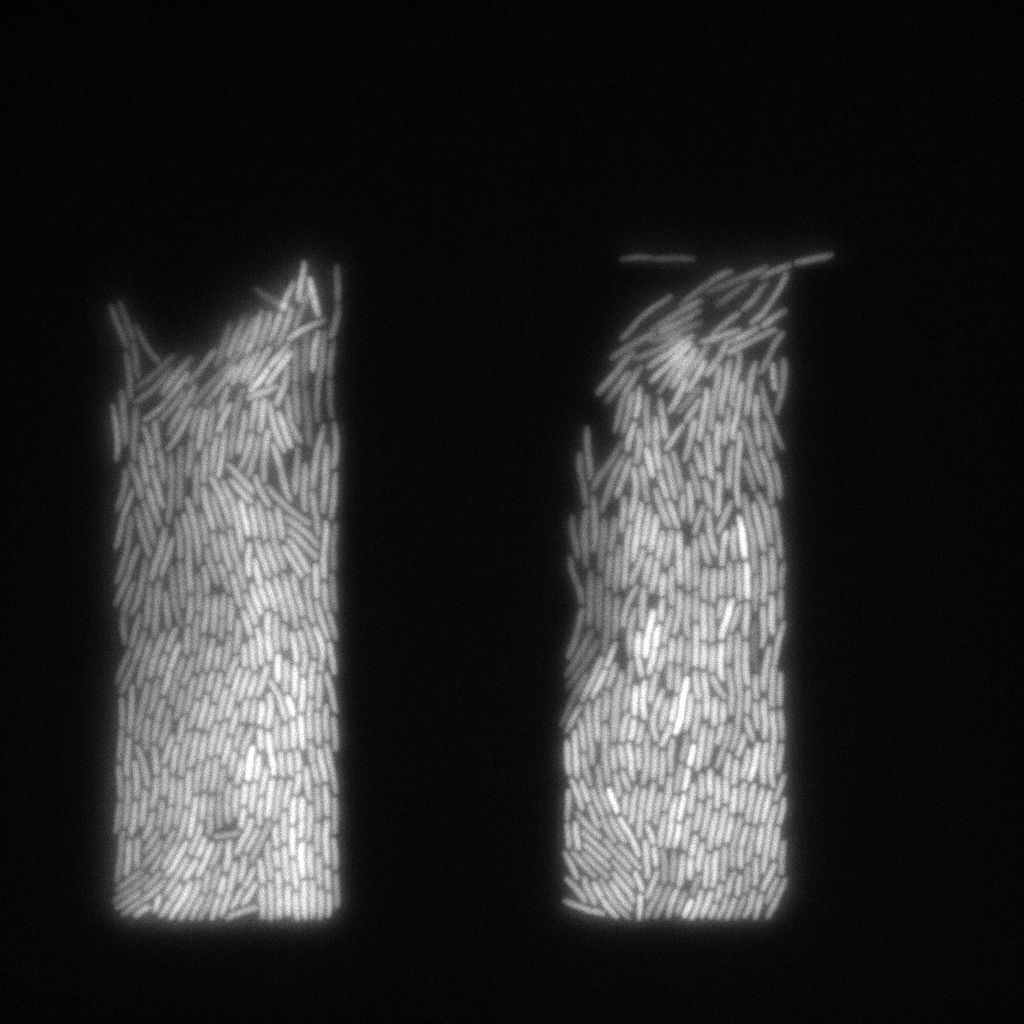

Supplement: Supplementary file 5 — Source Data for Figure 2 [file MSB-18-e10680-s004.zip › Fig2 panel 2a/Source images from the microscope/fluorescence unprocessed/pos2-y-1.tif]

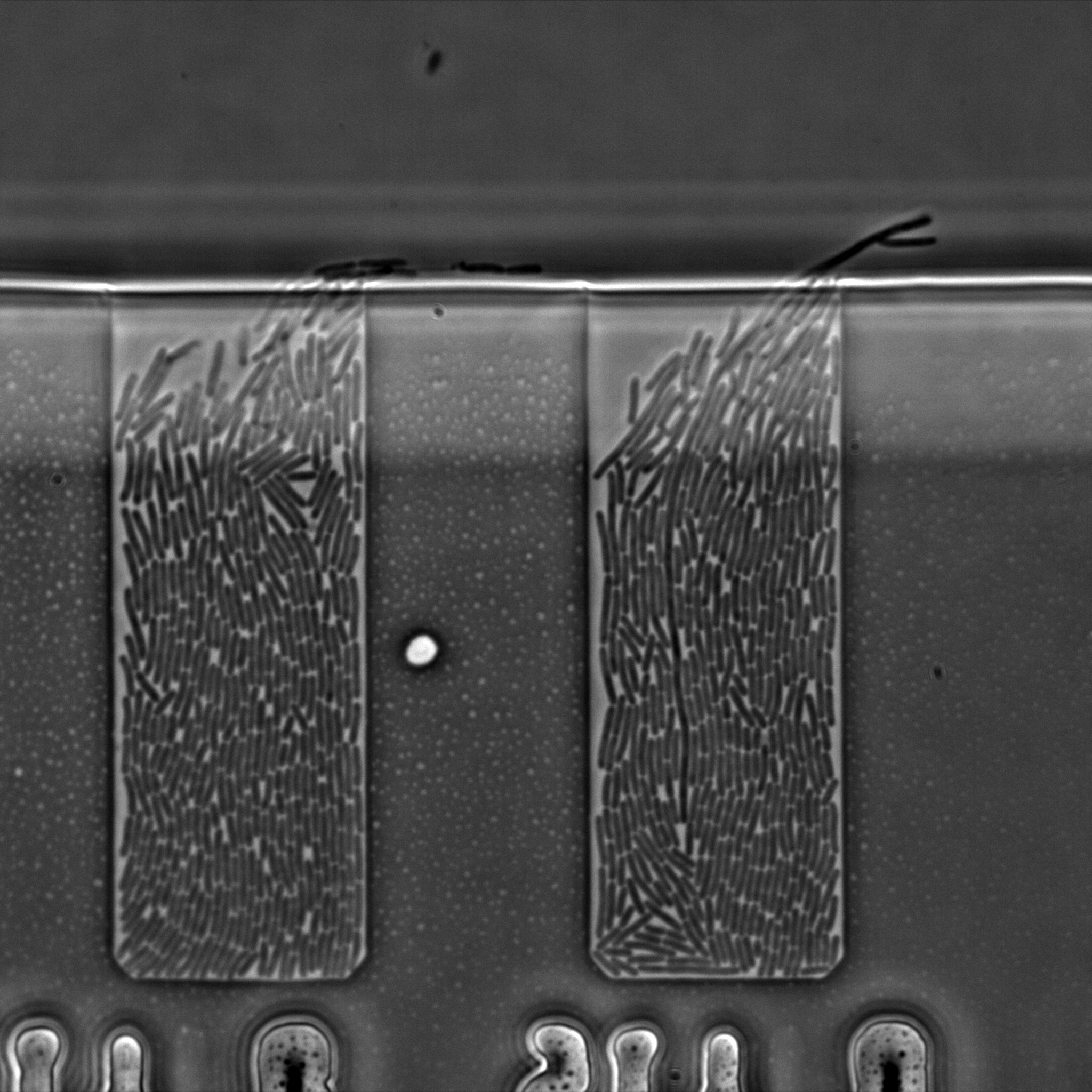

Supplement: Supplementary file 5 — Source Data for Figure 2 [file MSB-18-e10680-s004.zip › Fig2 panel 2a/Source images from the microscope/brightfield unprocessed/pos2-p-2-2.tif]

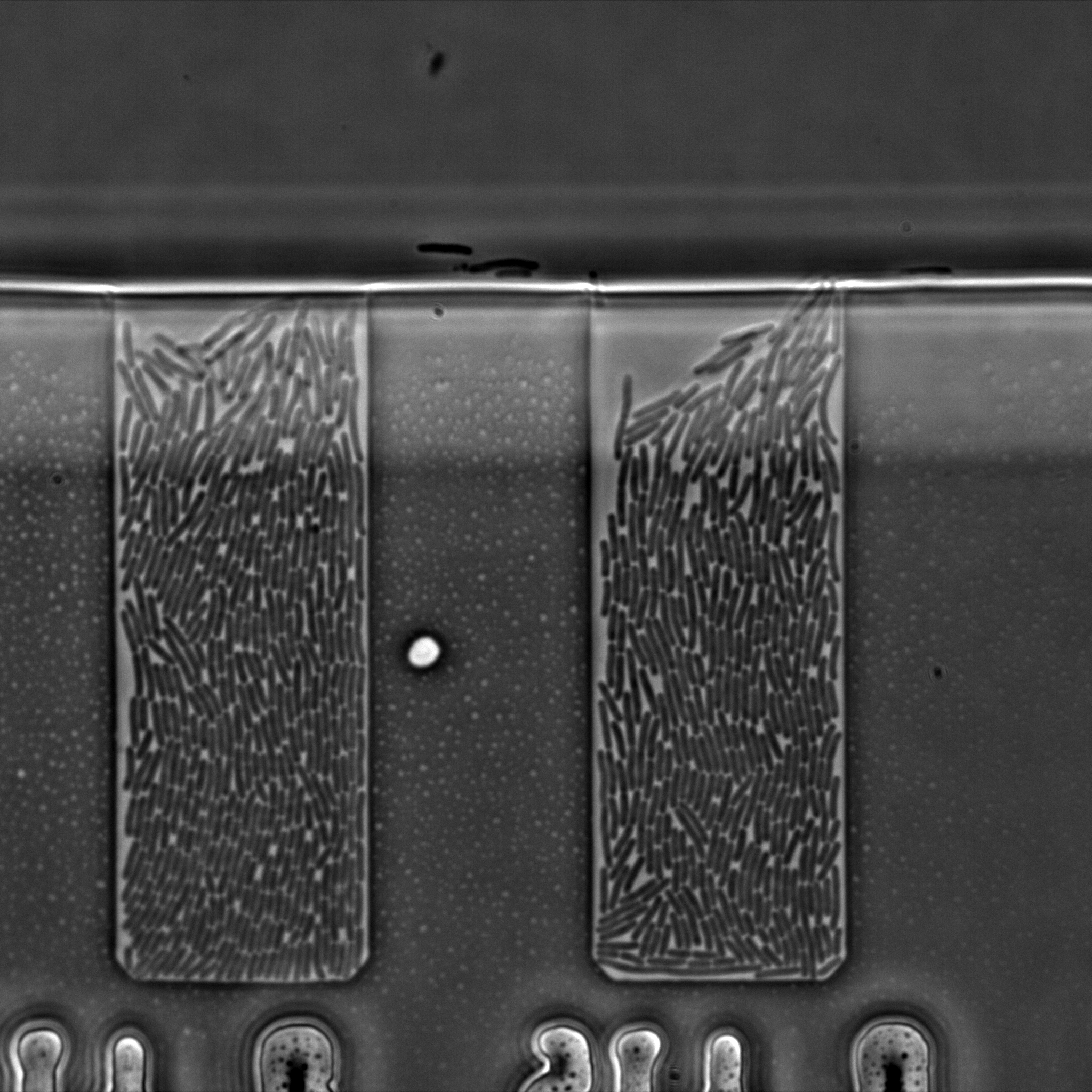

Supplement: Supplementary file 5 — Source Data for Figure 2 [file MSB-18-e10680-s004.zip › Fig2 panel 2a/Source images from the microscope/brightfield unprocessed/pos2-p-2-3.tif]

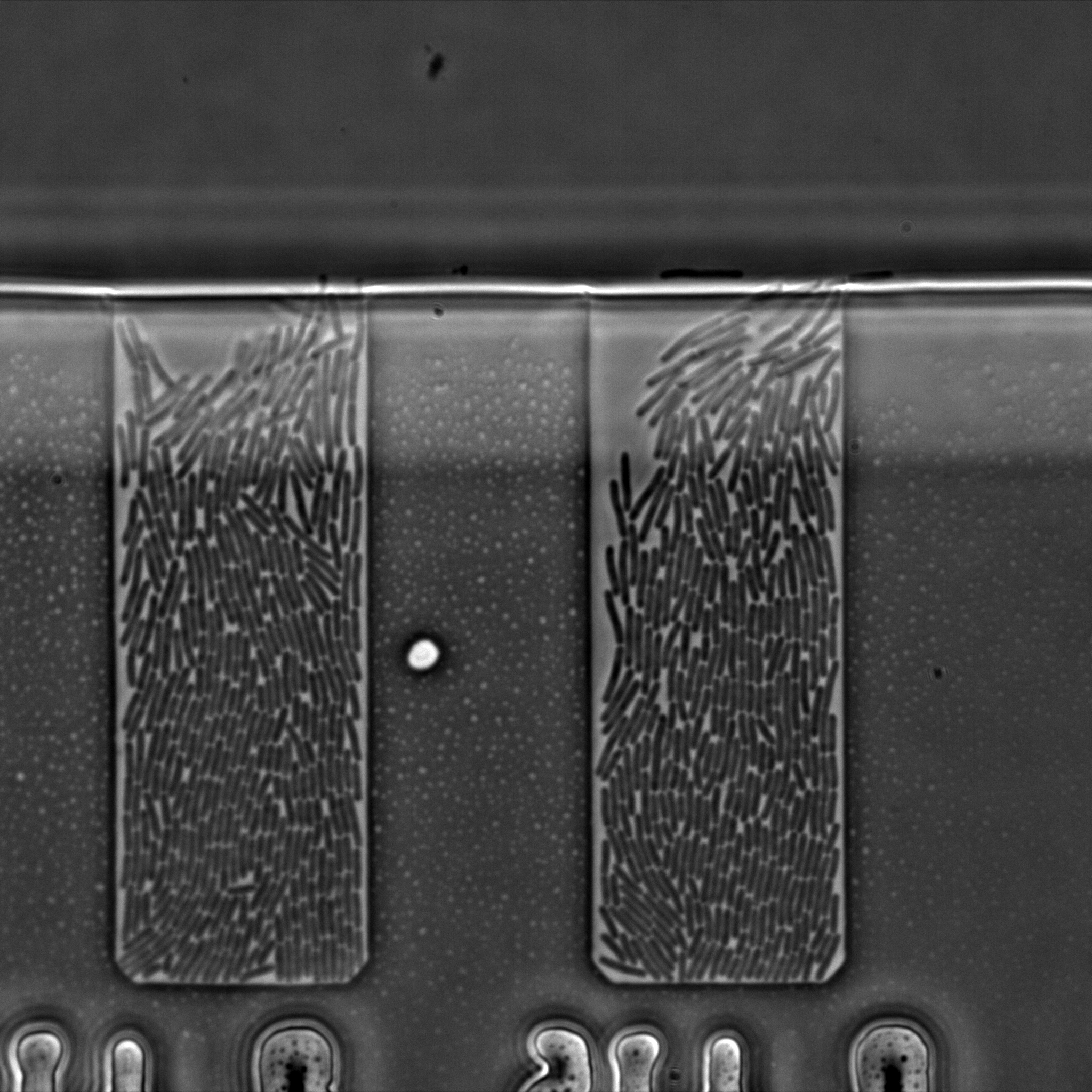

Supplement: Supplementary file 5 — Source Data for Figure 2 [file MSB-18-e10680-s004.zip › Fig2 panel 2a/Source images from the microscope/brightfield unprocessed/pos2-p-2-1.tif]

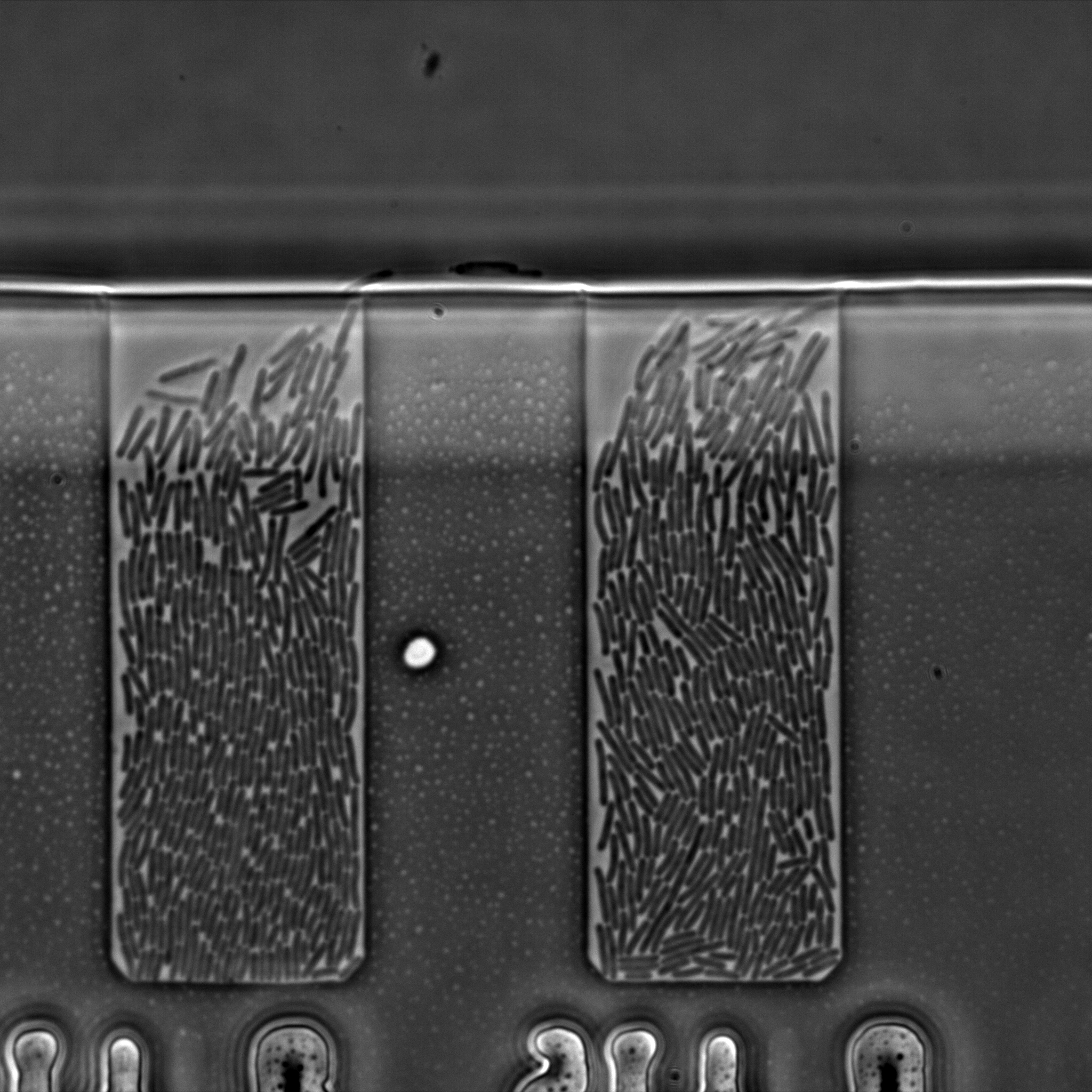

Supplement: Supplementary file 5 — Source Data for Figure 2 [file MSB-18-e10680-s004.zip › Fig2 panel 2a/Source images from the microscope/brightfield unprocessed/pos2-p-2-4.tif]
