# Supplementary material for: Single cell variability of CRISPR‐Cas interference and adaptation
Source: Mol Syst Biol. 2022 Apr 25;18(4):e10680. doi: 10.15252/msb.202110680 (PMC10561596; doi:10.15252/msb.202110680)
Supplement: Supplementary file 6 [file MSB-18-e10680-s006.pdf]

molecular systems biology

Single Cell Variability of CRISPR-Cas Interference and Adaptation

Rebecca McKenzie, Emma Keizer, Jochem Vink, Jasper van Lopik, Ferhat Büke, Vera Kalkman, Christian Fleck, Sander Tans, and Stan Brouns
DOI: 10.15252/msb.202110680

Corresponding author(s): Stan Brouns (stanbrouns@gmail.com) , Sander Tans (tans@amolf.nl), Christian Fleck (christian.fleck@fdm.uni-freiburg.de)

Review Timeline:

Submission Date:	8th Sep 21
Editorial Decision:	18th Oct 21
Revision Received:	4th Feb 22
Editorial Decision:	3rd Mar 22
Revision Received:	29th Mar 22
Accepted:	31st Mar 22

Editor: Jingyi Hou

Transaction Report:

(Note: With the exception of the correction of typographical or spelling errors that could be a source of ambiguity, letters and reports are not edited. Depending on transfer agreements, referee reports obtained elsewhere may or may not be included in this compilation. Referee reports are anonymous unless the Referee chooses to sign their reports.)

18th Oct 2021

Manuscript Number: MSB-2021-10680
Title: Single Cell Variability of CRISPR-Cas Interference and Adaptation
Author: Rebecca McKenzie
Emma Keizer
Jochem Vink
Jasper van Lopik
Ferhat Büke
Vera Kalkman
Christian Fleck
Sander Tans
Stan Brouns

Dear Dr. Brouns,

Thank you for submitting your work to Molecular Systems Biology. We have now heard back from the three reviewers who agreed to evaluate your manuscript. As you will see from the reports below, the reviewers acknowledge the potential interest of the study. They raise however a series of concerns, which we would ask you to address in a major revision.

Since the reviewers' recommendations are relatively straightforward, there is no need to reiterate all the points listed below. Importantly, as both Reviewers #2 and #3 mentioned, some level of experimental validation would strengthen and better support the conclusions from the model.

All other issues raised by the reviewers need to be satisfactorily addressed as well. As you may already know, our editorial policy allows in principle a single round of major revision and it is therefore essential to provide responses to the reviewers' comments that are as complete as possible.

On a more editorial level, we would ask you to address the following issues:

- Please provide a .docx formatted version of the manuscript text (including legends for main figures, EV figures and tables). Please make sure that the changes are highlighted to be clearly visible.

- Please provide individual production quality figure files as .eps, .tif, .jpg (one file per figure).

-Please provide a .docx formatted letter INCLUDING the reviewers' reports and your detailed point-by-point responses to their comments. As part of the EMBO Press transparent editorial process, the point-by-point response is part of the Review Process File (RPF), which will be published alongside your paper.

-Please note that all corresponding authors are required to supply an ORCID ID for their name upon submission of a revised manuscript.

-We replaced Supplementary Information with Expanded View (EV) Figures and Tables that are collapsible/expandable online (see examples in http://msb.embopress.org/content/11/6/812). A maximum of 5 EV Figures can be typeset. EV Figures should be cited as 'Figure EV1, Figure EV2" etc... in the text and their respective legends should be included in the main text after the legends of regular figures.

Additional Tables/Datasets should be labeled and referred to as Table EV1, Dataset EV1, etc. Legends have to be provided in a separate tab in case of .xls files. Alternatively, the legend can be supplied as a separate text file (README) and zipped together with the Table/Dataset file.

For the figures and tables that you do NOT wish to display as Expanded View figures, they should be bundled together with their legends in a single PDF file called *Appendix*, which should start with a short Table of Content. Each legend should be below the corresponding Figure/Table in the Appendix. Appendix figures and tables should be referred to in the main text as: "Appendix Figure S1, Appendix Figure S2, Appendix Table S1" etc. See detailed instructions regarding expanded view here: https://www.embopress.org/page/journal/17444292/authorguide#expandedview.

-Before submitting your revision, primary datasets (and computer code, where appropriate) produced in this study need to be deposited in an appropriate public database (see https://www.embopress.org/page/journal/17444292/authorguide#dataavailability).

Please remember to provide a reviewer password if the datasets are not yet public.

The accession numbers and database should be listed in a formal "Data Availability" section (placed after Materials & Method) that follows the model below (see also https://www.embopress.org/page/journal/17444292/authorguide#dataavailability). Please note that the Data Availability Section is restricted to new primary data that are part of this study.

Data availability

The datasets (and computer code) produced in this study are available in the following databases:

- RNA-Seq data: Gene Expression Omnibus GSE46843 (https://www.ncbi.nlm.nih.gov/geo/query/acc.cgi?acc=GSE46843)
- [data type]: [name of the resource] [accession number/identifier/doi] ([URL or identifiers.org/DATABASE:ACCESSION])

*** Note - All links should resolve to a page where the data can be accessed. ***

- We would encourage you to include the source data for figure panels that show essential quantitative information. Additional information on source data and instruction on how to label the files are available at < https://www.embopress.org/page/journal/17444292/authorguide#sourcedata >.

- All Materials and Methods need to be described in the main text. We would encourage you to use 'Structured Methods', our new Materials and Methods format. According to this format, the Material and Methods section should include a Reagents and Tools Table (listing key reagents, experimental models, software and relevant equipment and including their sources and relevant identifiers) followed by a Methods and Protocols section in which we encourage the authors to describe their methods using a step-by-step protocol format with bullet points, to facilitate the adoption of the methodologies across labs. More information on how to adhere to this format as well as downloadable templates (.doc or .xls) for the Reagents and Tools Table can be found in our author guidelines: < https://www.embopress.org/page/journal/17444292/authorguide#researcharticleguide >. An example of a Method paper with Structured Methods can be found here: .

-Regarding data quantification:
Please ensure to specify the name of the statistical test used to generate error bars and P values, the number (n) of independent experiments (please specify technical or biological replicates) underlying each data point and the test used to calculate p-values in each figure legend. Discussion of statistical methodology can be reported in the materials and methods section, but figure legends should contain a basic description of n, P and the test applied.
Graphs must include a description of the bars and the error bars (s.d., s.e.m.).
Please also include scale bars in all microscopy images.

- Please provide a "standfirst text" summarizing the study in one or two sentences (approximately 250 characters, including space), three to four "bullet points" highlighting the main findings and a "synopsis image" (550px width and 400-600px height, PNG format) to highlight the paper on our homepage.
Here are a couple of examples:
https://www.embopress.org/doi/10.15252/msb.20199356
https://www.embopress.org/doi/10.15252/msb.20209475
https://www.embopress.org/doi/10.15252/msb.209495

When you resubmit your manuscript, please download our CHECKLIST (http://bit.ly/EMBOPressAuthorChecklist) and include the completed form in your submission.
Please note that the Author Checklist will be published alongside the paper as part of the transparent process (https://www.embopress.org/page/journal/17444292/authorguide#transparentprocess).

If you feel you can satisfactorily deal with these points and those listed by the referees, you may wish to submit a revised version of your manuscript. Please attach a covering letter giving details of the way in which you have handled each of the points raised by the referees. A revised manuscript will be once again subject to review and you probably understand that we can give you no guarantee at this stage that the eventual outcome will be favorable.

I look forward to receiving your revised manuscript soon.

Kind regards,
Jingyi

Jingyi Hou
Editor

If you do choose to resubmit, please click on the link below to submit the revision online *within 90 days*.

https://msb.msubmit.net/cgi-bin/main.plex

IMPORTANT: When you send your revision, we will require the following items:
1. the manuscript text in LaTeX, RTF or MS Word format
2. a letter with a detailed description of the changes made in response to the referees. Please specify clearly the exact places in the text (pages and paragraphs) where each change has been made in response to each specific comment given
3. three to four 'bullet points' highlighting the main findings of your study
4. a short 'blurb' text summarizing in two sentences the study (max. 250 characters)
5. a 'thumbnail image' (550px width and max 400px height, Illustrator, PowerPoint or jpeg format), which can be used as 'visual title' for the synopsis section of your paper.
6. Please include an author contributions statement after the Acknowledgements section (see https://www.embopress.org/page/journal/17444292/authorguide)
7. Please complete the CHECKLIST available at (https://bit.ly/EMBOPressAuthorChecklist).
Please note that the Author Checklist will be published alongside the paper as part of the transparent process (https://www.embopress.org/page/journal/17444292/authorguide#transparentprocess).
8. Please note that corresponding authors are required to supply an ORCID ID for their name upon submission of a revised manuscript (EMBO Press signed a joint statement to encourage ORCID adoption).
(https://www.embopress.org/page/journal/17444292/authorguide#editorialprocess)
9. When assembling figures, please refer to our figure preparation guideline in order to ensure proper formatting and readability in print as well as on screen:
https://bit.ly/EMBOPressFigurePreparationGuideline
See also figure legend guidelines: https://www.embopress.org/page/journal/17444292/authorguide#figureformat

Currently, our records indicate that the ORCID for your account is 0000-0002-9573-1724.

Please click the link below to modify this ORCID:
Link Not Available

The system will prompt you to fill in your funding and payment information. This will allow Wiley to send you a quote for the article processing charge (APC) in case of acceptance. This quote takes into account any reduction or fee waivers that you may be eligible for. Authors do not need to pay any fees before their manuscript is accepted and transferred to the publisher.

As a matter of course, please make sure that you have correctly followed the instructions for authors as given on the submission website.

*** PLEASE NOTE *** As part of the EMBO Press transparent editorial process initiative (see our Editorial at https://dx.doi.org/10.1038/msb.2010.72), Molecular Systems Biology publishes online a Review Process File with each accepted manuscripts. This file will be published in conjunction with your paper and will include the anonymous referee reports, your point-by-point response and all pertinent correspondence relating to the manuscript. If you do NOT want this File to be published, please inform the editorial office at msb@embo.org within 14 days upon receipt of the present letter.

Reviewer #1:

McKenzie and Keizer et al. present a study of CRISPR-Cas interference and adaptation at the single cell level. This is one big step further in mechanistic studies as previous studies were done at the ensemble level. As the spacer adaptation is done rather sporadically, ensemble studies lack the temporal resolution to resolve mechanistic steps. The time lapse microscope and microfluidic device allows the authors to trace the cell lineages. From individual lineages and genealogical relations, they found that the direct interference occurs quickly and consistently, however priming is highly variable and much slower. It is impressive that the authors was able to show that the Cascade copy number in cells and demonstrate its correlation with the interference rate. Although the conclusion is within expectation, the ability of the experimental setup to resolve such mechanism at the single cell level is very impressive. More exciting discoveries will emerge from this pipeline.

Overall, this is very interesting paper that moves the field of Type I inference and adaptation forward. The technological and conceptual innovation are obvious. The result interpretation is rigorous, and the writing is quite polished. This reviewer Has only

a few minor suggestions.

1. In figure 4b, the Y-axis unit isμm-2. This reviewer finds it difficult to appreciate what this unit means. Does it make sense to convert the unit to "per cell".

2. Line 673, need a "." after "... via priming"

Reviewer #2:

In this work, McKenzie and coworkers investigated the single-cell dynamics of CRISPR-based interference and primed acquisition. Using the I-E system in E. coli as a model, the authors created single-cell movies tracking dividing cells over time, using a fluorescent protein encoded on a targeted plasmid as a readout of interference. They showed that direct interference was a fast and uniform process, whereas primed interference was much noisier and associated with either fast or slow timescales. They also noted correlations between cell division time and direct and primed interference as well as the importance of ancestry on interference and especially priming. By measuring the fluorescence of mCherry fused to Cas8e, they found that direct interference was associated with higher Cascade concentrations. Finally, the authors applied a stochastic model that, upon parameter fitting, reproduced the same general trends as the experimental results. The model also predicted that target search rather than spacer acquisition accounted for the variability associated with primed interference.

Overall, this work represents the first effort to my knowledge to investigate CRISPR immunity at the single-cell level, with implications for bacterial-phage dynamics and evolution. The authors had to devise new means of quantifying interference on top of the challenges of assessing movies with lineage tracking. I thought the work was thorough and generally well described and should be well recognized by the field, even if there are some aspects that need improvement or with better caveats.

Major comments:

1. I'm not convinced the box plots of percentile rankings are a reasonable way to evaluate different factors on direct and primed interference. Comparisons are made between two populations (clearance and no clearance), yet all values are shown. These two should be broken apart so it's clear what the two distributions look like. In addition, significance is determined using a binomial distribution, although this assumes a normally distributed dataset that does not appear to be the case here.

2. A necessary but notable caveat of the work is the use of an inducible promoter to drive Cas operon expression. These promoters create a somewhat artificial setup where Cascade is being expressed and forming while the invader is present in the cell. The use of inducers (one metabolizable) with associated uptake and catabolic pathways could introduce fluctuations not associated with constitutive promoters. I fully recognize that a constitutive promoter couldn't be used under this experimental setup, although the use of an inducible promoter does limit how broadly conclusions can be drawn about the dynamics of the experimental system.

3. The authors draw some major conclusions purely from the stochastic modeling, including the contribution of target search to the variability in primed interference as well as the role of copy number. The conclusions are stated rather definitely, although there are many reasons why the model could be misleading (e.g., model assumptions, oversimplification, important but missing mechanistic steps, fit parameters in the wrong regime). Any conclusions from the model need to be tempered or supported with experimental evidence. For instance, changing the origin-of-replication to a higher-copy number and repeating some basic experiments could support one of these major conclusions.

Other comments:

4. L. 51-52: The PAMs recognized by the I-E system are far broader than CTT. Slight rewording here could convey (or at least not contradict) this point.

5. L. 64, 331: I don't think tug-of-war is the right analogy, as it applies to similar entities trying to undermine each other. Instead, the analogy of a race would be more appropriate, where which proceeds faster determines the final outcome (invader propagation or clearance).

6. L. 139: this statement isn't quite true, as CRISPR-Cas was present under both experimental conditions. Instead, you could claim that the effect was target-dependent. To claim CRISPR-Cas dependence, either would have to be removed while keeping the target present.

7. L. 142: avoid using "significant" unless statistical comparisons are being made. "substantial" is easy to use instead.

8. L. 146: I wouldn't say YFP is diluted exponentially. It's more that YFP is diluted in half with each cell division, so halting YFP

production results in the apparent YFP levels to drop exponentially in individual cells over time.

9. L. 174-175: I'm having a hard time understanding what this sentence is conveying.

10. L. 177-178: could the authors sequence a small number of acquired spacers to show that the associated protospacers are all flanked by the expected CTT PAM? I know this is expected, although the current sentence sets up this experiment.

11. L. 201-203: I had trouble getting through this sentence as well.

12. L. 204: are the fractions equal?

13. L. 212-213: provide some explanation (or a reference) why ranking analysis suppresses long-term trends.

14. L. 230-232: causality is questionable here, as engaging acquisition could be slowing growth.

15. L. 251: provide a p-value similar to the prior sentence.

16. L. 255: explain what Cascade search hours are, as this term hasn't been introduced and is not clearly linked to the analyses in this section.

17. L. 258-259: I'm having trouble understanding how the correlation increases in magnitude but is not significant. Some rewording would be helpful here.

18. L. 275-276: was the reduction in binding affinity through parameter fitting? If so, this should be clearly stated rather than generally saying the binding affinity was found to be lower.

19. L. 344-346: alternatively, cells with fewer plasmids have a lower resource burden and may divide more quickly.

20. L. 375-377: I'm not sold on this line of argumentation. If anything, a highly-susceptible subpopulation could greatly increase phage titers, even if other cells are more efficiently clearing phages that do infect.

21. Figure 1C: include the blue segment of the target plasmid on the left. Otherwise, it looks like the plasmid is changing (versus CRISPR picking up a new spacer that can target a different location in the plasmid).

22. Figure 2C: Change CT to a different color, unless the authors intend for it to be linked to fluorescence concentration.

23. Figure 3 legend: provide more descriptions, such as the important labels in panel a, what the black dots represent in panel b, or what's meant by loss of cells.

24. Figure 3: what's the difference between growth rate and interdivision time? I would assume that one is merely the inverse of the other.

25. Figure 4 title: this appears incorrect. It's a rephrased version of the Figure 3 title and doesn't address Cascade levels.

26. Figure 4b: there should be a unit in the numerator (e.g., molecules).

27. Figure 5: add a schematic depicting the key steps of the model. That way the reader can have some understanding of how direct and primed interference are captured without having to dig deeply into the SI methods.

Reviewer #3:

Summary
McKenzie et al. establish an elegant system to monitor the activity of the E. coli type I CRISPR-Cas system in live single cells. The authors utilize two experimental setups to study interference and primed acquisition and find that while interference occurs on a similar timeframe in most cells, the timing of primed acquisition is highly variable. Most intriguingly slow-growing cells are better able to conduct priming. The authors use modeling to hypothesize that priming stochasticity owes to low-affinity binding of Cascade to target DNA and that stochastic CRISPR-Cas expression can accelerate priming.

General remarks
The microscopy and analysis is rigorously conducted allowing the authors to gain new insights into the single-cell biology of CRISPR-Cas defense. In the second half of the paper, the authors use modeling to generate several hypotheses regarding primed adaptation, and the paper would benefit significantly from experiments that test these hypotheses. The paper is presented as the first of its kind, however, at least one other study has used time-lapse microscopy to study the dynamics and

variability of type I CRISPR-Cas defense (Guan, Quant Biol, 2017). This work should be cited and discussed in the authors introduction and discussion, both in terms of background/context and in how the authors methodologies and results compare. Nonetheless, there are several technical and conceptual advances, including the use of an inducible CRISPR-Cas system to monitor plasmid loss and the relationship between CRISPR-Cas activites and cellular properties, such as growth rate. This work will be of interest to the CRISPR-Cas field, bacterial cell biologists and microbiologists interested in phage defense.

Major points

As stated above, this paper would be strengthened by experimental validation of the hypotheses generated by modeling:
- Binding affinity drives priming variability - can the authors test alternative PAM/seed escaper mutants with distinct target affinities? Even in the absence of known affinities, testing a seed escaper as an alternative to the PAM mutant already tested would add depth to the paper.
- Target abundance affects the balance between primed adaptation and primed interference - can the authors test plasmids with different copy numbers and/or different numbers of targets on the same plasmid?

The observation that slow cell growth is associated with cells that will ultimately undergo primed adaptation is fascinating. However, I am not sure that this conclusion is fully supported by the data.
- The major question is whether slow cell growth truly precedes adaptation and describes a cellular state which is more predisposed to adaptation or whether the act of adaptation is itself slowing cells down, and this is bleeding into the growth rate calculation. One may assume that adaptation could slow cells down due to broken DNA (substrates for adaptation) causing a DNA damage response. The authors use a lookback window of 90-60 minutes before PLT because adaptation occurs on average 60 minutes before plasmid loss. However, if this is the average, then many individual cells will be undergoing adaptation >60 mins before PLT which will be during and not after the window. Wouldn't it be better to set the lookback window on a cell-by-cell basis tied to the moment that fluorescence begins to decrease, which is an event more proximal (and therefore less variable) to adaptation than PLT? If this is not possible, how can the authors be sure that they are measuring events occurring before and not during spacer acquisition?
- If the authors are instead suggesting that slow growth is caused by the act of spacer acquisition (or substrate generation) and is therefore not indicative of a cellular state that is predisposed to undergo priming, this is a less interesting but still novel conclusion. Regardless, the authors should more thoroughly distinguish between these models (slow growth before priming vs. slow growth during priming) and discuss which precisely they are able to test.
- In 3F, how can the cells show a reduced growth rate with an unchanged size and division time? If cells are growing more slowly but maintaining the same size, doesn't this mean that they must be also dividing more slowly (i.e. have a longer interdivision time?)
- In Sup Fig. 17, it is mentioned that slow cell growth is associated with higher Cascade concentrations. Could this be why slow growth is associated with priming? Can you control for Cascade expression and determine if slow cell growth is still associated with priming?
- The discussion should elaborate on why the authors believe slow growth is associated with priming. There are several possibilities (DNA damage, some other stress, high Cascade concentrations, something else) and the authors should speculate on what exactly they think is going on. Line 438 "perhaps due to higher target DNA availability" may allude to the DNA damage hypothesis, but it unclear to me what exactly is meant as written.
-

Minor points

End of intro (99-103): mention that these results were based on modeling, as written they sound like experimental conclusions.

206-207: I'm struggling to understand why the moment of loss wouldn't be more biased toward earlier stages of the cell cycle when the adaptation even occurred in the parent - causing both sisters to lose the plasmid.

Fig. 3F: Why doesn't the data in 3F (30 minute lookback window) match the data in Sup Fig. 9 (top left)? Is this different data? Does the "30 minute lookback" in the 3F legend refer to something other than wstart = 30 mins and wend = 0 min? If the data in 3F refers to wstart = 90 mins and wend = 60 mins (my guess from line 227), then this should be stated as such in the legend.

216: I would prefer that the lookback window is described in some more detail here instead of saying "determined using autocorrelation times" which won't be meaningful to many readers. Can you explain what lookback window was used for Fig. 3F and why?

It is unclear to me how growth rate was calculated. This should be described somewhere in the methods, unless I missed it.

247: Figure legend says 30 min lookback window, text says 60 min lookback window

252-253: Doesn't this statement need to refer to a figure or paper?

255-259: I tried to read this several times and cannot follow. Can you expand this section in order to clarify the methods and

interpretations? How are Cascade search hours calculated? Can you add a few "suggesting that..." phrases to really hammer home what the data is telling you?

283: "by a factor of 100"

284: find > found

Final section 310-324: I had to read this section several times. What was motivation for changing the variation of Cascade expression? Is this relevant to some natural aspect of Cascade expression? The last sentence is a bit anti-climactic and refers elsewhere for a more thorough explanation. Can this be expanded upon in the results section, specifically "the highly non-linear fashion"? Is there a figure that can be pointed to to demonstrate this?

497: 'was performed 1ul' > 'was performed with 1 ul'

623: 'resulting in' > 'results in'

624: 'To invoke priming the' > 'To invoke priming, the'

631: 'To allow long term imaging cells' > 'To allow long term imaging, cells'

672/673: 'Depict clearance' > 'Clearance'

762: 'integrated upstream of the cas8e subunit' > 'fused to the N terminus of the Cas8e subunit'

FIGURES

Fig 1 - Panels a & c: Label the promoters, or describe them in the figure legend, so we know which are native/inducible (and arabinose vs IPTG inducible) without checking the methods section. Also, Cas3 and Cas1, 2 in locus diagram should be lower case/italicized.

Fig 2D/G - Label the x axes 'PLT' instead of 'Time.' This would keep the figure consistent with panel descriptions, and distinguish these graphs from those in panels b & f (where they use 'Time' to refer to time after induction, rather than PLT). Another minor note, but I kind of want the y axes scale (percentage) to be the same in both graphs to allow for direct comparison.

Fig 2F: Why does fluorescence decrease sharply at the beginning? Is that due to dilution following cell divisions? Photobleaching of initial YFP population?

Fig 3A: Describe the difference between open and closed circles. I think closed circles are cells in which plasmid the was lost (if same as closed circles in b), but it would be nice if they spelled that out.

Fig 3B: Again, would like x axis labeled PLT. Also, it's not intuitive what exactly what is being shown and it is not described in the legend.

Fig 3C - priming: are the colors swapped?

Sup Fig 2 legend - consider mentioning that this is for a representative individual lineage?

Sup Fig 4 title: 'during Direct interference' > 'during direct interference'

Sup Fig 6 description: What is meant by 'the moment of detection' (filled red dots)?

Sup Fig 5 - Can you put the lane information above each lane instead of referring readers to the legend to see what each lane is?

Sup Fig 6 - How does this compare to a control in which there is no interference? Is there 0% plasmid loss in this case? Also, the fluorescence curves look like they're on a sharp decline even before the plasmid loss event. Shouldn't the loss be more gradual given that each cleavage event (as the copy number decreases) is significantly impaired relative to interference with a perfect PAM? In other words, how does the rate of fluorescence decrease in those rare lineages shown compare to those during perfect interference?

Response to reviewers

We thank all three reviewers for careful reading and helpful comments. Below we provide a detailed response to each of the comments.

Reviewer #1:

McKenzie and Keizer et al. present a study of CRISPR-Cas interference and adaptation at the single cell level. This is one big step further in mechanistic studies as previous studies were done at the ensemble level. As the spacer adaptation is done rather sporadically, ensemble studies lack the temporal resolution to resolve mechanistic steps. The time lapse microscope and microfluidic device allows the authors to trace the cell lineages. From individual lineages and genealogical relations, they found that the direct interference occurs quickly and consistently, however priming is highly variable and much slower. It is impressive that the authors was able to show that the Cascade copy number in cells and demonstrate its correlation with the interference rate. Although the conclusion is within expectation, the ability of the experimental setup to resolve such mechanism at the single cell level is very impressive. More exciting discoveries will emerge from this pipeline.

Overall, this is very interesting paper that moves the field of Type I inference and adaptation forward. The technological and conceptual innovation are obvious. The result interpretation is rigorous, and the writing is quite polished. This reviewer Has only a few minor suggestions.

We are grateful and humbled by the response from the reviewer. We have addressed the minor comments, as below.

1. In figure 4b, the Y-axis unit is µm-2. This reviewer finds it difficult to appreciate what this unit means. Does it make sense to convert the unit to "per cell".

We thank the reviewer for this suggestion. The unit per cell is not ideal to employ due to the large variation seen in cell size during growth. To address this point the authors have adapted the y-axis label description to Cascade molecules / μm^2, and modified the figure caption to clarify the significance of this unit in terms of cell area. The legend now reads “**B** Cascade concentration of single-cell lineages over time from induction. The surface area of E. coli cells in our experiments is approximately 2 μm^2, resulting in an estimate of approximately 500 molecules of Cascade per cell at steady state.”

2. Line 673, need a "." after "... via priming"

The authors thank the reviewer for pointing out this mistake. We have changed it.

Reviewer #2:

In this work, McKenzie and coworkers investigated the single-cell dynamics of CRISPR-based interference and primed acquisition. Using the I-E system in E. coli as a model, the authors created single-cell movies tracking dividing cells over time, using a fluorescent protein encoded on a targeted plasmid as a readout of interference. They showed that direct interference was a fast and uniform process, whereas primed interference was much noisier and associated with either fast or slow timescales. They also noted correlations between cell division time and direct and primed interference as well as the importance of ancestry on interference and especially priming. By measuring the fluorescence of mCherry fused to Cas8e, they found that direct interference was associated with higher Cascade concentrations. Finally, the authors applied a stochastic model that, upon parameter fitting, reproduced the same general trends as the experimental results. The model also predicted that target search rather than spacer acquisition accounted for the variability associated with primed interference.

Overall, this work represents the first effort to my knowledge to investigate CRISPR immunity at the single-cell level, with implications for bacterial-phage dynamics and evolution. The authors had to devise new means of quantifying interference on top of the challenges of assessing movies with lineage tracking. I thought the work was thorough and generally well described and should be well recognized by the field, even if there are some aspects that need improvement or with better caveats.

We would like to thank the reviewer for the positive comments and important questions raised that have helped us to increase the quality of our work. Below we give a point-by-point answer to each question.

Major comments:

1. I'm not convinced the box plots of percentile rankings are a reasonable way to evaluate different factors on direct and primed interference. Comparisons are made between two populations (clearance and no clearance), yet all values are shown. These two should be broken apart so it's clear what the two distributions look like. In addition, significance is determined using a binomial distribution, although this assumes a normally distributed dataset that does not appear to be the case here.

We thank the reviewer for bringing up these points. First, they indicated to us that our justification for basing our analysis on rank, rather than a phenotypic parameter in a quantitative manner, was not sufficiently clear. We believe the rank-based analysis approach is valid and necessary due to the non-stationary nature of the cellular growth rate over time, and the need to analyze cells at different points in time. A regular correlation analysis, in which one would compare features of loss-lineages that cleared the plasmids at different times over the course of the 36 hour experiment, can erroneously detect trends, which are not related to the CRISPR-Cas response. Second, to clarify, our analysis does not compare two populations (clearance and no clearance). For the reasons discussed above and others, this is also not advisable. Rather, it ranks a single loss-lineage (a single cell

lineage where the plasmid is lost) among all other lineages that do retain their plasmid, at the time of the loss event in question. It does so for each loss event separately, which each yield one point (percentile rank) in the box-plot. The text has been adapted to clarify these points. Furthermore, as the data points, which are used to test if the median percentile rank is significantly different from 50, are nominal (with only 2 categories: the percentiles either fall below 50 or above it), we do not believe normality of the data is required.

2. A necessary but notable caveat of the work is the use of an inducible promoter to drive Cas operon expression. These promoters create a somewhat artificial setup where Cascade is being expressed and forming while the invader is present in the cell. The use of inducers (one metabolizable) with associated uptake and catabolic pathways could introduce fluctuations not associated with constitutive promoters. I fully recognize that a constitutive promoter couldn't be used under this experimental setup, although the use of an inducible promoter does limit how broadly conclusions can be drawn about the dynamics of the experimental system.

The arabinose is not metabolizable by our strain due to the deletion of araBAD. We have now added this in the strain description (Appendix Table S1) and added a reference to Datsenko et al. 2012 where the original strains were created and this mutation is noted.

3. The authors draw some major conclusions purely from the stochastic modeling, including the contribution of target search to the variability in primed interference as well as the role of copy number. The conclusions are stated rather definitely, although there are many reasons why the model could be misleading (e.g., model assumptions, oversimplification, important but missing mechanistic steps, fit parameters in the wrong regime). Any conclusions from the model need to be tempered or supported with experimental evidence. For instance, changing the origin-of-replication to a higher-copy number and repeating some basic experiments could support one of these major conclusions.

We thank the reviewer for this comment. Reading the text again, we agree that some conclusions which are based on the modeling are stated too strongly. We fully agree that any modeling, even when consistent with experimental data, generates hypotheses and can provide mechanistic understanding rather than driving definite conclusions. Hence we have tempered these conclusions as suggested.

While we do not intend for the simulation results to give quantitative predictions of the system, we believe that our model is sufficiently detailed to capture all relevant phases of direct interference and priming in order to provide understanding on a mechanistic level. It is also clear that with modeling one can study aspects that are difficult or impossible to address experimentally, such as systematically varying parameters or separating processes such as primed adaptation and interference.

Nonetheless, motivated by the remarks, we now also report the experimental data that we previously used to calibrate the model at a higher target copy number (~ 50 copies). This has now been added as Appendix Fig S8, and the text has been changed to reflect that this data was to estimate model parameters. The modeling suggests that when moving from the

previous low 5 copies to a higher 50 copies, the mean target loss time for priming should increase. The experimental data (Appendix Fig S8c), shows that the plasmid loss time (PLT) distribution has indeed shifted to larger values. When considering a quantitative comparison of the mean PLT with the model, one should note that the experimental distribution is capped by the duration of the experiment (25 hours). However, one can compare the experimental and theoretical distributions directly, which are observed to show a good agreement in the measured time window (Appendix Fig S8d). Hence, these data support our assertion that increased target copy numbers lead to increased PLT for primed adaptation. For direct interference, our predictions of the PLT for cells with higher target copy numbers agree qualitatively with those from a preprint manuscript from Mamontov et al. 2021 (Fig 6 of their manuscript).

Other comments:

4. L. 51-52: The PAMs recognized by the I-E system are far broader than CTT. Slight rewording here could convey (or at least not contradict) this point.

The authors thank the reviewer for pointing this out and have adapted the text in to read "For type I-E systems a 5'-CTT consensus PAM (Protospacer Adjacent Motif) sequence flanking the targeted site of the invader (Deveau et al., 2008; Mojica et al., 2009) allows swift recognition and ultimately degradation of the invader, through a process called direct interference." to ensure it does not imply this is the only recognized PAM.

5. L. 64, 331: I don't think tug-of-war is the right analogy, as it applies to similar entities trying to undermine each other. Instead, the analogy of a race would be more appropriate, where which proceeds faster determines the final outcome (invader propagation or clearance).

The text has been changed in these two places to replace the tug-of-war analogy.

6. L. 139: this statement isn't quite true, as CRISPR-Cas was present under both experimental conditions. Instead, you could claim that the effect was target-dependent. To claim CRISPR-Cas dependence, either would have to be removed while keeping the target present.

The text has been adapted to read "When the plasmid did not contain a target sequence (pControl) YFP levels did not decrease for over 35 hours (Fig EV1A), indicating targeting by CRISPR-Cas is required for plasmid loss in this setup." to incorporate this point

7. L. 142: avoid using "significant" unless statistical comparisons are being made. "substantial" is easy to use instead.

We have changed the text to read 'substantial' to incorporate this point

8. L. 146: I wouldn't say YFP is diluted exponentially. It's more that YFP is diluted in half

with each cell division, so halting YFP production results in the apparent YFP levels to drop exponentially in individual cells over time.

As a cell grows, its cell length increases exponentially while the cell width remains constant. Thus, the concentrations of existing proteins follow an exponentially decreasing curve, and concentrations are not only decreased at the time of cell division.

9. L. 174-175: I'm having a hard time understanding what this sentence is conveying.

We thank the reviewer for pointing out that further explanation was needed. The text has been adapted to read “When aligned at the PLT, the average profile of all production rate traces for direct interference and priming show a similar trend both right before and after plasmid loss is detected (Fig EV1D)” to clarify this point.

10. L. 177-178: could the authors sequence a small number of acquired spacers to show that the associated protospacers are all flanked by the expected CTT PAM? I know this is expected, although the current sentence sets up this experiment

We have sequenced new spacers from priming experiments in these exact strains and typically find more than 95% of new spacers with a CTT PAM. Due to the use of the microfluidic chip it is not possible to directly sequence the spacers acquired by individual cells in the imaged population. Cells end up in the waste and are collected from the entire chip including many wells not imaged. Apart from spacer PCRs which confirmed spacer acquisition in the chip, we feel that sequencing new spacers from the waste would give us the same information as from bulk. Moreover this exact waste is no longer available for analysis. New approaches with optical tweezers may in the future enable collecting individual cells from wells, and sequencing their new spacers specifically, which would be of interest.

11. L. 201-203: I had trouble getting through this sentence as well.

We have adapted the section between to clarify the meaning of this sentence.

12. L. 204: are the fractions equal?

Yes, the data is binned, so each observation falls into one of five equal bins: 0-0.2, 0.2-0.4, etc. We have replaced the text with “five equal fractions” to clarify this.

13. L. 212-213: provide some explanation (or a reference) why ranking analysis suppresses long-term treds.

The text has been modified between to provide more explanation for the use of the ranking analysis and how it circumvents the effects of the long-term trends in non-stationary cell features due to environmental changes on the correlation

14. L. 230-232: causality is questionable here, as engaging acquisition could be slowing growth.

We agree with the reviewers point that our study has a limited ability to prove either as the sole cause. However, meanwhile there has been a publication by Dimitriu et al. showing a causal link between slow growth induced by bacteriostatic antibiotics and heightened spacer acquisition. Therefore we believe that this is the most likely explanation. We have adjusted the text in the manuscript to give both options and indicated the explanation we find most likely.

15. L. 251: provide a p-value similar to the prior sentence.
The text has been adapted to include the p value of 0.32 to incorporate this comment.

16. L. 255: explain what Cascade search hours are, as this term hasn't been introduced and is not clearly linked to the analyses in this section.

We thank the reviewer for pointing out the briefness of this section and have adapted the text to read "Hence, to test this notion, we investigated the correlation between Cascade search hours and the PLT for each lineage at time points onwards from induction. Cascade search hours can be described as the sum of hours spent by all Cascades in the cell searching for the target and are determined from the cumulative RFP, i.e. the area under the RFP concentration curve of each lineage from induction until a point of interest (Appendix Fig S7). One may expect that a single-cell lineage which has a high number of Cascades for a short period of time close to induction could undergo adaptation earlier than a cell which has a lower number of Cascades over a longer period of time, or vice versa (Appendix Fig S7)." to try and clarify what is meant by the Cascade search hours.

In addition, we have now added an Appendix Fig S7 to help the reader with this concept.

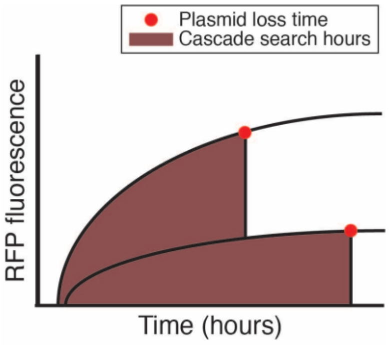

17. L. 258-259: I'm having trouble understanding how the correlation increases in magnitude but is not significant. Some rewording would be helpful here.

We agree with the reviewer that this was confusing and modified the text to read "For the priming process, the impact of Cascade levels appeared weaker and no significant correlation was found between PLT and Cascade concentration prior to loss (Fig 4d)" as we believe we could not detect such an effect well in our set up.

18. L. 275-276: was the reduction in binding affinity through parameter fitting? If so, this should be clearly stated rather than generally saying the binding affinity was found to be lower.

In the model, we can only reproduce the priming data if we reduce the binding affinity of Cascade to the target by 2 orders of magnitude while leaving the other parameters intact (this is shown in Fig. 5E-H). We have adapted the text to reflect that the value of the reduction was found through parameter fitting.

19. L. 344-346: alternatively, cells with fewer plasmids have a lower resource burden and may divide more quickly.

The authors thank the reviewer for this alternative hypothesis however, we believe based on a comparison of the growth rate in plasmid-containing cells and plasmid-free cells from our data that the resource burden of 5 copies of the plasmid in our setup is not large enough to create such an effect. It is possible however, in a setup where a high copy number plasmid is used such an effect would be relevant and easily detectable.

20. L. 375-377: I'm not sold on this line of argumentation. If anything, a highly-susceptible subpopulation could greatly increase phage titers, even if other cells are more efficiently clearing phages that do infect.

We thank the reviewer for this point of view and agree that this could also be the case. We have added this point to the discussion which now reads. "Further, we cannot discount that a more susceptible subpopulation of cells may lead to higher overall phage titres and a larger overall threat to the population."

21. Figure 1C: include the blue segment of the target plasmid on the left. Otherwise, it looks like the plasmid is changing (versus CRISPR picking up a new spacer that can target a different location in the plasmid).

We thank the reviewers for pointing this out Fig. 1C has been modified accordingly.

22. Figure 2C: Change CT to a different color, unless the authors intend for it to be linked to fluorescence concentration.

We have changed CT to green in Fig. 2C.

23. Figure 3 legend: provide more descriptions, such as the important labels in panel a, what the black dots represent in panel b, or what's meant by loss of cells.

In order to clarify what is shown we have adapted the legend of Fig. 3A&B to read "**A** Schematic of key analysis structure and terminology. Closed circles indicate cells which clear the plasmid before division, open circles indicate cells which have not yet lost the plasmid. **B** A comparison of 9 subtrees constructed from induction. The subtrees consist of reconstructed fluorescence lineage traces (grey). Loss lineages are indicated in purple with

the PLTs indicated by closed black dots."For figure 3C the axis label has been adapted to "Fraction of cells that lost the plasmid".

24. Figure 3: what's the difference between growth rate and interdivision time? I would assume that one is merely the inverse of the other.

Growth rates are determined by fitting an exponential function to recorded cell lengths over multiple frames and thus represent the rate of cell elongation, whereas interdivision time is calculated as the time between subsequent divisions. We have added this information to the Methods section.

In individual cells, growth rate and interdivision time cease to be equivalent quantities. While on average growth rate and interdivision time are linearly related, cells with identical growth rates do not necessarily have the same interdivision time. This is illustrated for example in Fig. 4 of Kennard et al., 2016 (DOI: 10.1103/PhysRevE.93.012408).

25. Figure 4 title: this appears incorrect. It's a rephrased version of the Figure 3 title and doesn't address Cascade levels.

We thank the reviewers for identifying this mistake. The figure title has been adapted to **"Fig. 4| Cellular Cascade concentrations influence the direct interference response"**

26. Figure 4b: there should be a unit in the numerator (e.g., molecules).

The authors have adapted the y-axis label description to Cascade molecules/ μm^2, and modified the figure caption to clarify the significance of the unit in relation to cell area.

27. Figure 5: add a schematic depicting the key steps of the model. That way the reader can have some understanding of how direct and primed interference are captured without having to dig deeply into the SI methods.

We have added Figure EV5 which shows key aspects of the agent-based model, as well as a schematic which depicts the (simplified) mechanisms of direct interference and priming.

Reviewer #3:

Summary

McKenzie et al. establish an elegant system to monitor the activity of the E. coli type I CRISPR-Cas system in live single cells. The authors utilize two experimental setups to study interference and primed acquisition and find that while interference occurs on a similar timeframe in most cells, the timing of primed acquisition is highly variable. Most intriguingly slow-growing cells are better able to conduct priming. The authors use modeling to hypothesize that priming stochasticity owes to low-affinity binding of Cascade to target DNA and that stochastic CRISPR-Cas expression can accelerate priming.

General remarks

The microscopy and analysis is rigorously conducted allowing the authors to gain new insights into the single-cell biology of CRISPR-Cas defense. In the second half of the paper, the authors use modeling to generate several hypotheses regarding primed adaptation, and the paper would benefit significantly from experiments that test these hypotheses. The paper is presented as the first of its kind, however, at least one other study has used time-lapse microscopy to study the dynamics and variability of type I CRISPR-Cas defense (Guan, Quant Biol, 2017). This work should be cited and discussed in the authors introduction and discussion, both in terms of background/context and in how the authors methodologies and results compare. Nonetheless, there are several technical and conceptual advances, including the use of an inducible CRISPR-Cas system to monitor plasmid loss and the relationship between CRISPR-Cas activites and cellular properties, such as growth rate. This work will be of interest to the CRISPR-Cas field, bacterial cell biologists and microbiologists interested in phage defense.

We would like to thank the reviewer for the critical reading of the manuscript, the positive feedback and the comments that have enabled us to improve our work.

In addition we thank the reviewer for pointing out the work of Guan et al 2017 we now reference this work in both the introduction and discussion. Further, the discussion now compares the work of Guan et al. to our own in the section that reads "For direct interference, we found our observed degradation time of ~ 90 mins on average for the consensus target plasmids to be on a comparable time scale to previous work (Guan et al, 2017) considering the differences in set up including but not exclusive to having less targeting spacers, a higher copy number of targets and differences in induction levels of the Cas proteins. While we saw, in agreement with the work of Guan et al. 2017, that cell size was not an influential factor in the speed of target degradation, we additionally found that cells that cleared the target DNA earlier, grew and divided faster than the population mean."

Major points

As stated above, this paper would be strengthened by experimental validation of the hypotheses generated by modeling:
- Binding affinity drives priming variability - can the authors test alternative PAM/seed escaper mutants with distinct target affinities? Even in the absence of known affinities, testing a seed escaper as an alternative to the PAM mutant already tested would add depth to the paper.

We agree that more experimental data could be beneficial. However, this goes beyond the scope of this paper as addressing these new modeling hypotheses experimentally would take several months to complete for each new variant, and we currently do not have the trained personnel to complete such studies. We therefore feel that the comparison of priming against different CRISPR escape mutants would be better addressed in a next study, while this study only presents the hypothesis based on the modeling.

- Target abundance affects the balance between primed adaptation and primed interference - can the authors test plasmids with different copy numbers and/or different numbers of targets on the same plasmid?

In response to this comment and a similar remark by reviewer 2, we now also report the experimental data that we previously used to calibrate the model at a higher target copy number (~ 50 copies). This has now been added as Appendix Fig S8, and the text has been changed to reflect that this data was to estimate model parameters.

Since we are not able to detect the moment of spacer acquisition in individual lineages due to natural fluctuations in the production rate, it is not possible to quantify the time necessary for adaptation and interference. However, the overall distribution of plasmid loss time that was observed in the experiment with the higher target copy number showed good agreement with our predictions based on the model. The new Appendix Fig S8 shows that the plasmid loss time distribution has indeed shifted to larger values. When considering a quantitative comparison of the mean PLT with the model, one should note that the experimental distribution is capped by the duration of the experiment (25 hours).

The observation that slow cell growth is associated with cells that will ultimately undergo primed adaptation is fascinating. However, I am not sure that this conclusion is fully supported by the data.
- The major question is whether slow cell growth truly precedes adaptation and describes a cellular state which is more predisposed to adaptation or whether the act of adaptation is itself slowing cells down, and this is bleeding into the growth rate calculation. One may assume that adaptation could slow cells down due to broken DNA (substrates for adaptation) causing a DNA damage response. The authors use a lookback window of 90-60 minutes before PLT because adaptation occurs on average 60 minutes before plasmid loss. However, if this is the average, then many individual cells will be undergoing adaptation >60 mins before PLT which will be during and not after the window. Wouldn't it be better to set the lookback window on a cell-by-cell basis tied to the moment that fluorescence begins to decrease, which is an event more proximal (and therefore less variable) to adaptation than PLT? If this is not possible, how can the authors be sure that they are measuring events occurring before and not during spacer acquisition?

While perhaps clear, we would like to point out that we do set the window on a cell-by-cell basis, and that the PLT, which is based on the fluorescence production rate, is effectively one of the best measures of the moment the fluorescence decreases. This can for instance be seen in the various lineages in Fig. 3B, with the dots which represent the PLT positioned at a moment when the fluorescence decreases. The rest of the decrease is rather to dilute the proteins out by volume dilution.
We have now expanded the discussion to further elaborate on the reasons we believe slow growth may have a positive effect on the speed of spacer acquisition and therefore priming, including a cellular stress response due to DNA damage.

While the reviewer’s concern about the lookback window preceding the moment of spacer acquisition is valid, it is not possible to detect the exact onset of plasmid clearance in individual lineages due to natural fluctuations in the production rate. However, as we have

found that clearance after adaptation is similar to direct interference, we expect that the variability present in the primed interference process is similar to the direct interference distribution. Thus, we expect that variation in the time required for primed interference is relatively small, and the lookback window will thus mostly precede the moment where adaptation is expected to happen. Additionally, the autocorrelation time of the growth rate is approximately 30 minutes (Appendix Fig S3), and we thus expect that the effect of having the window a few minutes too early or too late will be relatively small.

- If the authors are instead suggesting that slow growth is caused by the act of spacer acquisition (or substrate generation) and is therefore not indicative of a cellular state that is predisposed to undergo priming, this is a less interesting but still novel conclusion. Regardless, the authors should more thoroughly distinguish between these models (slow growth before priming vs. slow growth during priming) and discuss which precisely they are able to test.

We thank the reviewer for the feedback and input to help more clearly define this finding. We believe that slow growth precedes and aids the adaptation process possibly due to a number of factors including a higher target plasmid copy number, a possible increase in the concentration of Cascade and potentially the increased time for the integration process to occur without interruption. In line with this idea, recently published studies on primed CRISPR adaptation in I-F systems in *Pseudomonas aeruginosa* have found a causal link between reduced bacterial growth rates by bacteriostatic antibiotics and increased spacer acquisition (Dimitriu et al, 2022). This finding was attributed to slower phage development, allowing more time for cells to acquire spacers. We acknowledge that our setup does not have the ability to eliminate the other hypothesis that spacer insertion events itself can lead to slower growth. To this end, we have added both options to the discussion and indicated our preferred explanation.

This section now reads "For priming the reverse was found. Cells that adapted and cleared the target DNA earlier, grew more slowly than the population mean. From this we conjectured that slower growth may lead to more spacer acquisition events, or that spacer acquisition may cause slower growth of cells. We speculate that the first explanation is most likely explanation may play a role due to copy number maintenance mechanisms, which result in higher concentrations of target plasmids in slow growing cells (Ingmer et al, 2001). This hypothesis was further supported by the model which showed that adaptation occurs more efficiently in the presence of a higher number of targets . While in our set up an effect of Cascade concentration on priming could not be detected, we note that slower growing cells had higher Cascade abundance (Fig EV4) suggesting that Cascade levels may play a role in combination with other factors enhanced by slow growth. Slow growth may simply provide more time to the cell to locate the target and facilitate spacer insertion before interruption by cell division (Høyland-Kroghsbo et al, 2018). In line with this idea, recently published studies on primed CRISPR adaptation in I-F systems in *Pseudomonas aeruginosa* have found a causal link between reduced bacterial growth rates and increased spacer acquisition (Dimitriu et al, 2022). This finding was attributed to slower phage development induced by bacteriostatic antibiotics, allowing more time for cells to acquire spacers especially in the late-exponential and early-stationary phases, when cells are presumably

growing slower (Amlinger et al, 2017). Our findings together with these studies indicate that slow growth caused by any environmental change or cellular stress may in fact be beneficial to a cell trying to undergo adaptation."
Conversely, due to our limited ability to determine the precise moment acquisition occurred, we cannot rule out that slow cell growth could be occurring as a result of the spacer integration process itself resulting in a cellular stress response due to DNA damage. Therefore we have also discussed this option in the revised manuscript.

- In 3F, how can the cells show a reduced growth rate with an unchanged size and division time? If cells are growing more slowly but maintaining the same size, doesn't this mean that they must be also dividing more slowly (i.e. have a longer interdivision time?)

For population averages this is indeed correct: cell populations that grow slower, e.g. because they are in less rich media, strictly have larger interdivision times and are smaller in size. Here however, individual cells here rather grow slower due to internal stochasticity, when the external conditions are constant. Such growth fluctuations are transient, and can occur within one cell cycle. Hence, variations in growth rate within the lookback window (which is calculated over a part of a cell cycle) are not necessarily correlated with interdivision time (which is calculated over the entire cell cycle) or size (which anyway varies by a factor two due to doubling).

Moreover, as internal stochasticity drives variability in all these quantities, their relations are not strict but rather correlations. For instance, it is possible for a cell to have a lower growth rate the entire cycle without a longer interdivision time, as that cell in that cycle can happen to become smaller. More specifically, it has been found that while there is some correlation between initial size at the start of the cell cycle and growth rate over the entire cell cycle, this correlation is not large (Kennard et al., 2016). For direct interference, this negative correlation was also detected in our data. Overall, these observations indicate that cells can transiently show a reduced growth rate while without a concomitant reduction in size and interdivision time.

- In Sup Fig. 17, it is mentioned that slow cell growth is associated with higher Cascade concentrations. Could this be why slow growth is associated with priming? Can you control for Cascade expression and determine if slow cell growth is still associated with priming?

This is a very interesting suggestion. It is indeed possible that transiently slower-growing cells have rising cascade levels (for instance due to limited volume growth), which in turn accelerates plasmid clearance. We now address this possibility in the discussion. Following the suggestion, we controlled for Cascade expression, and checked whether growth was then associated with priming. We did not find significant correlations. However, due to the limited size of the data-set, it is well possible that there is an underlying correlation but it was not detectable. Hence, we cannot base a specific conclusion based on this analysis. Overall however, we do think that this is a possible explanation. Additionally, we believe that the increased target copy number associated with slower growing cells could also increase the probability of successful adaptation.

- The discussion should elaborate on why the authors believe slow growth is associated with priming. There are several possibilities (DNA damage, some other stress, high Cascade concentrations, something else) and the authors should speculate on what exactly they think is going on. Line 438 "perhaps due to higher target DNA availability" may allude to the DNA damage hypothesis, but it unclear to me what exactly is meant as written.

We have now expanded the discussion to further elaborate on the reasons we believe slow growth may have a positive effect on the speed of spacer acquisition and therefore priming.

Minor points

End of intro (99-103): mention that these results were based on modeling, as written they sound like experimental conclusions.

The text has been adapted to read “Further, through stochastic modeling we were able to define the adaptation and clearance stages of priming and identified primed adaptation as the source of the variation observed – more specifically the binding of Cascade to the mutated target DNA, rather than other complex processes including the integration of new spacer DNA fragments into the host genome” to incorporate this point

206-207: I'm struggling to understand why the moment of loss wouldn't be more biased toward earlier stages of the cell cycle when the adaptation event occurred in the parent - causing both sisters to lose the plasmid.

We believe that adaptation in the parent could occur at any moment during the cell cycle. As this will then be followed by the direct interference process which showed only a small amount of variation, we would expect for daughter cells in which the mother incorporated a spacer at the beginning of the cell cycle to clear the plasmid much earlier in the cell cycle than daughter cells whose mother incorporated a spacer just before division. As the moment of adaptation is random we therefore expect plasmid loss to be randomly distributed for different pairs of daughter cells based on when adaptation occurred. We have now adapted the text to try and clarify this point.

Fig. 3F: Why doesn't the data in 3F (30 minute lookback window) match the data in Sup Fig. 9 (top left)? Is this different data? Does the "30 minute lookback" in the 3F legend refer to something other than wstart = 30 mins and wend = 0 min? If the data in 3F refers to wstart = 90 mins and wend = 60 mins (my guess from line 227), then this should be stated as such in the lIf we need to reduce the legend

We thank the reviewer for pointing this out. In Appendix Fig S5 we used a different lookback window wstart 30 min prior to plasmid loss and wend 0 min. As you have identified in figure 3f the window wstart 90 wend 60 is used. For clarity we have added the window start and end for the panels of 3f to the figure legend.

216: I would prefer that the lookback window is described in some more detail here instead of saying "determined using autocorrelation times" which won't be meaningful to many readers. Can you explain what lookback window was used for Fig. 3F and why?

We have adapted the text to clarify the significance of the autocorrelation time and how it has been used to determine the lookback window.

It is unclear to me how growth rate was calculated. This should be described somewhere in the methods, unless I missed it.

Growth rates are determined by fitting an exponential function to recorded cell lengths over multiple frames and thus represent the rate of cell elongation. We have added this detail to the Methods section (lines 566-569).

247: Figure legend says 30 min lookback window, text says 60 min lookback window

We thank the reviewer for pointing this out. This has been changed in the figure 4 legend as a 60-minute window was used.

252-253: Doesn't this statement need to refer to a figure or paper?

Thank you for pointing this out. Indeed, we were referring to our own findings, a reference to Fig 2F has been added.

255-259: I tried to read this several times and cannot follow. Can you expand this section in order to clarify the methods and interpretations? How are Cascade search hours calculated? Can you add a few "suggesting that..." phrases to really hammer home what the data is telling you?

We thank the reviewer for pointing out this section needed further improvement. We have now expanded this section of the paper to address this comment and clarify the methods and findings.

283: "by a factor of 100"
284: find > found

The above two points have been corrected in the text

Final section 310-324: I had to read this section several times. What was motivation for changing the variation of Cascade expression? Is this relevant to some natural aspect of Cascade expression? The last sentence is a bit anti-climactic and refers elsewhere for a more thorough explanation. Can this be expanded upon in the results section, specifically "the highly non-linear fashion"? Is there a figure that can be pointed to to demonstrate this?

We thank the reviewer for pointing out that this section needs additional context. We have expanded on our motivation for doing this analysis, and have included a more detailed explanation of our findings.

497: 'was performed 1ul' > 'was performed with 1 ul'
623: 'resulting in' > 'results in'
624: 'To invoke priming the' > 'To invoke priming, the'
631: 'To allow long term imaging cells' > 'To allow long term imaging, cells'
672/673: 'Depict clearance' > 'Clearance'
762: 'integrated upstream of the cas8e subunit' > 'fused to the N terminus of the Cas8e subunit'

The authors would like to thank the reviewer for their attention to detail the 6 points listed above have been adapted in the text

FIGURES
We thank the reviewers for their sharp eye on the figures. All comments have been changed in the figures or adapted in the text as below

Fig 1 - Panels a & c: Label the promoters, or describe them in the figure legend, so we know which are native/inducible (and arabinose vs IPTG inducible) without checking the methods section. Also, Cas3 and Cas1, 2 in locus diagram should be lower case/italicized.

The promoters have now been further described in the figure legend "The cell contains a I-E CRISPR-Cas system controlled by the lacUV5 (cas3) and araBp8 (cas operon) inducible promoters (black arrows)" and the cas genes have been italicized

Fig 2D/G - Label the x axes 'PLT' instead of 'Time.' This would keep the figure consistent with panel descriptions, and distinguish these graphs from those in panels b & f (where they use 'Time' to refer to time after induction, rather than PLT). Another minor note, but I kind of want the y axes scale (percentage) to be the same in both graphs to allow for direct comparison.

We thank the reviewer for this suggestion and agree it better clarifies what the plot shows and aligns with the legend. The figure panels have been adapted to incorporate the suggested changes.

Fig 2F: Why does fluorescence decrease sharply at the beginning? Is that due to dilution following cell divisions? Photobleaching of initial YFP population?

Yes, we believe it to be both due to photo bleaching as we see the YFP reaches a new equilibrium after ~ 4 hours. We correct for this when calculating the production rate by adding a bleaching rate into the equation to correct for a faster bleaching rate when there is more YFP present.

Fig 3A: Describe the difference between open and closed circles. I think closed circles are cells in which plasmid the was lost (if same as closed circles in b), but it would be nice if they spelled that out.

The legend has been updated to read "A Schematic of key analysis structure and terminology. Closed circles indicate cells which clear the plasmid before division, open circles indicate cells which have not yet lost the plasmid."

Fig 3B: Again, would like x axis labeled PLT. Also, it's not intuitive what exactly what is being shown and it is not described in the legend.

The authors have changed the legend to read "**B** A comparison of 9 subtrees constructed from induction. The subtrees consist of reconstructed fluorescence lineage traces (grey). Loss lineages are indicated in purple with the PLTs indicated by closed black dots." However, we have not changed the x axis to PLT as time (h) is more relevant for the fluorescence traces, the PLTs calculated from the production rate are indicated by the black dot and form only a small subset of all data points shown.

Fig 3C - priming: are the colors swapped?

Yes thanks for this observation, this has been corrected.

Sup Fig 2 legend - consider mentioning that this is for a representative individual lineage?

We have adapted the figure legend to clarify the fluorescence data comes from a single representative lineage.

Sup Fig 4 title: 'during Direct interference' > 'during direct interference'

Thanks, this has been corrected.

Sup Fig 6 description: What is meant by 'the moment of detection' (filled red dots)?

The red dot indicates the PLT detected using the production rate. For clarity the legend has been adapted to read "Lineages that were able to clear the plasmid are highlighted in blue with the red dot indicating the PLT determined by the production rate".

Sup Fig 5 - Can you put the lane information above each lane instead of referring readers to the legend to see what each lane is?

This has been adapted.

Sup Fig 6 - How does this compare to a control in which there is no interference? Is there 0% plasmid loss in this case?Also, the fluorescence curves look like they're on a sharp decline even before the plasmid loss event. Shouldn't the loss be more gradual given that each cleavage event (as the copy number decreases) is significantly impaired relative to

interference with a perfect PAM? In other words, how does the rate of fluorescence decrease in those rare lineages shown compare to those during perfect interference?

The authors believe that such a control is given in Fig EV1A. where the CRISPR system has no target and is unable to clear the plasmid. In this control we see no loss of the plasmid in 36 hours so believe the plasmid is unlikely to be lost in the absence of interference. In addition, the pSC101 origin was chosen as it has stringent copy number control making it more difficult for the plasmid to be lost in a CRISPR-independent or interference-independent way.

Indeed, the signals seem to show a sharp decrease, and one may expect plasmid clearance to be much slower here. First, it is important to note that the measured decreases in YFP concentration are dominated by dilution due to volume growth: when the cells double in size, the YFP concentration halves, but this is true when no new YFP is made, which may not be the case during that initial decrease. It is not trivial / possible to extract from the data the underlying cleavage events or copy number decreases, as is likely clear to the reviewer, because of the many contributing events (not only plasmid cleavage, but also plasmid replication, YFP production, and YFP dilution). For this reason it is better to consider the YFP production rate for this purpose. While the plasmid clearance may be 30 minutes longer, we do not see a major increase in the plasmid loss time. However, for the control experiment shown in Fig EV1C there were only five observations of plasmid loss. Unfortunately this number is too small to draw any quantitative conclusions about the steepness of the production rate curves between the Priming and the control data.

3rd Mar 2022

Manuscript Number: MSB-2021-10680R
Title: Single Cell Variability of CRISPR-Cas Interference and Adaptation
Author: Rebecca McKenzie
Emma Keizer
Jochem Vink
Jasper van Lopik
Ferhat Büke
Vera Kalkman
Christian Fleck
Sander Tans
Stan Brouns

Dear Dr. Brouns,

Thank you for sending us your revised manuscript. We have now heard back from the two reviewers who were asked to evaluate your study. As you will see, the reviewers are overall satisfied with the modifications made and think that the study is now suitable for publication.

Before we can formally accept your manuscript, we would ask you to address the following issues:
1. the remaining comments of Reviewer #2.

On a more editorial level:

Thank you for submitting this study to Molecular Systems Biology. I look forward to receiving your revised manuscript soon.

Kind regards,
Jingyi

Jingyi Hou
Editor
Molecular Systems Biology

If you do choose to resubmit, please click on the link below to submit the revision online before 2nd Apr 2022.

https://msb.msubmit.net/cgi-bin/main.plex

IMPORTANT: When you send your revision, we will require the following items:
1. the manuscript text in LaTeX, RTF or MS Word format
2. a letter with a detailed description of the changes made in response to the referees. Please specify clearly the exact places in the text (pages and paragraphs) where each change has been made in response to each specific comment given
3. three to four 'bullet points' highlighting the main findings of your study
4. a short 'blurb' text summarizing in two sentences the study (max. 250 characters)
5. a 'thumbnail image' (550px width and max 400px height, Illustrator, PowerPoint or jpeg format), which can be used as 'visual title' for the synopsis section of your paper.
6. Please include an author contributions statement after the Acknowledgements section (see https://www.embopress.org/page/journal/17444292/authorguide#manuscriptpreparation)
7. Please complete the CHECKLIST available at (https://bit.ly/EMBOPressAuthorChecklist).
Please note that the Author Checklist will be published alongside the paper as part of the transparent process (https://www.embopress.org/page/journal/17444292/authorguide#transparentprocess).
8. When assembling figures, please refer to our figure preparation guideline in order to ensure proper formatting and readability in print as well as on screen:
https://bit.ly/EMBOPressFigurePreparationGuideline
See also figure legend guidelines: https://www.embopress.org/page/journal/17444292/authorguide#figureformat
9. Please note that corresponding authors are required to supply an ORCID ID for their name upon submission of a revised manuscript (EMBO Press signed a joint statement to encourage ORCID adoption).
(https://www.embopress.org/page/journal/17444292/authorguide#editorialprocess)
Currently, our records indicate that the ORCID for your account is 0000-0002-9573-1724.

Please click the link below to modify this ORCID:
Link Not Available

The system will prompt you to fill in your funding and payment information. This will allow Wiley to send you a quote for the article processing charge (APC) in case of acceptance. This quote takes into account any reduction or fee waivers that you may be eligible for. Authors do not need to pay any fees before their manuscript is accepted and transferred to the publisher.

As a matter of course, please make sure that you have correctly followed the instructions for authors as given on the submission website.

*** PLEASE NOTE *** As part of the EMBO Press transparent editorial process initiative (see our Editorial at https://dx.doi.org/10.1038/msb.2010.72 , Molecular Systems Biology will publish online a Review Process File to accompany accepted manuscripts. When preparing your letter of response, please be aware that in the event of acceptance, your cover letter/point-by-point document will be included as part of this File, which will be available to the scientific community. More information about this initiative is available in our Instructions to Authors. If you have any questions about this initiative, please contact the editorial office (msb@embo.org).

Reviewer #2:

I had two remaining points that could be better addressed, although these can be tackled with slight rewording. Otherwise, the

authors have sufficiently addressed all other comments.

Reviewer #2, Comment #1: the authors state that normality of the data is not required, which I agree with. The issue though is that (unless I'm mistaken) a binomial distribution is making an assumption about the distribution. It's probably reasonable at the end of the day, although it would behoove the authors to state the caveats of this assumption when interpreting their data and the statistical analyses.

Reviewer #2, Comment #8: I'm not sure the length of the cell is increasing exponentially or-more importantly-this increase accounts for the dilution of YFP over time. Instead, as a cell doubles to two cells and then to four cells and so on, a fixed amount of YFP is diluted 2-fold and then 4-fold and so on, creating an exponential reduction in the YFP concentration. See PMID = 15790834 for one of the original examples of these sort of analyses and a relevant paper to cite for this sentence. This will be a small wording change at most; my goal is to make sure the underlying concept is correct.

Reviewer #3:

The authors addressed my concerns in their revised manuscript. I recommend for publication.

Reviewer comments

Reviewer #2:

I had two remaining points that could be better addressed, although these can be tackled with slight rewording. Otherwise, the authors have sufficiently addressed all other comments.

Reviewer #2, Comment #1: the authors state that normality of the data is not required, which I agree with. The issue though is that (unless I'm mistaken) a binomial distribution is making an assumption about the distribution. It's probably reasonable at the end of the day, although it would behoove the authors to state the caveats of this assumption when interpreting their data and the statistical analyses.

> We have now updated the legend of Figures 3 and 4 to include and discuss the assumptions that were made in performing the underlying the statistical test.

Reviewer #2, Comment #8: I'm not sure the length of the cell is increasing exponentially or- more importantly-this increase accounts for the dilution of YFP over time. Instead, as a cell doubles to two cells and then to four cells and so on, a fixed amount of YFP is diluted 2-fold and then 4-fold and so on, creating an exponential reduction in the YFP concentration. See PMID = 15790834 for one of the original examples of these sort of analyses and a relevant paper to cite for this sentence. This will be a small wording change at most; my goal is to make sure the underlying concept is correct.

> While the total amount of YFP per cell indeed halves upon division, the YFP concentration is not affected by the moment of division and rather gradually decreasing due to volume growth. To make this point clear, we have now also show this exponential decrease of the amount of fluorescence after plasmid clearance in Fig EV1B.

Reviewer #3:

The authors addressed my concerns in their revised manuscript. I recommend for publication.

31st Mar 2022
Manuscript number: MSB-2021-10680RR
Title: Single Cell Variability of CRISPR-Cas Interference and Adaptation

Dear Dr. Brouns,

Thank you again for sending us your revised manuscript. We are now satisfied with the modifications made and I am pleased to inform you that your paper has been accepted for publication.

*** PLEASE NOTE *** As part of the EMBO Publications transparent editorial process initiative (see our Editorial at https://dx.doi.org/10.1038/msb.2010.72), Molecular Systems Biology publishes online a Review Process File with each accepted manuscripts. This file will be published in conjunction with your paper and will include the anonymous referee reports, your point- by-point response and all pertinent correspondence relating to the manuscript. If you do NOT want this File to be published, please inform the editorial office at msb@embo.org within 14 days upon receipt of the present letter.

Should you be planning a Press Release on your article, please get in contact with msb@wiley.com as early as possible, in order to coordinate publication and release dates.

LICENSE AND PAYMENT:
All articles published in Molecular Systems Biology are fully open access: immediately and freely available to read, download and share.

Molecular Systems Biology charges an article processing charge (APC) to cover the publication costs. You, as the corresponding author for this manuscript, should have already received a quote with the article processing fee separately.

Once your article is at Wiley for editorial production you will receive an email from Wiley's Author Services system, which will ask you to log in and will present you with the publication license form for completion. Within the same system the publication fee can be paid by credit card, an invoice or pro forma can be requested.

Payment of the publication charge and the signed Open Access Agreement form must be received before the article can be published online.

Molecular Systems Biology articles are published under the Creative Commons licence CC BY, which facilitates the sharing of scientific information by reducing legal barriers, while mandating attribution of the source in accordance to standard scholarly practice.

Proofs will be forwarded to you within the next 2-3 weeks.

Thank you very much for submitting your work to Molecular Systems Biology.

Kind regards,
Jingyi

Jingyi Hou
Editor
Molecular Systems Biology
